# Boron-Insertion-Induced Lattice Engineering of Rh Nanocrystals Toward Enhanced Electrocatalytic Conversion of Nitric Oxide to Ammonia

**DOI:** 10.1007/s40820-025-01919-6

**Published:** 2025-10-05

**Authors:** Peng Han, Xiangou Xu, Weiwei Chen, Long Zheng, Chen Ma, Gang Wang, Lei Xu, Ping Gu, Wenbin Wang, Qiyuan He, Zhiyuan Zeng, Jinlan Wang, Dong Su, Chongyi Ling, Zhengxiang Gu, Ye Chen

**Affiliations:** 1 Department of Chemistry, The Chinese University of Hong Kong, Hong Kong, People’s Republic of China; 2 Key Laboratory of Quantum Materials and Devices of Ministry of Education, School of Physics, Southeast University, Nanjing, 211189 People’s Republic of China; 3 Beijing National Laboratory for Condensed Matter Physics, Institute of Physics, Chinese Academy of Sciences, Beijing, 100190 People’s Republic of China; 4 Department of Materials Science and Engineering, City University of Hong Kong, Hong Kong, People’s Republic of China; 5 Department of Materials Science and Engineering, and State Key Laboratory of Marine Pollution, and Center of Super-Diamond and Advanced Films, City University of Hong Kong, Hong Kong, People’s Republic of China; 6 School of Chemistry and Materials Science, Nanjing Normal University, Nanjing, 210023 People’s Republic of China

**Keywords:** Lattice engineering of nanomaterials, Phase engineering of nanomaterials, Wet-chemical synthesis, Metal nanocatalysts, Nitric oxide reduction reaction, Electrocatalytic ammonia synthesis

## Abstract

Electrocatalytic nitric oxide (NO) reduction reaction (NORR) is a promising and sustainable process that can simultaneously realize green ammonia (NH_3_) synthesis and hazardous NO removal. However, current NORR performances are far from practical needs due to the lack of efficient electrocatalysts. Engineering the lattice of metal-based nanomaterials via phase control has emerged as an effective strategy to modulate their intrinsic electrocatalytic properties. Herein, we realize boron (B)-insertion-induced phase regulation of rhodium (Rh) nanocrystals to obtain amorphous Rh_4_B nanoparticles (NPs) and hexagonal close-packed (*hcp*) RhB NPs through a facile wet-chemical method. A high Faradaic efficiency (92.1 ± 1.2%) and NH_3_ yield rate (629.5 ± 11.0 µmol h^−1^ cm^−2^) are achieved over *hcp* RhB NPs, far superior to those of most reported NORR nanocatalysts. In situ spectro-electrochemical analysis and density functional theory simulations reveal that the excellent electrocatalytic performances of *hcp* RhB NPs are attributed to the upshift of d-band center, enhanced NO adsorption/activation profile, and greatly reduced energy barrier of the rate-determining step. A demonstrative Zn–NO battery is assembled using *hcp* RhB NPs as the cathode and delivers a peak power density of 4.33 mW cm^−2^, realizing simultaneous NO removal, NH_3_ synthesis, and electricity output.

**Supplementary Information:**

The online version contains supplementary material available at 10.1007/s40820-025-01919-6.

## Highlights

- Phase regulation of B-inserted rhodium (Rh) nanocrystals is achieved using a facile wet-chemical approach.
- The B-inserted Rh nanocatalysts exhibit phase-dependent behaviors in electrocatalytic nitric oxide (NO) reduction reaction.
- The hexagonal close-packed RhB nanocatalysts demonstrate superior electrocatalytic activity in NH_3_ production with a maximum NH_3_ yield rate of 629.5 µmol h^−1^ cm^−2^ and FE_NH3_ of 92.1%.
- Theoretical simulations reveal possible origin of the excellent electrocatalytic activity, which could be attributed to the d-band center upshift, enhanced NO adsorption/activation, and reduced energy barrier of rate-determining step.

**Supplementary Information:**

The online version contains supplementary material available at 10.1007/s40820-025-01919-6.

## Graphical abstract

**Figure:**
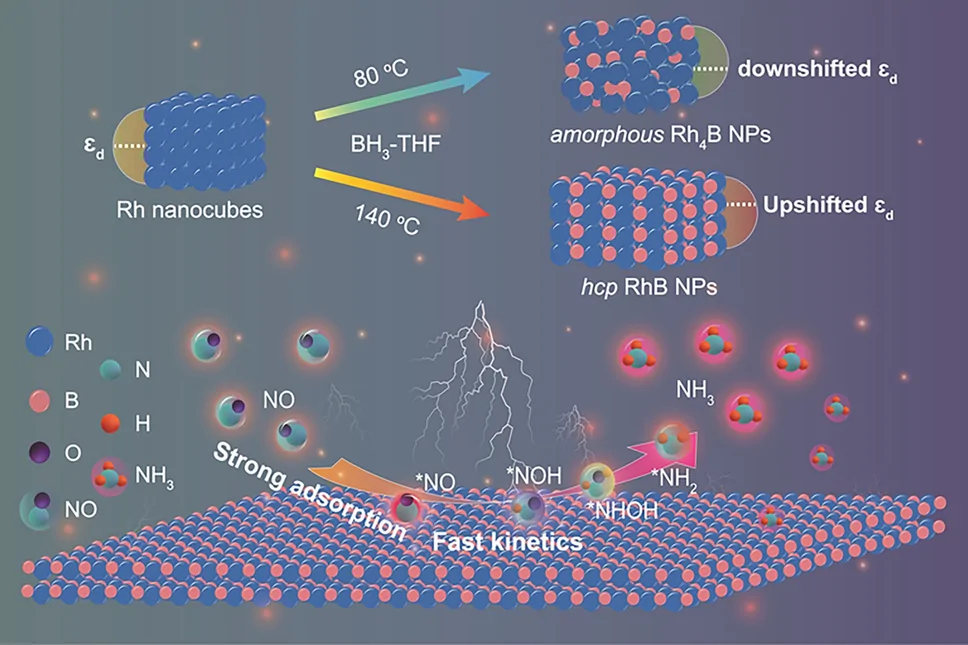

## Introduction

Balance of the nitrogen cycle, a critical biogeochemical process of the global ecosystem, is of great importance for all forms of lives [1, 2]. However, human activities including intensive agriculture, over industrialization, and deforestation have significantly disturbed nitrogen cycle, causing exponentially increasing harmful nitrogen emission [3, 4]. Nitric oxide (NO) is one of the major hazardous nitrogen oxides and oxyanion pollutants, mainly generated from the incomplete combustion of fossil fuels and biomass [5, 6], which could cause respiratory disease [7, 8] and severely damage the ecological environment [1, 8–10]. Therefore, the conversion of discarded NO to harmless N_2_ or even high value-added nitrogenous feedstocks is of great significance. Traditionally, selective catalytic reduction is employed to convert NO to N_2_, but it faces drawbacks of high operating cost and enormous energy consumption due to high reaction temperature and the involvement of valuable reactants such as ammonia (NH_3_) or hydrogen (H_2_) [11, 12]. Therefore, it is urgently needed to develop cost-effective and eco-friendly alternatives for high-throughput removal of NO. Electrocatalytic NO reduction reaction (NORR) uses H_2_O as the hydrogen source to directly reduce NO to NH_3_ under applied potential. Therefore, electrochemical NORR not only offers a sustainable and ambient-condition route for NO conversion, but also alleviates the environmental and energetic burdens of the current Haber–Bosch process by producing green NH_3_ [13–15]. However, the industrial application of electrocatalytic NORR is severely inhabited, largely due to the scarcity of high-performance catalysts with satisfactory activity, selectivity, and durability.

The exploration of NORR electrocatalysts is still in the early stage. Several transition metals [10, 16, 17], metal-based compounds [18–21], and single- or dual-atom catalysts [22, 23] have been reported effective for NORR. Substantial research efforts have been devoted to optimizing their performances via modulating the composition [24], defects [19, 25], facets [15], and surface strain [26]. Recently, phase engineering of nanomaterials emerges as an effective strategy to directly control the atomic arrangement of nanomaterials with tailored functions and compelling performance [10, 27–30]. For instance, cobalt (Co) nanosheets with hexagonal close-packed (*hcp*) phase demonstrated much better NORR activity than the face-centered cubic (*fcc*) counterpart [10]. Among various phase control approaches, incorporating metal nanomaterials with light nonmetal elements like hydrogen (H), boron (B), and carbon (C) shows the uniqueness of simultaneous phase and electronic structure control [31–33]. However, incorporation of such nonmetal species often requires relatively harsh conditions such as high temperature [34] and pressure [35], possibly due to the low reactivity of nonmetal source [36] and high metal-nonmetal bond formation energy [37–39]. Therefore, the development of a facile approach to synthesize novel nonmetal-incorporated metal-based nanocatalysts with controlled phases and tunable electronic structures holds great promise in promoting NORR and enabling in-depth understanding of the phase dependency of metal-based nanocatalysts in electrocatalysis.

Herein, we report the phase-regulated synthesis of B-inserted rhodium (Rh) nanocrystals via a robust wet-chemical method by simply controlling the B-insertion temperature. Lower boronization temperature results in the formation of amorphous Rh_4_B nanoparticles (denoted as *a*-Rh_4_B NPs), while *hcp* RhB NPs are obtained at a higher insertion temperature. Notably, B-inserted Rh nanostructures with different phases exhibit phase-dependent behaviors as NORR nanocatalysts. The *hcp* RhB nanocatalyst achieves remarkable catalytic activity and selectivity with NH_3_ yield rate of 629.5 ± 11.0 µmol h^−1^ cm^−2^ at − 0.60 V vs. reversible hydrogen electrode (RHE) and a maximum Faradaic efficiency (FE) of 92.1% ± 1.2% at − 0.50 V vs. RHE, which are much superior to those of *fcc* Rh nanocubes (NCs) and *a*-Rh_4_B NPs. Furthermore, consecutive cycling electrolysis and long-term chronoamperometry demonstrate the excellent catalytic durability of *hcp* RhB NPs. The projected density of states (PDOSs) and in situ spectro-electrochemical analyses indicate that the unique electronic structure and enhanced NO adsorption/activation capability of *hcp* RhB NPs promote the NO-to-NH_3_ conversion. Moreover, density functional theory (DFT) calculations reveal that *hcp* RhB surface delivers a stronger charge transfer toward NO and possesses lower energy barrier for the rate-determining step (RDS). In addition, the proof-of-concept *hcp* RhB NP-based Zn–NO battery demonstrates a power density of 4.33 mW cm^−2^, superior to most reported Zn–NO batteries. This work not only reveals the significance of lattice engineering in metal-based nanomaterials through B-insertion, but also offers a novel strategy in enhancing the electrocatalytic activity and stability for NORR, paving a new avenue for sustainable NO conversion and NH_3_ production.

## Experimental Section

### Synthesis of *fcc* Rh NCs

Rh NCs were synthesized followed by a previously reported method with minor modifications [40]. In a typical experiment, 52.0 mg AA, 108.0 mg KBr, and 133.0 mg PVP were dissolved in 13.0 mL EG solution in a 50-mL flask. Then, the mixture was heated to 140 °C in oil bath under magnetic stirring (350 rpm) and kept at 140 °C for 0.5 h. After that, 5.0 mL of EG solution containing 46.2 mg Na_3_RhCl_6_ was injected into the above-mentioned mixture, with the first 1.1 mL at a speed of 60.0 mL h^−1^ and the remaining 4.9 mL at a speed of 4.0 mL h^−1^. After the injection, the reaction was maintained at 140 °C for another 3.0 h. The resultant product was collected by centrifugation at 14,000 rpm for 20 min and then washed by a mixture of ethanol and acetone 3 times. Finally, the product was dispersed in 8.0 mL ethanol for further usage.

### Synthesis of ***a***-Rh_4_B NPs

Typically, 1.0 mL of the as-synthesized Rh NC suspension solution was centrifugated at 14,000 rpm and washed by THF for 3 times, followed by redispersion in 0.5 mL THF. After that, this suspension solution and 7.5 mL BH_3_-THF solution were added into a 15-mL pressure tube, which was then purged with high-purity N_2_ gas for 15 min. Then, the tube was sealed and placed in an oil bath and stirred at 80 °C for 3 days. After cooling down to room temperature, the product was collected by centrifugation at 10,000 rpm for 5 min, washed by THF for 3 times, and finally washed by ethanol for 3 times.

The *a*-Rh_4_B NPs can also be synthesized using DMAB as an alternative B source. Typically, 1.0 mL of the as-synthesized suspension solution was centrifugated and washed by THF for 3 times, followed by the redispersion in 5.0 mL THF solution. 300.0 mg of DMAB was mixed with this suspension solution and Further sonicated for 10 min. After the mixture was transferred and sealed into a 15-mL glass pressure vial, it was Further placed into an oil bath, then heated to 110 °C, and kept this temperature for 3 days with a continuous stirring (350 rpm). After cooling down to room temperature, the product was collected by centrifugation and washed by THF for 3 times and ethanol for 3 times, respectively.

### Synthesis of *hcp* RhB NPs

Typically, 1.0 mL of the as-synthesized Rh NC suspension solution was centrifugated at 14,000 rpm and washed by THF for 3 times, followed by the redispersion in 0.5 mL THF solution. After that, this suspension solution and 7.5 mL BH_3_-THF solution were added into a 15-mL pressure tube, which was then purged with high-purity N_2_ gas for 15 min. Then, the tube was sealed and placed in an oil bath and stirred at 140 °C for 3 days. Finally, the products are collected by centrifugation at 10,000 rpm for 5 min, washed by THF for 3 times, and finally washed by ethanol for 3 times.

The *hcp* RhB NPs can also be synthesized using DMAB as an alternative B source, except that the reaction temperature was changed to 170 °C.

### Synthesis of 18.3 nm Rh NCs (Large Rh NCs)

The large Rh NCs were synthesized followed by a previously reported method with some modifications [41]. In a typical experiment, 108.0 mg KBr was dissolved in 4.0 mL EG solution in a 50-mL flask. Then, the mixture was heated to 160 °C under magnetic stirring (350 rpm) and kept at 160 °C for 0.5 h. After that, 4.0 mL of EG solution containing 13.0 mg RhCl_3_·xH_2_O and 36.5 mg PVP was injected into the above-mentioned mixture at a rate of 4.0 mL h^−1^. After injection, the reaction mixture was held at 160 °C for another 15 min and then naturally cooled to room temperature. The products were collected by centrifugation at 12,000 rpm for 5 min and washed by the mixture of ethanol and acetone 3 times. Finally, the product was dispersed in 4.0 ml ethanol for further usage.

### Synthesis of Large *hcp* RhB NPs

The large *hcp* RhB NPs were obtained via the same reaction conditions as that of *hcp* RhB NPs, except using 1.0 mL of the as-synthesized large Rh NCs suspension solution as the starting material instead.

### Preparation of Nanocatalysts Supported on Carbon

To prepare *hcp* RhB NPs/C catalyst, firstly, 8.5 mg of Cabot Vulcan XC-72 carbon was dispersed in 5.0 mL of ethanol and sonicated for 30 min in ice water to form a well-dispersed suspension. Then, 1.5 mL ethanol dispersion containing 1.5 mg of *hcp* RhB NPs was added dropwise into the Cabot Vulcan XC-72 carbon suspension. The obtained mixture was then sonicated for another 1.5 h in ice water. After that, the *hcp* RhB NPs/C was collected by centrifugation at 14,000 rpm for 10 min, followed by washing with ethanol for 2 times and drying at 40 °C at vacuum condition for 12 h. Other catalysts supported on Cabot Vulcan XC-72 carbon were prepared via the same procedure.

### Preparation of Working Electrodes

The catalyst loaded on carbon paper was used as the working electrode. 2.5 mg of the catalysts were dispersed in 2 mL ethanol by sonication for 30 min. 40 µL Nafion solution was added to the mixed solution and sonicated for 30 min to obtain a homogeneous ink. Finally, the ink was loaded dropwise onto carbon paper with an area of 1 × 1.5 cm^2^. The mass loading was calculated to be 1.25 mg cm^−2^. The geometric surface area of the working electrode used in the electrochemical test was 1 × 1 cm^2^.

### Electrochemical Measurements

The electrochemical measurements were taken in a typical H-type cell separated by a Nafion 115 membrane at room temperature and recorded by a CHI760E electrochemical workstation. The electrolyte was 0.5 M Na_2_SO_4_. A graphite rod and the Ag/AgCl electrode were selected as a counter and reference electrode, respectively. The measured potentials via the Ag/AgCl electrode were converted to those based on a reversible hydrogen electrode (RHE) by the Nernst equation:

1 $${\text{E (V vs}}{\text{. RHE)  =  E (V vs}}{\text{. Ag/AgCl)  +  0}}{\mathrm{.0591}} \times {\text{pH  +  0}}{\text{.198 V}}$$

Before the electrochemical measurement, the electrolyte in cathode compartment was purged with Ar flow (25 sccm) for 30 min and then purged with NO flow (15 sccm) for 15 min. During the electrochemical test, Ar or NO was constantly fed at 15 sccm. The linear sweep voltammetry (LSV) curves were established at a scanning rate of 5 mV s^−1^ after 50 cycles of the cyclic voltammetry (CV) at a scan rate of 50 mV s^−1^ to obtain stable curves. Chronoamperometry was used to evaluate the stability under different applied potentials. All data were obtained with 90% of iR-Compensation.

### Calculation Details

The Vienna Ab initio Simulation Package (VASP) was employed to perform density functional theory (DFT) calculations [42]. These calculations used the projector augmented wave (PAW) pseudopotential to approximate the potential generated by the electrons in atoms, incorporating the PBE generalized gradient approximation (GGA) exchange–correlation functional [43, 44]. The Monkhorst–Pack grid was used to set 3 × 3 × 1 k-points in the Brillouin zone for optimizing the structure and 7 × 7 × 2 was used to analyze electron-related properties. In the wave Function calculations, a kinetic energy cutoff of 520 eV and the Gaussian smearing width of 0.05 eV were employed. The z-direction vacuum layer is set to 15 A for all calculations. DFT-D3 method developed by Becke–Johnson was used to model the van der Waals interactions [43, 44]. The relaxation criterion for all atoms, excluding those at the boundary, was met when the residual force fell below 0.01 eV Å^−1^.

To determine the adsorption energy (*E*_ads_) for reactants or reaction intermediates, the following calculation is performed:

2 $${E}_{\mathrm{ads}} =  {E}_{\mathrm{tot}}\text{ - (}{E}_{\mathrm{mol}} +  {E}_{\mathrm{slab}}\mathrm{)}$$

*E*_tot_ represents the total energy of the adsorption system, *E*_mol_ is the energy of the adsorbate molecules, and *E*_slab_ is the energy of the catalyst slab. The calculation of the Gibbs free energy for each species can be expressed as:

3 $$\Delta G = \Delta E + \Delta E_{{{\mathrm{ZPE}}}} - T\Delta S$$

The Gibbs free energy incorporates the zero-point energy change (∆*E*_ZPE_) and the entropy change (∆*S*). For this work, the values of ∆*E*_ZPE_ and ∆*S* were derived from vibrational frequency calculations.

## Results and Discussion

### Material Synthesis and Characterizations

**Fig. 1:**
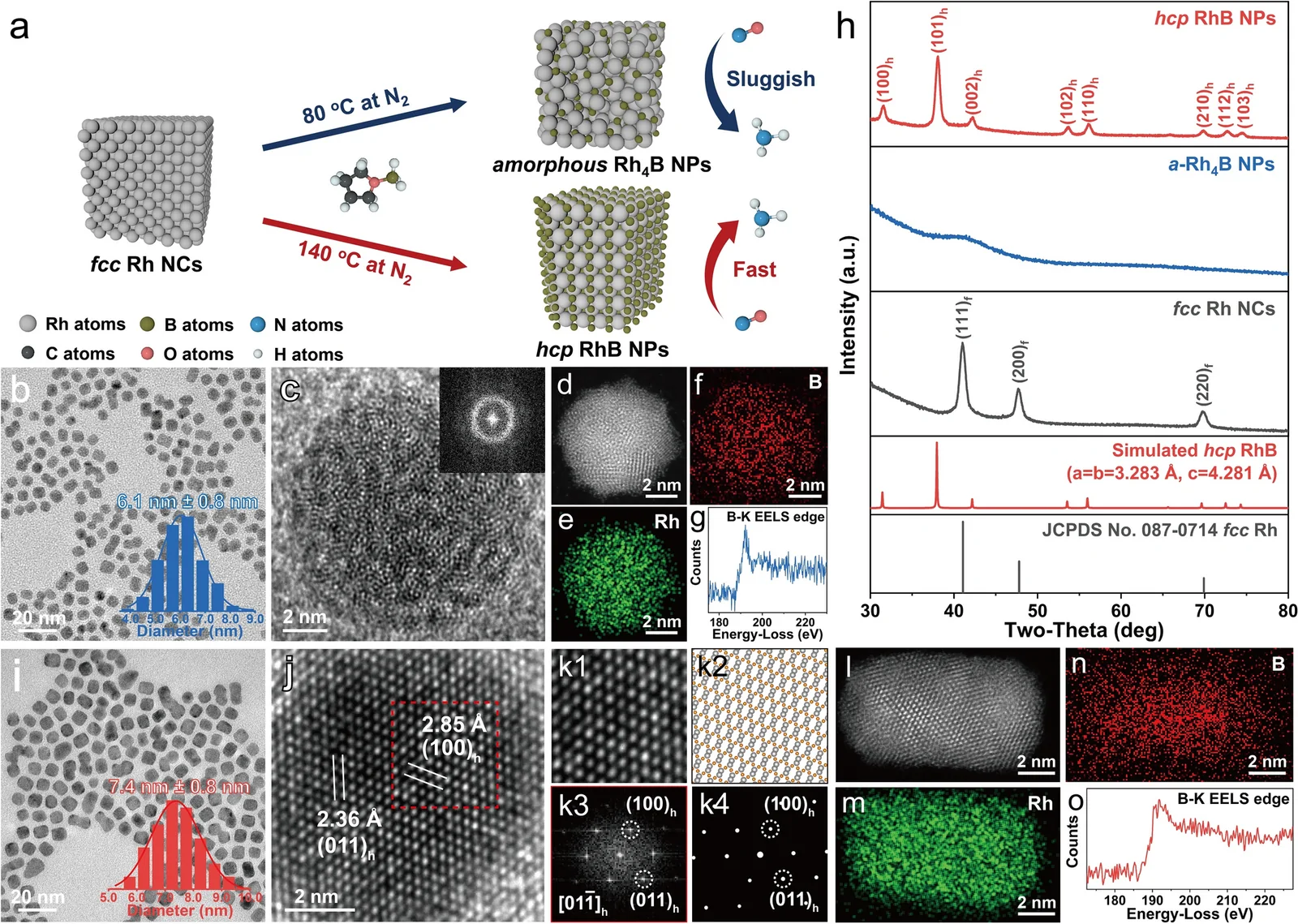
Synthesis and structural characterization of face-centered cubic (*fcc*) Rh nanocubes (Rh NCs), amorphous Rh_4_B nanoparticles (*a*-Rh_4_B NPs), and hexagonal close-packed (*hcp*) RhB NPs. **a** Schematic illustration of temperature-regulated synthesis of *a*-Rh_4_B NPs and *hcp* RhB NPs using Rh NCs as seeds. **b** Low-magnification transmission electron microscopy (TEM) image of *a*-Rh_4_B NPs. **c** High-resolution TEM (HRTEM) image of a typical *a*-Rh_4_B NP and the corresponding fast Fourier transform (FFT) pattern (inset). **d-g** Aberration-corrected high-angle annular dark-field scanning TEM (HAADF-STEM) image (**d)**, the corresponding STEM-energy-dispersive X-ray spectroscopy (EDS) element mapping of Rh (**e)**, the corresponding electron energy loss spectroscopy (EELS) mapping of B (peak area at 181.4–205.9 eV) (**f)**, and B-K EELS edge spectrum (**g)** of a typical *a*-Rh_4_B NP. **h** X-ray diffraction patterns of Rh NCs, *a*-Rh_4_B NPs, and *hcp* RhB NPs. **i** TEM image of *hcp* RhB NPs. **j** HRTEM image of a typical *hcp* RhB NP. **k1-4**) Enlarged atomic resolution image (**k1**) taken from the selected red dashed region in (j) and the corresponding simulated atomic model (**k2**) of *hcp* RhB oriented along the [01 $\stackrel{\mathrm{-}}{1}$]_h_ zone axis with corresponding FFT patterns (**k3**) and simulated electron diffraction pattern (**k4**), respectively. Gray and orange dots represent Rh and B atoms, respectively. **l-o** HAADF-STEM image (**l**), the corresponding STEM-EDS element mapping of Rh (**m**), the corresponding EELS mapping of B (**n**), and the B-K EELS edge spectrum (**o**) of a typical *hcp* RhB NP

The phase-controlled synthesis of boronized Rh nanomaterials is illustrated in Fig. 1a. Briefly, *fcc* Rh NCs with an average edge length of 5.2 ± 0.7 nm were first synthesized by a previously reported method with minor modifications (Fig. S1, see Supplementary Methods for experimental details) [40, 45]. A subsequent B-insertion process was carried out using the Rh NCs as seeds and borane–tetrahydrofuran (BH_3_-THF) as B source. At relatively low temperature of 80 °C, *a*-Rh_4_B NPs were obtained, while at relatively high temperature of 140 °C, *hcp* RhB NPs were prepared.

As revealed in Fig. 1b, the obtained *a*-Rh_4_B NPs have relative uniform size of 6.1 ± 0.8 nm. The amorphous structure of *a*-Rh_4_B NPs was confirmed by the random atomic arrangement in the high-resolution transmission electron microscopy (HRTEM) image (Fig. 1c) of a typical *a*-Rh_4_B NP, the diffuse rings in its corresponding fast Fourier transform (FFT) pattern (inset of Fig. 1c) as well as in the selected-area electron diffraction (SAED) pattern of *a*-Rh_4_B NPs in a larger scale (Fig. S2a). The local elemental analysis of one typical *a*-Rh_4_B NP (Fig. 1d) was further conducted. Figure 1e-g reveals the energy-dispersive X-ray spectroscopy (EDS) element mapping of Rh, electron energy loss spectroscopy (EELS) mapping of B-K edge, and the corresponding B-K EELS edge spectrum, respectively, suggesting the homogeneous distribution of Rh and B elements without notable composition segregation [46, 47]. The atomic ratio of Rh to B is determined to be 81.3:18.7 by inductively coupled plasma optical emission spectrometry (ICP-OES) (Fig. S2b), close to 4:1. Moreover, in the X-ray diffraction (XRD) pattern of *a*-Rh_4_B NPs (Fig. 1h), no obvious diffraction peaks are observed. The *hcp* RhB NPs obtained at elevated reaction temperature have a mean size of 7.4 ± 0.8 nm (Fig. 1i). The characteristic peaks in XRD pattern of *hcp* RhB NPs (Fig. 1h) and the diffraction rings in SAED pattern (Fig. S3a) fit well with the diffraction patterns of *hcp* RhB based on the lattice parameters of *a* = *b* = 3.283 Å and *c* = 4.281 Å (Fig. S4). HRTEM image of a representative *hcp* RhB NP (Fig. 1j) reveals interplanar spacings of 2.85 Å and 2.36 Å, which are assigned to (100)_h_ and (011)_h_ planes of *hcp* RhB, respectively. The periodic atomic arrangement of Rh can be clearly observed in the enlarged atomic resolution image, which is consistent with simulated crystal structure model (Fig. 1k2) viewed from the [01$\stackrel{\mathrm{-}}{1}$]_h_ zone axis. The corresponding FFT pattern (Fig. 1k3) matches well with the simulated electron diffraction pattern (Fig. 1k4) of *hcp* RhB along the [01$\stackrel{\mathrm{-}}{1}$]_h_ zone axis, confirming the single crystal nature. The typical atomic stacking mode of *hcp* phase, “ABAB” along the [001]_h_ close-packed direction, was also observed from the HRTEM image of another *hcp* RhB NP with well-assigned corresponding FFT pattern (Fig. S3b, c). The local elemental analysis of one typical *hcp* RhB NP (Fig. 1l) is illustrated in Fig. 1m–o, revealing the EDS element mapping of Rh, EELS mapping of B-K edge, and the corresponding B-K EELS edge spectrum, respectively. Line-scan elemental analysis (Fig. S5) also confirms the homogeneous B distribution inside the *hcp* RhB NP. These results suggest the uniform distribution of Rh and B elements. The atomic ratio of Rh to B is determined to be 54.1:45.9 by ICP-OES (Fig. S3d), close to 1:1. The facile and controlled B-insertion are believed to play a key role in the formation of *a*-Rh_4_B NPs and *hcp* RhB NPs. By using an alternative B source (dimethylamine borane), *a*-Rh_4_B NPs (Fig. S6a, b), and *hcp* RhB NPs (Fig. S6c, d) were also obtained at slightly higher temperature, indicating the versatility of our phase-regulation strategy. The distinct phases of *a*-Rh_4_B NPs and *hcp* RhB NPs are believed to be directly related to the B content [38, 48], which could be controlled by the thermodynamically influenced B-insertion process. Control experiments suggest that at reaction temperatures below 80 °C, the *fcc* phase gradually diminishes toward amorphous phase, while at reaction temperature above 100 °C, *hcp* phase starts to form (Fig. S7). With detailed HRTEM characterizations (Figs. 1c, j and S8) and compositional analyses (Figs. S2, S3, and S8) of samples obtained at different reaction temperatures (60–140 °C), a temperature-dependent, B-content-related phase evolution of Rh nanocrystals of “*fcc* Rh → *a*-Rh_4_B → *a*-RhₓB/*hcp* RhB heterophase → *hcp* RhB” is collectively demonstrated (Fig. S9). It is worth mentioning that size control of *hcp* RhB NPs was also realized by simply using larger Rh NCs as the template, from which larger *hcp* RhB NPs with the average edge length of 18.8 ± 1.0 nm were successfully obtained (Figs. S10–11), suggesting the universality of the synthetic method.

**Fig. 2:**
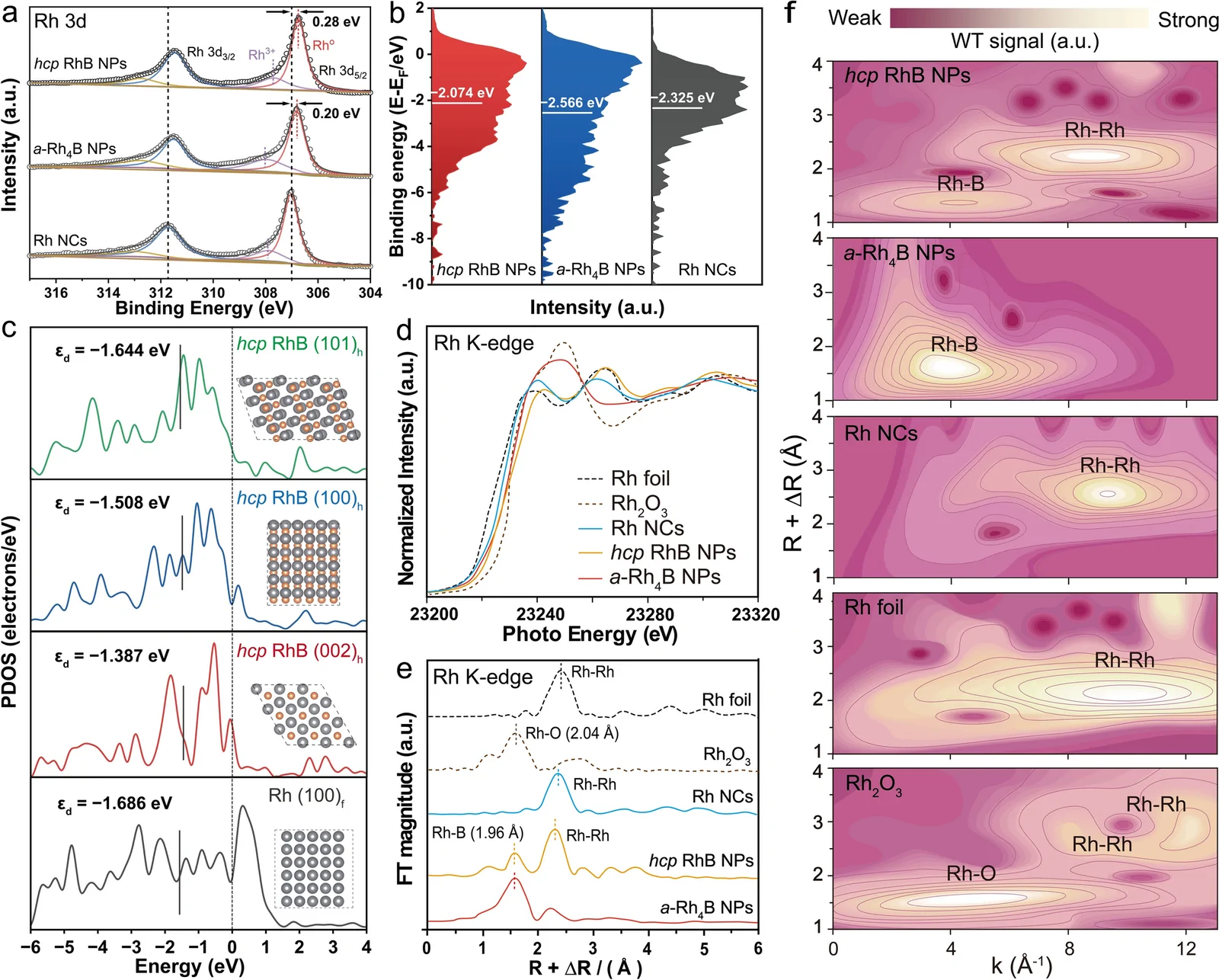
Chemical environment and electronic structure characterizations of Rh NCs, *a*-Rh_4_B NPs, and *hcp* RhB NPs. **a** High-resolution XPS spectra of Rh 3d of Rh NCs, *a*-Rh_4_B NPs, and *hcp* RhB NPs. **b** XPS valence band spectra of *hcp* RhB NPs, *a*-Rh_4_B NPs, and Rh NCs. **c** Projected density of states (PDOSs) of Rh over *fcc* Rh (100)_f_, *hcp* RhB (002)_h_, *hcp* RhB (100)_h_, and *hcp* RhB (101)_h_ surfaces. The E_F_ is set to zero (dashed line) and the location of d-band center (ɛ_d_) is marked by a solid line. The corresponding atomic structures of *fcc* Rh (100)_f_, *hcp* RhB (002)_h_, *hcp* RhB (100)_h_, and *hcp* RhB (101)_h_ surfaces are provided. Gray and brownish-yellow balls represent Rh and B atoms, respectively. **d, e** Normalized Rh K-edge X-ray absorption near-edge structure spectra and Fourier transform of k^2^-weighted X-ray absorption fine structure (EXAFS) spectra of Rh NCs, *a*-Rh_4_B NPs, and *hcp* RhB NPs in reference to Rh foil and Rh_2_O_3_. **f** Wavelet transforms for the k^2^-weighted Rh K-edge EXAFS spectra of *hcp* RhB NPs, *a*-Rh_4_B NPs, Rh NCs, Rh foil, and Rh_2_O_3_

Comprehensive investigations of the chemical environments and electronic structures of Rh NCs, *a*-Rh_4_B NPs, and *hcp* RhB NPs are of critical importance to understand the structural modification introduced by B-insertion in terms of electron density distribution, band structure, coordination environment, and phase stability. Figure 2a shows the X-ray photoelectron spectroscopy (XPS) analyses of the three samples. The dominant peaks located at about 307.0 and 311.8 eV in Rh NCs are attributed to metallic Rh^0^ 3*d*_5/2_ and Rh^0^ 3*d*_3/2_, respectively [49]. Compared with Rh NCs, the peak positions of Rh^0^ in *a*-Rh_4_B NPs shift negatively by 0.2 eV, which demonstrates the electron transfer from B to Rh in *a*-Rh_4_B NPs [50]. Interestingly, the positions of Rh^0^ in *hcp* RhB NPs shifted negatively by around 0.28 eV, also indicating the electron redistribution of Rh sites. Besides, the B XPS spectrum confirms that B^0^ is the major state of B element (Fig. S12). Furthermore, XPS valence spectroscopy was applied to study their electronic structures (Fig. 2b). The *d*-band center of *a*-Rh_4_B NPs (− 2.566 eV) downshifts compared with that of Rh NCs (− 2.325 eV). In contrast, the d-band center of *hcp* RhB NPs shifts upwards to − 2.074 eV. The upshifted d-band center in XPS valence could be beneficial for the adsorption and activation of key intermediates of NORR [29, 51]. To better understand the different 3d-band center (ɛ_d_) profiles of *hcp* RhB NPs and Rh NCs, DFT calculation was applied (see Supplementary Methods for calculation details) to visualize the projected density of states (PDOSs) of Rh 3d band over *fcc* Rh (100)_f_ and major *hcp* surfaces of RhB including (002)_h_, (100)_h_, and (101)_h_ (Fig. 2c). The ɛ_d_ of Rh on *hcp* RhB (002)_h_, *hcp* RhB (100)_h_, and *hcp* RhB (101)_h_ are − 1.387, − 1.508, and − 1.644 eV, respectively, all of which are closer to E_F_ (0 eV), compared to that of *fcc* Rh (100)_f_ (− 1.686 eV), coinciding with the d-band center upshifts of *hcp* RhB NPs in the XPS valence spectra. The more positive ɛ_d_ values of Rh sites on *hcp* RhB reflect more empty antibonding states, particularly at higher energy states, suggesting a stronger molecule adsorption capability of *hcp* RhB than that of *fcc* Rh [52]. In addition, the differential charge density analysis of RhB(002), RhB(100), and RhB(101) facets (Fig. S13) demonstrates B-induced electron accumulation on Rh sites, directly correlating with XPS and XAS results which illustrate the electron transfer from B to Rh, collectively confirming the electronic structure modification of Rh active sites.

Furthermore, X-ray absorption near-edge structure (XANES) and the extended X-ray absorption fine structure (EXAFS) spectroscopies were utilized to elucidate the electronic structures and local environments of Rh in Rh NCs, *a*-Rh_4_B NPs, and *hcp* RhB NPs. As shown in Fig. 2d, the white line positions of *a*-Rh_4_B NPs and *hcp* RhB NPs lie between those of Rh_2_O_3_ and Rh foil, suggesting the co-existence of metallic and valence states of Rh [53]. Besides, the reduced intensity of white line in *hcp* RhB NPs compared with that in Rh NCs indicates an electron transfer from B to Rh [54], in agreement with the XPS results. Figure 2e shows the Fourier-transformed k^2^-weighted EXAFS spectra of the three samples in reference to Rh foil and Rh_2_O_3_ in the R space. The peak located at about 2.72 Å is attributed to Rh-Rh scattering paths [55]. A summary of Rh K-edge EXAFS fitting results is listed in Fig. S14 and Table S1. The dominant metallic state of Rh in *hcp* RhB NPs and Rh NCs is confirmed by the predominant Rh-Rh coordination and the absence of the Rh-O scattering path [56], which is consistent with XPS and XANES results. Importantly, RhB scattering path is identified at peak position of 1.96 Å in the EXAFS spectra of *hcp* RhB NPs and *a*-Rh_4_B NPs, which is ~ 0.1 Å shorter than that of Rh-O path. Note that the RhB coordination is strongly predominant in *a*-Rh_4_B NPs, indicating that Rh atoms are largely isolated by B atoms. Furthermore, the coordination environment profiles were validated by visualizing the wavelet transforms (WT) of Rh K-edge EXAFS spectra of different samples. As revealed in Fig. 2f, the WT demonstrated the co-existence of Rh-Rh and RhB bonds in *hcp* RhB NPs, while only obvious RhB bond is observed in *a*-Rh_4_B NPs. The center position of the Rh-Rh bond in *hcp* RhB NPs shifts to smaller k direction compared with Rh NCs and Rh foil, indicating reduced Rh-Rh coordination of second-coordination shells of Rh atoms in *hcp* RhB NPs due to the incorporation of light species of B atoms [57, 58]. Moreover, the maximum intensity profile of the Rh-Rh bond in *hcp* RhB NPs shows a narrower distribution than those in Rh NCs and Rh foil in R direction, also indicating reduced Rh-Rh coordination of second-coordination shells [57, 59]. Results from Fig. 2 indicate that the coordination environment of *hcp* RhB NPs has been modified, distinctly different from metallic Rh or *a*-Rh_4_B NPs, due to the introduction of B atoms and the formation of the *hcp* phase [60].

### NORR Performance Evaluation

**Fig. 3:**
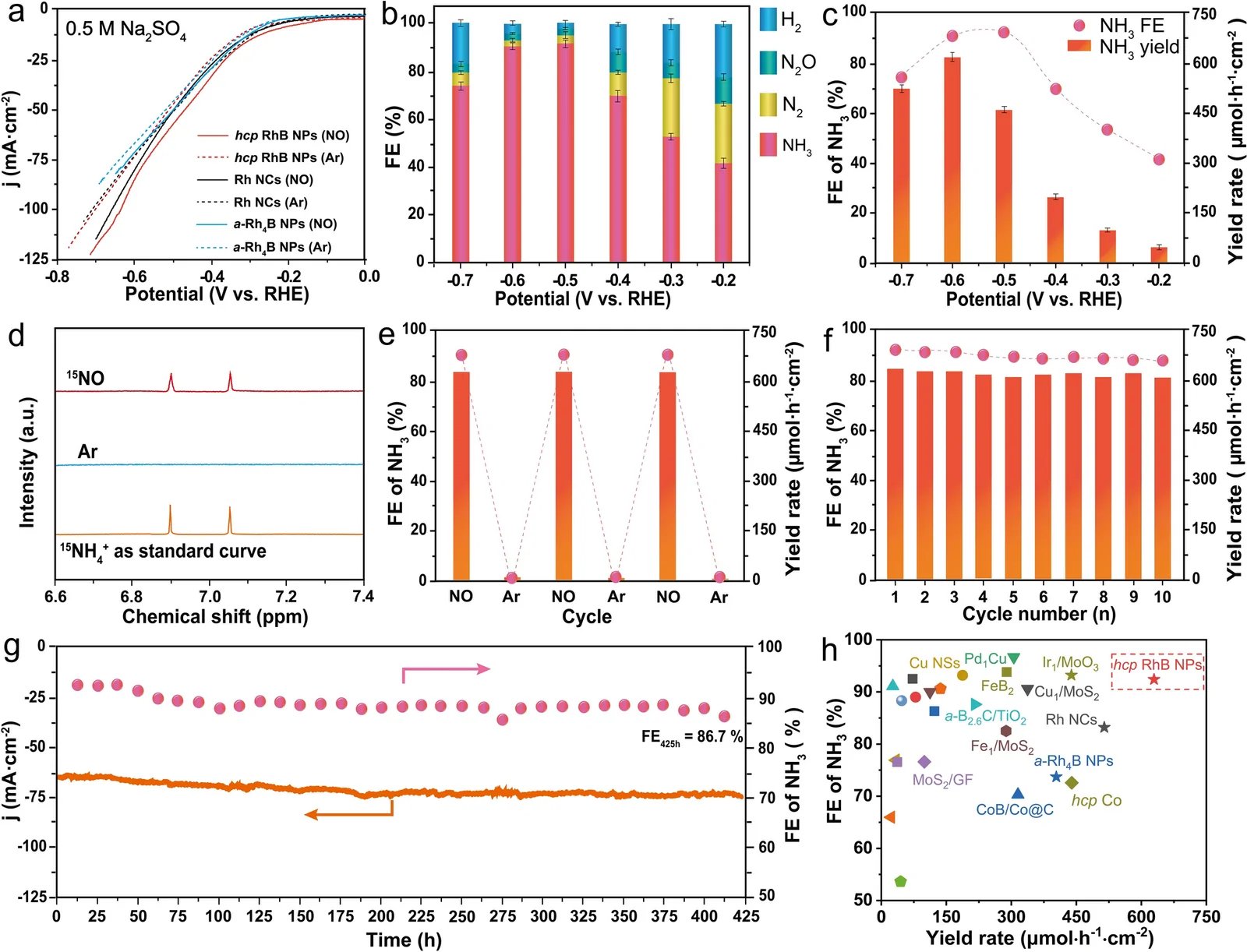
Electrocatalytic nitric oxide reduction reaction (NORR) performances of Rh NCs, *a*-Rh_4_B NPs, and *hcp* RhB NPs. **a** Linear sweep voltammetry curves of Rh NCs, *a*-Rh_4_B NPs, and *hcp* RhB NPs in Ar-saturated (dashed lines) and NO-saturated (solid Lines) 0.5 M Na_2_SO_4_ electrolyte. **b** Faradaic efficiencies (FEs) of all possible products including NH_3_, H_2_, N_2_O and N_2_ for *hcp* RhB NPs at each given potential. **c** NH_3_ yield rate and FE of NH_3_ (FE_NH3_) of *hcp* RhB NPs at different potentials. **d** ^1^H nuclear magnetic resonance spectra of the ^15^N isotope-labeling experiment over *hcp* RhB NPs. The ^1^^5^N isotope-labeling experiment is necessary to verify that the obtained NH_3_ originates from the feeding NO rather than contamination. **e** Alternating electrolysis test of *hcp* RhB NPs at − 0.6 V versus reversible hydrogen electrode (RHE). **f** FE_NH3_ and NH_3_ yield rate in 10 consecutive cycles over *hcp* RhB NPs for NORR at − 0.6 V vs. RHE. **g** Long-term stability test for 425 h over the *hcp* RhB NPs at − 0.5 V versus RHE. **h** Comparison of FE_NH3_ and NH_3_ yield rate between catalysts in our work and selected reported nanocatalysts performed in H-type electrochemical systems (details of the comparison are shown in Table S2)

The electrocatalytic NORR performances of obtained catalysts were evaluated in 0.5 M Na_2_SO_4_ aqueous solution using a gas-tight H-shape cell under ambient conditions (see Supplementary Methods for experimental details). In brief, the catalysts were loaded on Vulcan XC-72 carbon (15 wt%, Fig. S15) and dropped on carbon paper at 1.25 mg cm^−2^, which were used as the working electrodes. All potentials were converted and referred to RHE in this work. Before each electrochemical test, high-purity Ar gas was purged into the electrolyte for 30 min to eliminate O_2_. Figure 3a shows the linear sweep voltammetry (LSV) curves, in which the current densities of Rh NCs, *a*-Rh_4_B NPs, and *hcp* RhB NPs under NO/Ar (20 vol% NO) atmosphere were enhanced compared to those under saturated Ar (no NO gas), suggesting that electrocatalytic NORR occurred on all Rh samples in the presence of NO. Compared with Rh NCs and *a*-Rh_4_B NPs, *hcp* RhB NPs exhibit the most obvious enhancement of current density, suggesting the best NORR activity. Subsequently, the possible products, including NH_3_ (Fig. S16), N_2_H_4_ (Fig. S17), H_2_, N_2_O, and N_2_, at different applied potentials (Figs. S18-S20) were quantified and the corresponding Faradaic efficiencies (FEs) and NH_3_ yield rates were calculated. Surprisingly, *hcp* RhB NPs exhibit the maximum FE for NH_3_ (FE_NH3_) of 92.1% ± 1.2% at − 0.5 V (Fig. 3b**)**, much higher than those of Rh NCs (Fig. S21a, 83.2% ± 1.7% at − 0.6 V) and *a*-Rh_4_B NPs (Fig. S22a, 73.2% ± 2.1% at − 0.5 V). Moreover, the maximum NH_3_ yield rate of *hcp* RhB NPs at − 0.6 V is 629.5 ± 11.0 µmol h^−1^ cm^−2^ (Fig. 3c), superior to those of Rh NCs (Fig. S21b, 514.7 ± 12.0 µmol h^−1^ cm^−2^ at − 0.6 V) and *a*-Rh_4_B NPs (Fig. S22b, 403.8 ± 9.0 µmol h^−1^ cm^−2^ at − 0.6 V). The high FE_NH3_ and NH_3_ yield rate demonstrate the excellent selectivity and activity of *hcp* RhB NPs for electrocatalytic conversion of NO to NH_3_. These superior catalytic behaviors are believed to be irrelevant to size, as the as-prepared Rh NCs and *a*-Rh_4_B NPs are of similar sizes. For reference, the larger *hcp* RhB NPs exhibit a maximum FE_NH3_ of 71.2% ± 1.1% (Fig. S23a) and NH_3_ yield rate of 457.3 ± 13.0 µmol h^−1^ cm^−2^ at − 0.6 V (Fig. S23b), possibly due to lower surface-to-volume ratio. To verify that the produced ammonium all originated from the NO in the electrolyte, chronoamperometry measurements were conducted separately using Ar or ^15^NO as the feeding gas at − 0.6 V for 6.0 h. In Fig. 3d, the ^1^H nuclear magnetic resonance (NMR) (600 MHz) spectra only show typical double peaks of ^15^NH_4_^+^ when employing ^15^NO as the N source, confirming the origin of N source. Besides, alternating electrolysis on *hcp* RhB NPs was performed at − 0.6 V (Fig. 3e), showing consistently high FE_NH3_ and NH_3_ yield after repeatedly switching the electrolyte between Ar-saturated and NO-saturated ones. The well-maintained selectivity and activity for NH_3_ production on *hcp* RhB NPs demonstrate their robust electrocatalytic performance and excellent adaptability to the disturbance of electrolyte environment. An additional alternating electrolysis experiment by switching between NO-saturated and air-saturated electrolytes (Fig. S24) further confirms that the measured NH_3_ yield originates exclusively from NO reduction. Furthermore, in Fig. 3f, the NH_3_ yield rate and FE_NH3_ of *hcp* RhB NPs show no obvious decay after 10 consecutive cycles, confirming its excellent cycling stability. The long-term electrocatalytic stability of *hcp* RhB NPs was also evaluated at − 0.5 V (Fig. 3g). After the 425-h test, only small drops of FE_NH3_ and NH_3_ yield rate were found, indicating the great catalytic stability of *hcp* RhB NPs and therefore their potential for long-term industrial applications. Moreover, XRD pattern (Fig. S25), HAADF-STEM (Fig. S26a), HRTEM (Fig. S26b, c), ICP-OES (Fig. S26d), and XPS (Fig. S27) results of *hcp* RhB NPs after the durability tests show that the crystal structure, morphology, particle size, composition, and electronic structure can be well maintained. The consistent crystal lattice and neglectable change of Rh:B atomic ratio coupled with the negligible B^0^ peak shift demonstrates that B atoms remain intact in the Rh lattice and were not leached out during the electrocatalytic process. Notably, the excellent electrocatalytic NORR performances of our *hcp* RhB NPs put them among the best reported nanocatalysts tested in H-type electrochemical systems (Fig. 3h, Table S2).

### Mechanistic Study

**Fig. 4:**
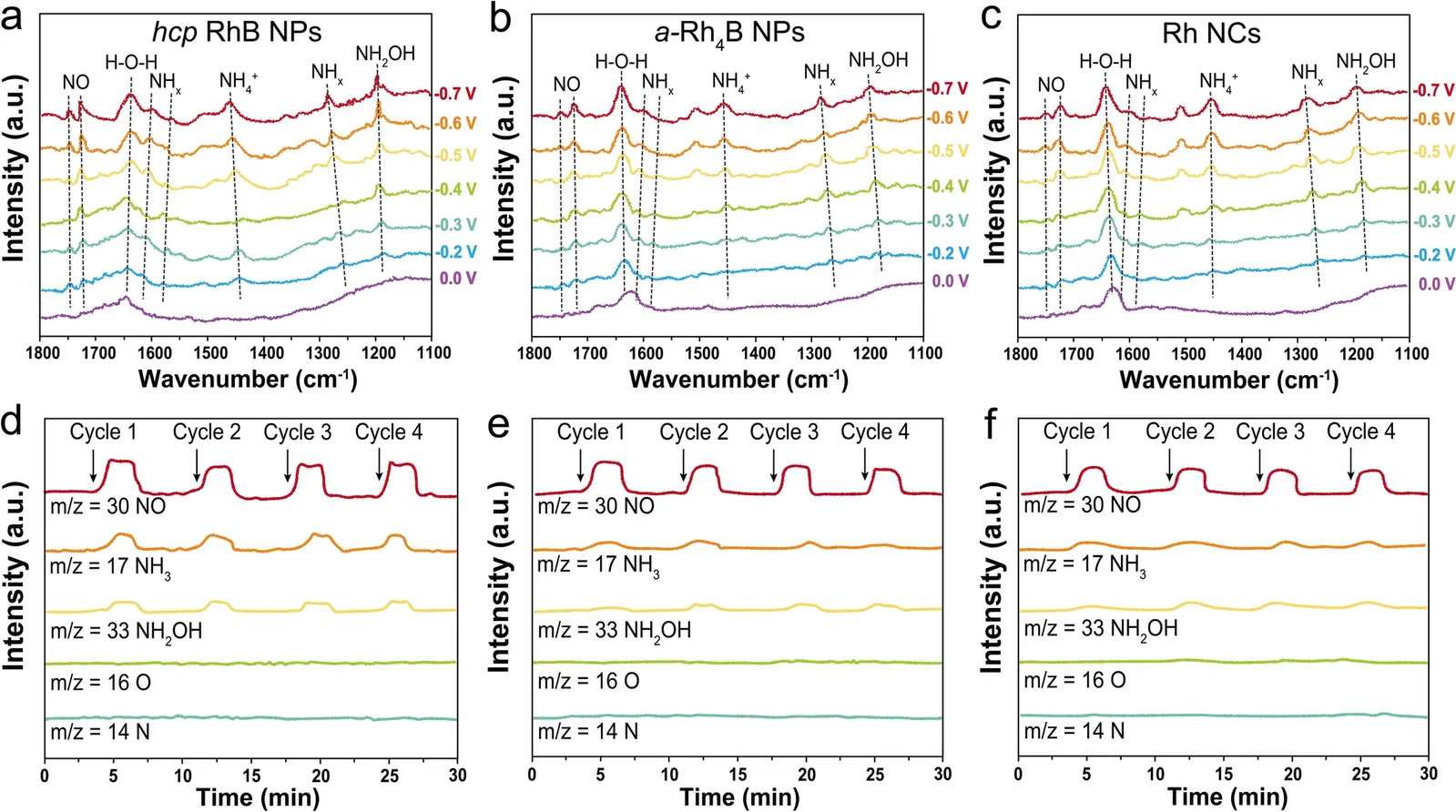
In situ spectroscopy analyses of electrocatalytic NORR intermediates of Rh NCs, *a*-Rh_4_B NPs and *hcp* RhB NPs. **a**-**c** Potential-dependent in situ attenuated total reflection infrared spectra of **a** *hcp* RhB NPs, **b** *a*-Rh_4_B NPs, and **c** Rh NCs for NORR in the range from 0 to − 0.7 V versus RHE. **d-f** In situ differential electrochemical mass spectrometry of **d** *hcp* RhB NPs, **e** *a*-Rh_4_B NPs, and **f** Rh NCs for NORR during four consecutive cycles at − 0.5 V versus RHE

To gain deeper understanding of the catalytic mechanisms over Rh NCs, *a*-Rh_4_B NPs, and *hcp* RhB NPs in electrocatalytic NORR, a series of in situ analyses were performed. First, in situ attenuated total reflection infrared (ATR-IR) spectroscopy was employed to identify the adsorbed intermediates on different catalyst surfaces. NO adsorption is the first and pivotal step in the electrocatalytic NORR, critically affecting the reaction kinetics of the subsequent steps. As revealed in Figs. 4a–c and S28, NO molecules are adsorbed on the surface of catalysts with both vertical mode at about 1750 cm^−1^ and bent mode at about 1720 cm^−1^. The peaks of NO in bent mode enhanced significantly as the applied potential increased, suggesting that NO molecule is effectively activated at more negative potentials [19, 61]. As shown in Figs. 4a and S28a, the peaks of NO in bent mode for *hcp* RhB NPs appear at relatively lower potential compared with those for *a*-Rh_4_B NPs (Figs. 4b and S28b) and Rh NCs (Figs. 4c and S28c), indicating that NO molecule is activated at an earlier stage on *hcp* RhB NPs. Meanwhile, the peak intensities of bent-mode NO on *hcp* RhB NPs are much stronger than those on *a*-Rh_4_B NPs and Rh NCs. These results demonstrate *hcp* RhB NPs exhibit superior NO adsorption and activation capability than *a*-Rh_4_B NPs and Rh NCs [15]. Then, the peaks at 1644 cm^−1^ are assigned to water, which is believed to provide protons for the hydrogenation of NO [19, 62, 63]. Furthermore, *NH_2_OH is another critical intermediate that significantly influences the NORR dynamics and reaction pathway [26, 64, 65]. The peak at ~ 1190 cm^−1^, ascribed to the adsorption of *NH_2_OH species, appears as early as initial potential applied and the intensity is enhanced with increasing potential. Further reaction of *NH_2_OH intermediates would produce the early species of NH_3_ (*NH_x_). The gradually increased peaks at 1610, 1565, and 1280 cm^−1^ can be attributed to the *NH_x_ species at bent mode, while the band at 1450 cm^−1^ implies the accumulation of numerous NH_4_^+^ products on the surface of catalysts [15, 66, 67]. Obviously, the peak intensities of *NH_x_ species and NH_4_^+^ products on *hcp* RhB NPs (Figs. 4a and S28a) are much stronger than those on *a*-Rh_4_B NPs (Figs. 4b and S28b) and Rh NCs (Figs. 4c and S28c), indicating highly efficient and active reaction dynamics of *hcp* RhB NPs for direct NO-to-NH_3_ conversion. In addition, key intermediates and products during electrocatalytic NORR process are detected using in situ differential electrochemical mass spectrometry (DEMS) (Fig. 4d-f). During four continuous cycles at − 0.5 V, the detected m/z signals of 14, 16, 17, 30, and 33 correspond to N, O, NH_3_, NO, and NH_2_OH, respectively. Much higher peak intensities of NO, NH_3_ and NH_2_OH were observed on *hcp* RhB NPs (Fig. 4d) compared to those on *a*-Rh_4_B NPs (Fig. 4e) and Rh NCs (Fig. 4f), suggesting stronger NO adsorption capability and accelerated reaction kinetics of NO-to-NH_3_ conversion [68]. Based on the in situ ATR-IR and DEMS observations, the NO reduction process over our catalysts is speculated to follow a series of proton-coupled electron transfer (PCET) steps and undergo two possible pathways. One pathway follows “NO → *NO → *NOH → *NHOH → *NH_2_OH → *NH_2_ → *NH_3_” (denoted as “*NO → *NOH” route, illustrated by schemes in Fig. S29) [26], and another follows “NO → *NO → *HNO → *NHOH → *NH_2_OH → *NH_2_ → *NH_3_” (denoted as “*NO → *HNO” route, illustrated by schemes in Fig. S30) [63, 69]. The main difference between these two pathways is whether proton bonds with N or O atom in the *NO intermediate in the first hydrogenation step.

**Fig. 5:**
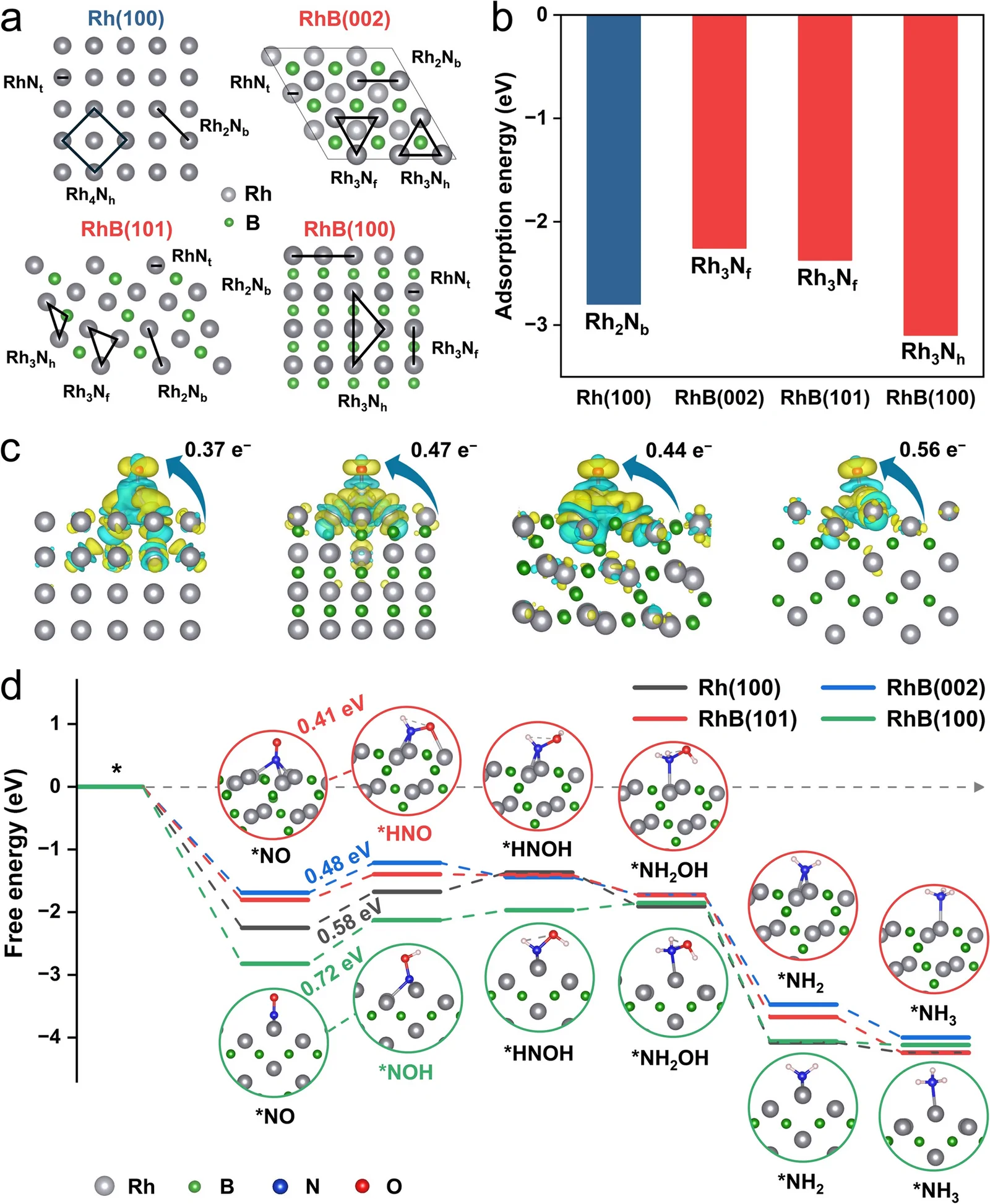
Theoretical simulations of NO adsorption, NO activation, and NORR pathways. **a** Possible NO adsorption configurations on Rh(100), RhB(002), RhB(101), and RhB(100) facets. Gray and green balls represent Rh and B atoms, respectively. The subscript letter, t, b, h, or f represents the relative position of N atoms to the Rh atoms. In brief, RhN_t_ means NO is adsorbed right on top of a Rh atom. Rh_2_N_b_ means NO is adsorbed at the center of a bridge site between two adjacent Rh atoms. Rh_4_N_h_ means NO is adsorbed at the Rh fourfold coordinated site. Rh_3_N_h_ means NO is adsorbed at the Rh threefold coordinated site in a hexagonal close-packed configuration. Rh_3_N_f_ means NO is adsorbed at Rh threefold coordinated site in a face-centered cubic configuration. **b** Calculated NO adsorption energy over the most favorable NO configurations on Rh(100), RhB(002), RhB(101), and RhB(100) facets. **c** Calculated Charge of Densities and Bader Charge profiles in the NO adsorption process over Rh(100), RhB(002), RhB(101), and RhB(100) facets. The colored shapes on the Rh lattice visualize the charge density differences at the interface between the adsorbed NO and surfaces (isovalue = 0.002 a.u.). **d** Reaction Gibbs free energy diagram of NORR intermediate steps of Rh(100), RhB(002), RhB(101), and RhB(100) facets. The optimized adsorption configurations on RhB(101) (highlighted in red circles) and RhB(100) (highlighted in green circles), representing two NORR pathways, are illustrated (the blue, red and light pink balls represent N, O and H atoms, respectively)

To gain insight into the origin of the outstanding NORR performance over *hcp* RhB NPs, DFT calculations toward the whole NORR process were employed. As NO adsorption is a critical step in the reaction pathway [21, 26, 64], the NO adsorption profiles were firstly investigated on the mostly commonly exposed *hcp* RhB facets, namely RhB(002), RhB(101), and RhB(100) facets, as well as a reference *fcc* Rh facet of Rh(100). Four common configurations of adsorption sites on RhB surfaces, including RhN_t_, Rh_2_N_b_, Rh_3_N_f_, and Rh_3_N_h_ (Fig. 5a), and three possible configurations of adsorption sites on Rh(100) surface, including RhN_t_, Rh_2_N_b_, and Rh_4_N_h_ are first investigated (Fig. S31) [15]. After evaluating the corresponding NO adsorption energies of different configurations on each facet (Table S3), the most favorable NO adsorption configuration on each surface is summarized in Fig. 5b, which were selected as the dominant configurations for further computations. Then, Bader Charge calculations were carried out on RhB(002), RhB(101), RhB(100), and Rh(100) facets (Table S4), showing significant charge transfer from these surfaces to the adsorbed NO molecule with corresponding electron gain values of 0.47, 0.44, 0.56, and 0.37 e^−^, respectively. In addition, PDOS analyses of the Rh d-orbitals on *hcp* RhB surfaces after NO adsorption generally become more dispersed with slight expansion to the low-energy direction (Fig. S32) [70]. The Bader Charge and PDOS analyses collectively suggest a stronger NO activation capability of *hcp* RhB compared to *fcc* Rh. The side views of the charge transfer process are illustrated in Fig. 5c (top views are provided in Fig. S33). Combining the differential charge density analysis (Figs. 5c and S33) which verifies the significant charge exchange at the interface between the adsorbed NO and catalyst surface, the weakened N–O bond and simultaneously strengthened Rh-N bond over *hcp* RhB facets are demonstrated, suggesting more effective NO activation. Notably, the theoretically verified improvement in NO adsorption and activation on *hcp* RhB align well with our observations from in situ ATR-IR and DEMS (Fig. 4a, d).

To further identify the reaction pathway and investigate the origin of activity and selectivity of NORR on Rh NCs and *hcp* RhB NPs, energy profiles of NORR through the two mentioned pathways including “*NO → *NOH” and “*NO → *HNO” were calculated. Firstly, the reaction Gibbs free energy (G) diagrams of “*NO → *HNO” and “*NO → *NOH” (Fig. S34) show that as ΔG for hydrogenation of *NO to *HNO is lower than that of *NO to *NOH (ΔG_*HNO_ < ΔG_*NOH_), manifesting that NORR follows the “*NO → *HNO” pathway on Rh(100), RhB(002), and RhB(101) facets (the corresponding structures in the case of RhB(101) are presented in Fig. 5d and the rest cases are presented in Fig. S35). Correspondingly, because ΔG_*HNO_ > ΔG_*NOH_ (Fig. S34), NORR undergoes the pathway of “*NO → *NOH” on RhB(100) surface (the corresponding structures of RhB(100) are presented in Fig. 5d). After confirming the reaction pathway, RDSs of the NORR over four surfaces are further revealed in Fig. 5d. The RDSs on Rh (100), RhB(002), and RhB(101) facets are found to be the hydrogenation of *NO to *HNO, with corresponding energy barriers of 0.58, 0.48, and 0.41 eV, respectively. The ΔG of the RDS on RhB(002) and RhB(101) facets are much lower than that of Rh(100) facet. The ΔG of the RDS on RhB(100) is identified to be the hydrogenation of *NO to *NOH, with a ΔG value of 0.72 eV, larger than those of Rh(100), RhB(002), and RhB(101) facets. In addition, the hydrogenation of *NO to *HNO on RhB(101) requires 0.07 eV less energy than on RhB(002). Furthermore, the subsequent hydrogenation reaction of *HNO to *HNOH on Rh(100) is endothermic, while all the successive hydrogenation steps on RhB(002), RhB(101), and RhB(100) facets are exothermic except the slightly endothermic *NOH to *HNOH and *HNOH to *NH_2_OH steps on RhB(100) (Fig. 5d and Table S5), suggesting that the reaction steps after RDS on RhB(002), RhB(101), and RhB(100) facets are more thermodynamically favorable compared to Rh(100). Moreover, the ΔG of *NO adsorption on all RhB surfaces and Rh(100) surface are found more negative than those of *H and *H_2_O adsorption, indicating an effective inhibition of the competing hydrogen evolution reaction (HER) (Fig. S36), which can be reflected by the significantly reduced FE for H_2_ production (Fig. 3b) [71]. The B-insertion-enhanced HER inhibition may also originate from the electronic structure modulation to Rh sites, as evidenced by the chemical environment analysis (Fig. 2a, d) and PDOS calculations (Fig. 2c), which could disrupt interfacial water dynamics, weaken H_2_O adsorption (Fig. S36), and ultimately reduce proton availability at the Rh site [72, 73]. The inhibited HER in *hcp* RhB NPs compared to Rh NCs is also experimentally observed (Fig. S37), consistent with the theoretical calculation. It is worth noting that *hcp* nanocrystals in an ideal Wulff morphology expose a dominant percentage of (101) facet [74]; therefore, the catalytic behaviors simulated on RhB (101) facet may also play a more dominant role in reflecting the electrocatalytic characteristics of *hcp* RhB NPs among the three calculated facets. Based on the above theoretical analysis, *hcp* RhB catalyst can not only lead to favorable NO activation, but also reduce the energy barriers of the RDS (hydrogenation of *NO) and its subsequent steps, thus remarkably boosting the catalytic performance of NORR.

### Zn–NO Battery Demonstration

**Fig. 6:**
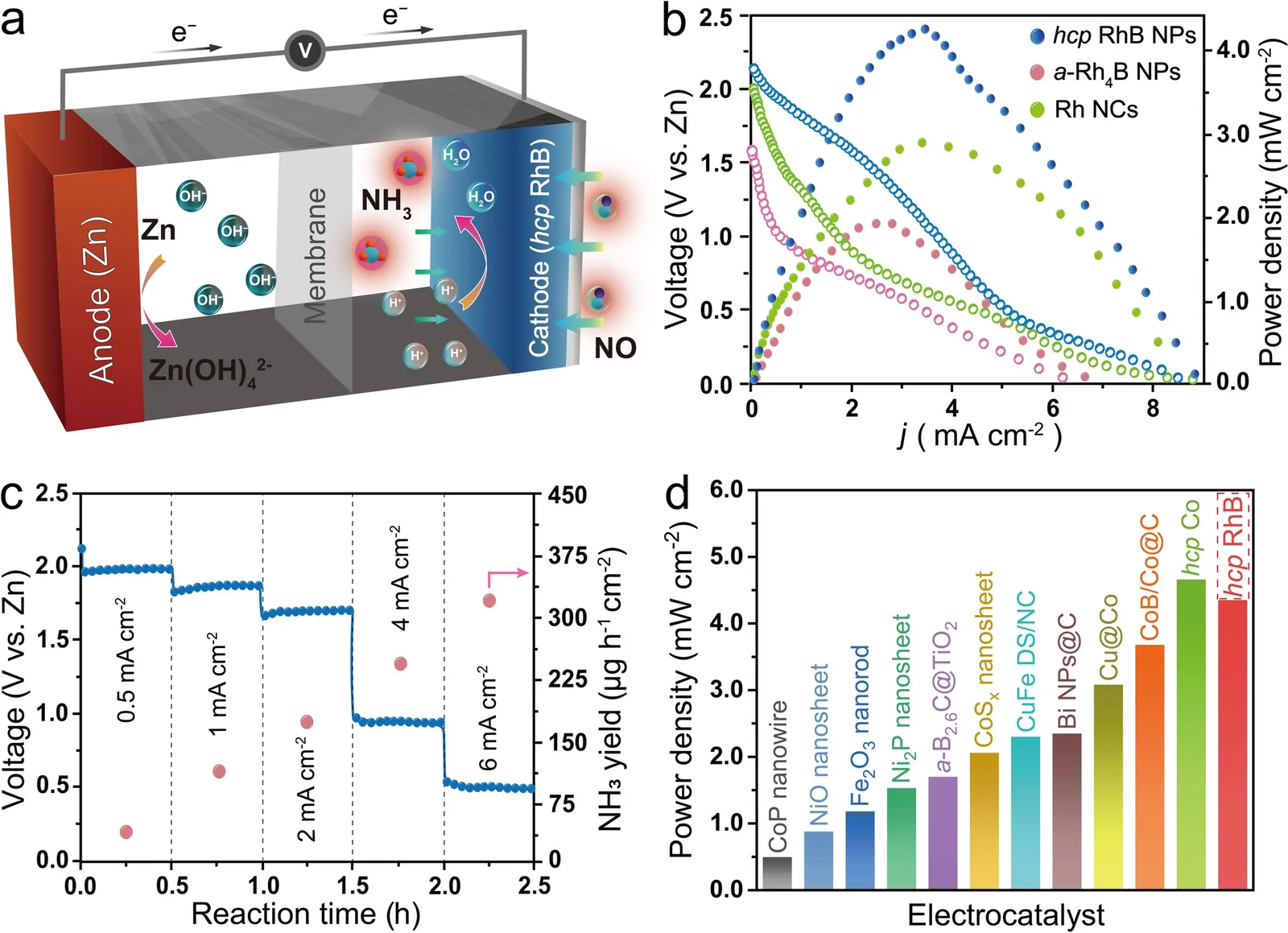
**a** Schematic illustration of the Zn−NO battery using *hcp* RhB NPs as the cathode. **b** Discharge polarization and power density plots of the Zn–NO batteries using Rh NCs, *a*-Rh_4_B NPs and *hcp* RhB NPs as the cathodes. **c** Discharge current density from 0.5 to 6 mA cm^−2^ and the corresponding NH_3_ yield rate of the Zn−NO battery at different voltages. **d** Comparison of peak power density of the *hcp* RhB NPs-based Zn−NO battery with selected representative Zn−NO battery systems without additional molecular additives reported to date (details of the comparison are shown in Table S6)

As a proof of concept, a Zn–NO battery is assembled, in which NORR takes place at the cathode and the performance of NORR catalyst greatly affects the overall operational efficiency and stability of the Zn–NO battery [75]. A series of Zn–NO battery systems are assembled using the Rh-based catalysts as the cathode, zinc plate as the anode, and 0.2 M Na_2_SO_4_ aqueous solution as the cathodic electrolyte (Fig. 6a). Using *hcp* RhB NPs as the cathode, the Zn–NO battery can deliver a peak power density of 4.33 mW cm^−2^, superior to those using Rh NCs and *a*-Rh_4_B NPs (2.91 and 1.94 mW cm^−2^, respectively) (Fig. 6b). An open-circuit voltage of 2.12 V was measured on the assembled battery (Fig. S38), demonstrating a strong thermodynamic driving force for NO conversion and energy generation. As shown in Fig. 6c, the Zn–NO battery with *hcp* RhB NPs exhibits remarkably stable and continuous discharge capability from 0.5 to 6.0 mA cm^−2^ with NH_3_ yield rate of 180.3 µg h^−1^ cm^−2^ at 2 mA cm^−2^. Moreover, post-test characterizations confirm the exceptional stability of *hcp* RhB NPs in terms of morphology (Fig. S39a), size (Fig. S39b), crystal phase (Fig. S39c), and composition (Fig. S39d). Notably, the *hcp* RhB NPs-based Zn–NO battery outperforms most Zn–NO batteries reported in literature to date (Fig. 6d and Table S6). This application suggests that the novel *hcp* RhB nanocatalysts possess impressive multi-functions in NO removal, NH_3_ synthesis, and electrical energy output.

## Conclusions

In conclusion, we successfully achieved the phase regulation of B-inserted Rh nanocrystals using a facile wet-chemical method. The reaction temperature is believed to play a key role in facilitating the formation of distinct phases, namely *a*-Rh_4_B and *hcp* RhB. Investigation of the chemical environments and electronic structures suggest that introduction of B leads to the electron redistribution of Rh atoms and upshift of d-band center in *hcp* RhB NPs, in contrast to *a*-Rh_4_B NPs. Impressively, the *hcp* RhB NPs achieve an excellent electrocatalytic selectivity and activity toward NORR with a FE_NH3_ up to 92.1% ± 1.2% with NH_3_ yield rate of 629.5 ± 11.0 µmol h^−1^ cm^−2^, outperforming the Rh NCs, *a*-Rh_4_B NPs, and most nanocatalysts reported to date. In situ spectroscopic measurements and DFT calculations suggest that Rh in *hcp* RhB NPs possess an upshift of d-band center, a stronger NO adsorption/activation capability, and a lower energy barrier for the RDS. Moreover, the assembled proof-of-concept Zn–NO battery suggests that the *hcp* RhB NPs can also serve as efficient cathode catalysts toward stable and high-power-density energy storage. Our work not only provides a new strategy for the lattice regulation of noble-metal-based nanostructures toward enhanced catalytic activity, selectivity, and stability, but also offers an attractive catalyst for efficient NORR and sustainable NH_3_ synthesis.

## Supplementary Information

Below is the link to the electronic supplementary material.

Supplementary file1 (DOCX 16918 kb)
