# Supplementary material for: Boron-Insertion-Induced Lattice Engineering of Rh Nanocrystals Toward Enhanced Electrocatalytic Conversion of Nitric Oxide to Ammonia
Source: Nanomicro Lett. 2025 Oct 5;18:74. doi: 10.1007/s40820-025-01919-6 (PMC12496378; doi:10.1007/s40820-025-01919-6)
Supplement: Supplementary file 1 — Supplementary file1 (DOCX 16918 kb) [file 40820_2025_1919_MOESM1_ESM.docx]

Supporting Information for

**Boron-Insertion-Induced Lattice Engineering of Rh Nanocrystals towards Enhanced Electrocatalytic Conversion of Nitric Oxide to Ammonia**

Peng Han^1,#^, Xiangou Xu^2,^ ^#^, Weiwei Chen^3^, Long Zheng^1^, Chen Ma^1^, Gang Wang^1^, Lei Xu^1^, Ping Gu^1^, Wenbin Wang^4^, Qiyuan He^4^, Zhiyuan Zeng^5^, Jinlan Wang^2^, Dong Su^3^, Chongyi Ling^2,^*, Zhengxiang Gu^6,^*, and Ye Chen^1,^*

^1^ Department of Chemistry, The Chinese University of Hong Kong, Hong Kong, P. R. China

^2^ Key Laboratory of Quantum Materials and Devices of Ministry of Education, School of Physics, Southeast University, Nanjing 211189, P. R. China

^3^ Beijing National Laboratory for Condensed Matter Physics, Institute of Physics, Chinese Academy of Sciences, Beijing 100190, P. R. China

^4^ Department of Materials Science and Engineering, City University of Hong Kong, Hong Kong, P. R. China

^5^ Department of Materials Science and Engineering, and State Key Laboratory of Marine Pollution, and Center of Super-Diamond and Advanced Films, City University of Hong Kong, Hong Kong, P. R. China

^6^ School of Chemistry and Materials Science, Nanjing Normal University, Nanjing 210023, P. R. China

^#^ Peng Han and Xiangou Xu contributed equally to this work.

*Corresponding authors. E-mail: [lingchy@seu.edu.cn](mailto:lingchy@seu.edu.cn) (Chongyi Ling); [zxgu16@fudan.edu.cn](mailto:zxgu16@fudan.edu.cn) (Zhengxiang Gu); [yechen@cuhk.edu.hk](mailto:yechen@cuhk.edu.hk) (Ye Chen)

# S1 Experimental Section

## S1.1 Chemicals and reagents

All chemicals were used as received without further purification. L-Ascorbic acid (AA, BioXtra, ≥99.0%, crystalline), potassium bromide (KBr, ≥99.0%), polyvinylpyrrolidone (PVP, powder, M_W_ ~55000), sodium hexachlororhodate(III) (Na_3_RhCl_6_, 97%), rhodium(III) chloride hydrate (RhCl_3_⸱xH_2_O, Rh 38-40%) tetrahydrofuran (THF, 99.9%, anhydrous), sodium sulfate (Na_2_SO_4_, ≥99.0%), sodium citrate tribasic dihydrate (≥99.0%), ammonium chloride (NH_4_Cl, ≥99.5%), sodium hydroxide (NaOH, ACS reagent, ≥97.0%), hydrazine hydrate (N_2_H_4_, reagent grade, 50-60%), potassium hydroxide (KOH, 99.99%), p-dimethylaminobenzaldehyde (C_9_H_11_NO, 99%), dimethylamine borane (DMAB, (CH_3_)_2_NH·BH_3_, 97%), and ethylene glycol (EG, 99.8%, anhydrous) were all purchased from Sigma-Aldrich. Borane–tetrahydrofuran (BH_3_-THF, 1 M in THF), sodium nitroferricyanide dihydrate (C_5_FeN_6_Na_2_O·2H_2_O), salicylic acid (≥99%), sodium hypochlorite (NaClO, available chlorine ≥30%), and carbon (C, Cabot Vulcan XC-72) were purchased from Macklin. Nafion 115 membrane was purchased from Aladdin. Nafion D-521 dispersion (5% w/w in water and 1-propanol) was purchased from Thermo Scientific Chemicals. Fumasep FBM-bipolar membrane was purchased from Fuel Cell Store. Ethanol (HPLC, 99.9%) and acetone (HPLC, 99.9%), and hydrochloric acid (HCl, AR, 37%) were purchased from RCI Labscan (Thailand). N_2_ gas (99.95%) and Ar (99.95%) were provided by Industrial from Linde HKO Limited (Hong Kong). All aqueous solutions are prepared using Milli-Q water with a resistivity of 18.2 MΩ·cm at room temperature.

## S1.2 Structural characterizations

The transmission electron microscopy (TEM) images are acquired on an FEI Tecnai Spirit 12 microscope operated at 120 kV. High-resolution TEM (HRTEM) images are carried out on an FEI Tecnai F20 microscope operated at 200 kV. The high-angle annular dark-field scanning transmission electron microscopy (HAADF-STEM) and energy-dispersive X-ray spectroscopy (EDS) elemental mapping images are performed on a JEOL JEM-NeoAEM-200F TEM performed at 200 kV. X-ray diffraction (XRD) patterns are measured by a Rigaku SmartLab diffractometer with Cu Kα radiation (λ=1.5406 Å). X-ray photoelectron spectroscopy (XPS) is conducted on PHI 5000 Versaprobe III system. A Bruker 600 M nuclear magnetic resonance (NMR) instrument with water suppression was used to record the ^1^H-NMR spectra. The X-ray absorption fine structures (XAFS) of Rh NCs, *a*-Rh_4_B NPs, *hcp* RhB NPs, Rh foil and Rh_2_O_3_ were measured at BL14W1 beamline in Shanghai Synchrotron Radiation Facility (Shanghai, China). The edge energy of the X-ray absorption near edge structure (XANES) spectra were determined from the maximum value in the first derivative of the leading edge of the XANES spectrum. The coordination parameters were obtained by fitting the R-space Fourier transformed data using the least square in Artemis. XAS data normalization and background subtraction were performed using Demeter 0.9.26 software package. The inductively coupled plasma optical emission spectrometry (ICP-OES) is taken on an ICPE 9820 model.

## S1.3 Quantification of products concentration

NH_4_^+^ quantification. The produced NH_3_ was quantitatively determined using the indophenol blue method [S1]. Typically, 5 ml of electrolyte was withdrawn from the reaction cell and diluted to 2 ml. Then, 2 ml of 1 M NaOH solution containing sodium citrate and salicylic acid (stored at 4 °C) together with 1 ml of freshly prepared 0.05 M NaClO were added. The mixed solution was shaken for a few seconds. Finally, 0.2 ml of a 1 wt.% sodium nitroferricyanide solution (stored at 4 °C) was added for the color reaction. After keeping at room temperature for 1 h, the resulting solution was measured using an ultraviolet–visible (UV–Vis) spectrophotometer. The absorbance at ~655 nm was used to determine the concentration of NH_3_. To quantify the amount of NH_3_, a calibration curve was built using a standard NH_4_Cl (≥99.5%) solution in 0.5 M Na_2_SO_4_. The fitting curve (y = 0.420x − 0.0018, R^2^ = 0.9998) showed a good linear relation of absorbance value with NH_3_ concentrations.

N_2_H_4_ quantification. The N_2_H_4_ was quantitatively determined by Watt and Chrisp method [S2]. A mixed solution of 1.97 g C_9_H_11_NO, 10 mL concentrated HCl, and 100 mL ethanol was used as a color reagent. The calibration curve was plotted as follow: (i) collecting the electrolyte from the cathode compartment or preparing a series of N_2_H_4_ solutions of known concentration as standards; (ii) adding 4 mL color reagent into above solutions separately and standing 20 min at room temperature; (iii) measuring the absorbance of the resulting solution at 460 nm in 10 mm glass cuvette by the Ultraviolet-visible (UV-Vis) spectrophotometer. The fitting curve shows a good linear relation of absorbance with N_2_H_4_ concentration (y = 0.425x + 0.055, R^2^ = 0.9998).

Gas chromatography (Agilent Technologies 7890B) and mass chromatography (Pfeiffer GSD 320 O2) were used to quantify gas products, including H_2_, N_2_, NO and N_2_O.

## S1.4 Calculation of Faradaic efficiency (FE) and the NH_3_ yield rate

The FE for NH_3_ electrosynthesis was defined as the amount of electric charge used for producing NH_3_ divided by the total charge passed through the electrodes during electrolysis. The FE was calculated according to the following equation:

FE = n × F × c × V / (M × Q) (S1)

The NH_3_ yield was calculated using the following equation:

NH_3_ yield = C_NH3_ × V / (17 × t × S) (S2)

where n is the number of electrons needed to produce one product molecule, F is the Faraday constant (96485 C mol^–1^), C is the measured mass concentration of the product, V is the volume of the cathodic reaction electrolyte (50 mL), M is the relative molecular mass of a specific product, Q is the quantity of applied charge/electricity, t is the duration for applying the potential (1 h), and S is the geometric area of the working electrode (1 cm^2^).

## S1.5 In-situ attenuated total reflection infrared (ATR-IR) spectroscopy measurements

The crystalline Ge substrates were used to increase the reflected signals. The catalyst-loaded gold film supported by crystalline silicon, platinum wire, and Ag/AgCl electrode served as the working electrode, counter electrode, and reference electrode, respectively. NO flow was constantly purged before and during the tests. LSV curves were performed in the range of 0.0 to –0.7 V with a scan rate of 1 mV s^-1^.

## S1.6 Zn-NO battery assembly and measurement

The electrocatalysts were employed as the cathode in a cathodic electrolyte (0.2 M Na_2_SO_4_). A polished Zn plate was employed in an anodic electrolyte (1.0 M KOH), and a bipolar membrane was used to separate the two electrolytes. During the battery discharge process, NORR occurs at the cathode side, while Zn converts to ZnO at the anode side. The electrochemical reaction on each electrode was described from the following equations:

Cathode: NO + 5H^+^ + 5e^–^ → NH_3_ + H_2_O (S3)

Anode: Zn + 2OH^–^ → ZnO + H_2_O + 2e^–^ (S4)

Overall: 5Zn + 2NO + 3H_2_O → 5ZnO + 2NH_3_ (S5)

The NH_3_ yield was quantified using a colorimetric method. The power density was calculated from the equation (P = U × *j*) using the polarization results, where U is the voltage and *j* the current density.

# S2 Supplementary Figures

**
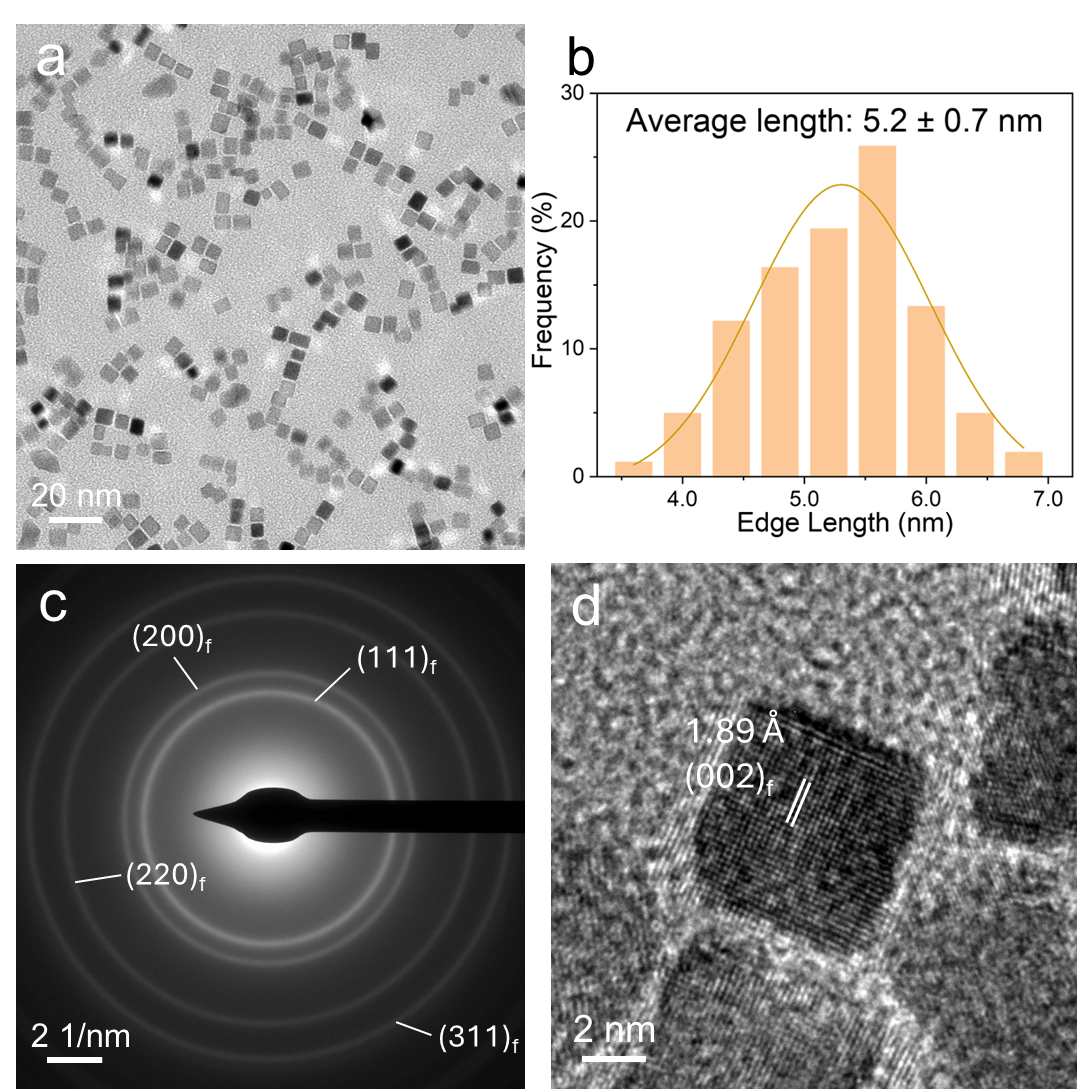
**

**Fig. S1** (**a**) Low-magnification TEM image with size distribution histogram, (**b**) Size distribution diagram, (**c**) Selected area electron diffraction (SAED) pattern, and (**d)** The HRTEM image of Rh NCs


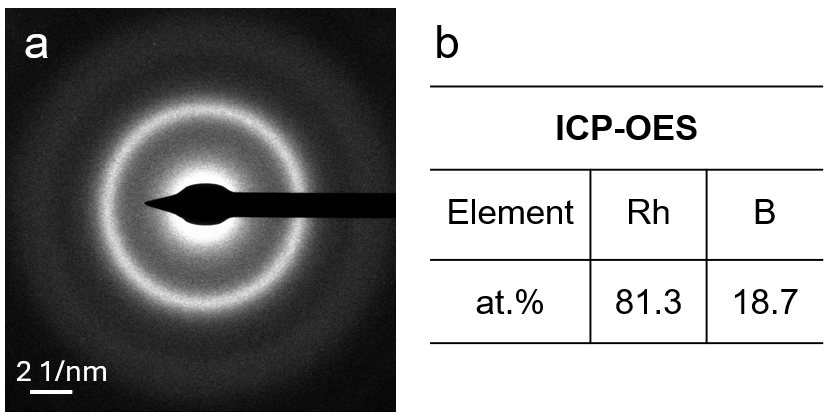


**Fig. S2** (**a**) SAED pattern of *a*-Rh_4_B NPs. (**b**) Elemental analysis result of *a*-Rh_4_B NPs from ICP-OES


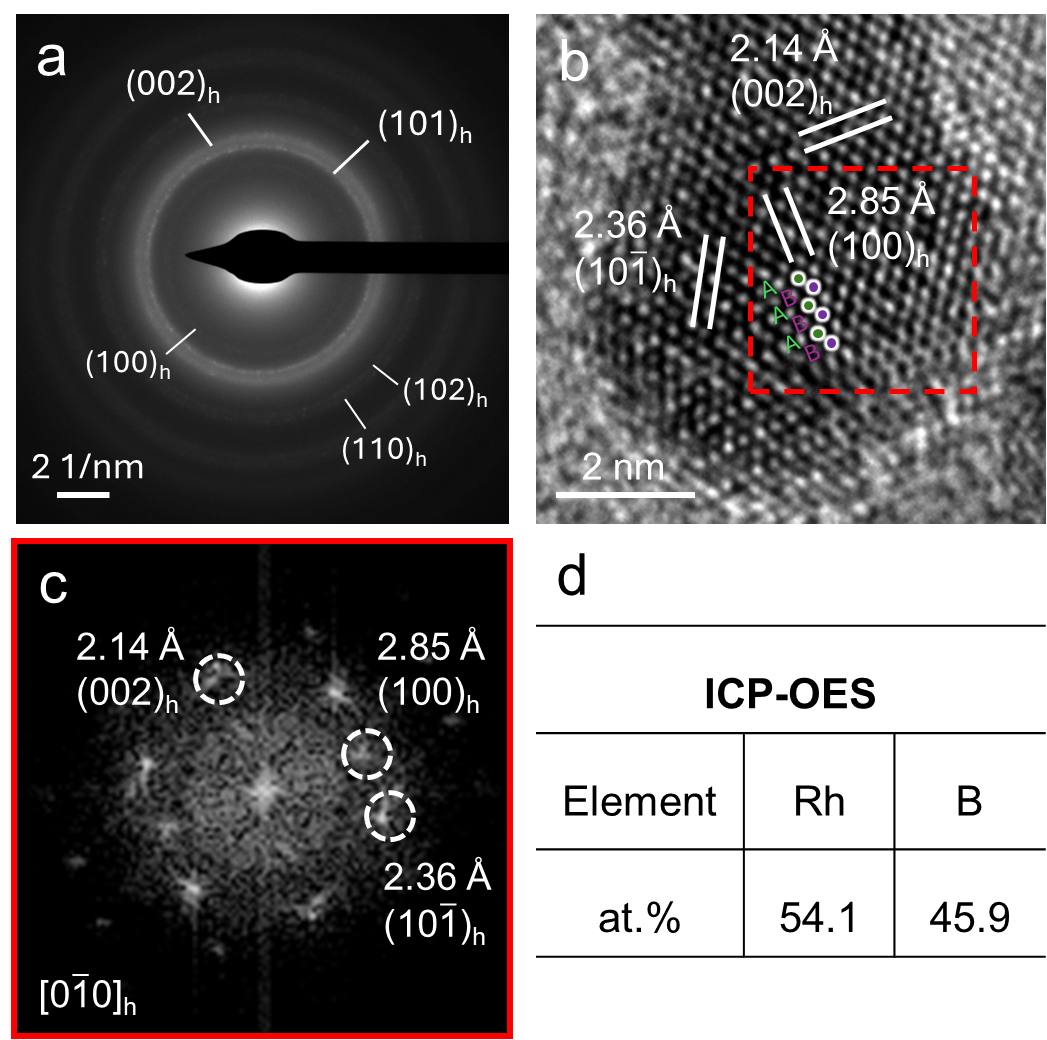


**Fig. S3** (**a**) SAED pattern, (**b**) HRTEM image (**c**) The corresponding fast Fourier transform (FFT) pattern of the selected square area marked in (b), and (**d**) ICP-OES result of *hcp* RhB NPs. The HRTEM image (Fig. S3b) of a representative *hcp* RhB NP show the typical atomic stacking mode of a *hcp* phase: i.e., “ABAB” along the [001]_h_ close-packed direction


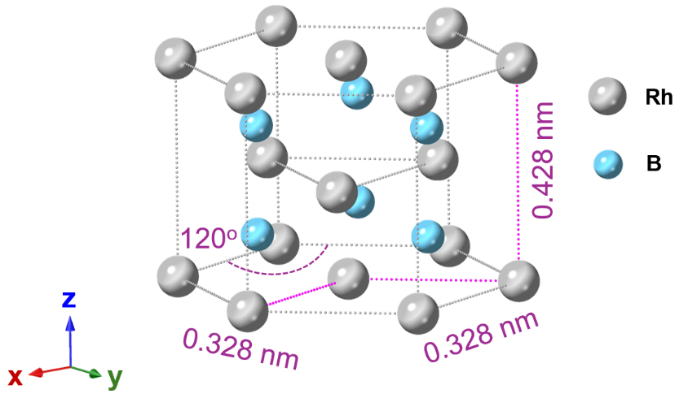


**Fig. S4** The crystal structure model of *hcp* RhB. The lattice parameters are derived from the inter-planer spacing d_hkl_ obtained by the XRD pattern and Bragg’s Law (λ = 2d_hkl_sinθ).

Note that *hcp* RhB crystallizes in the hexagonal *P*6₃/*mm*c space group. Rh is bonded in a 6-coordinate geometry to six equivalent B atoms. B is bonded in a distorted body-centered cubic geometry to six equivalent Rh and two equivalent B atoms [S3, S4].


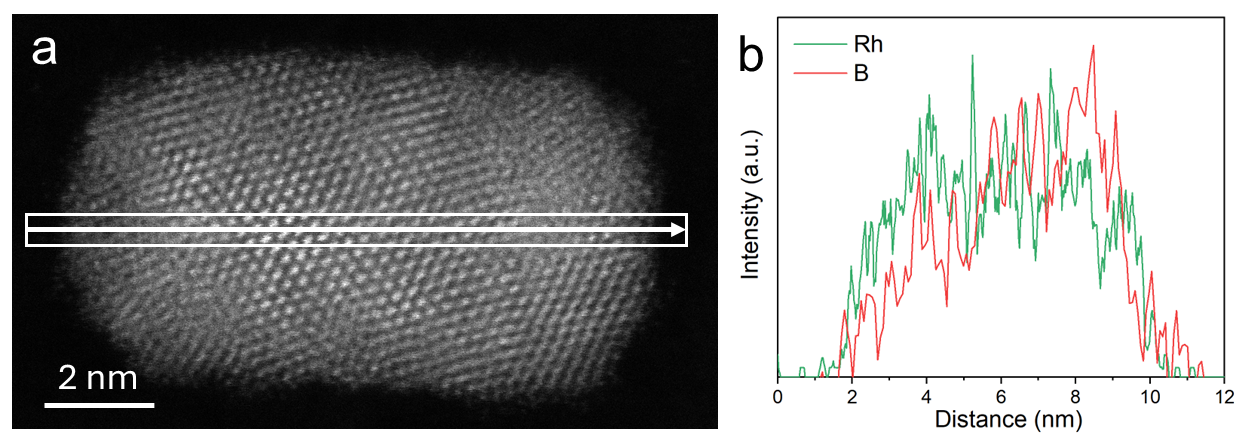


**Fig. S5** (**a**) HAADF-STEM image of one typical *hcp* RhB NP and (**b**) the corresponding line-scan STEM-EDS elemental analysis of Rh and EELS elemental analysis of B along the white arrow in (a)


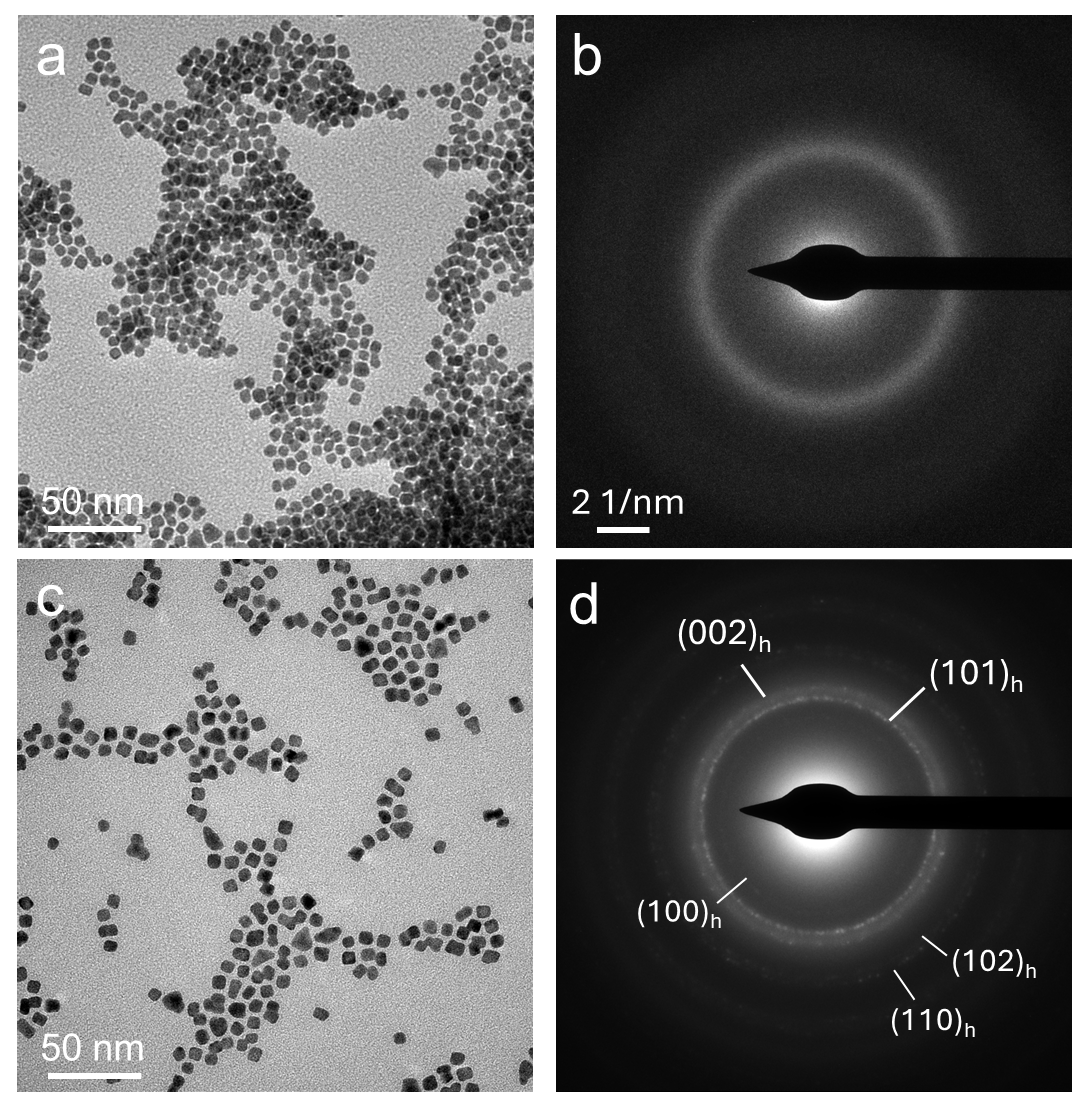


**Fig. S6** Structural characterizations of B-inserted Rh nanocrystals obtained using DMAB as an alternative B source. The synthesis temperature is increased, and other conditions are the same as those using BH_3_-THF. (**a**) TEM image and (**b**) SAED pattern of *a*-Rh_4_B NPs prepared at reaction temperature of 110 ^○^C. (**c**) TEM image and (d) SAED pattern of *hcp* RhB NPs prepared at reaction temperature of 170 ^○^C


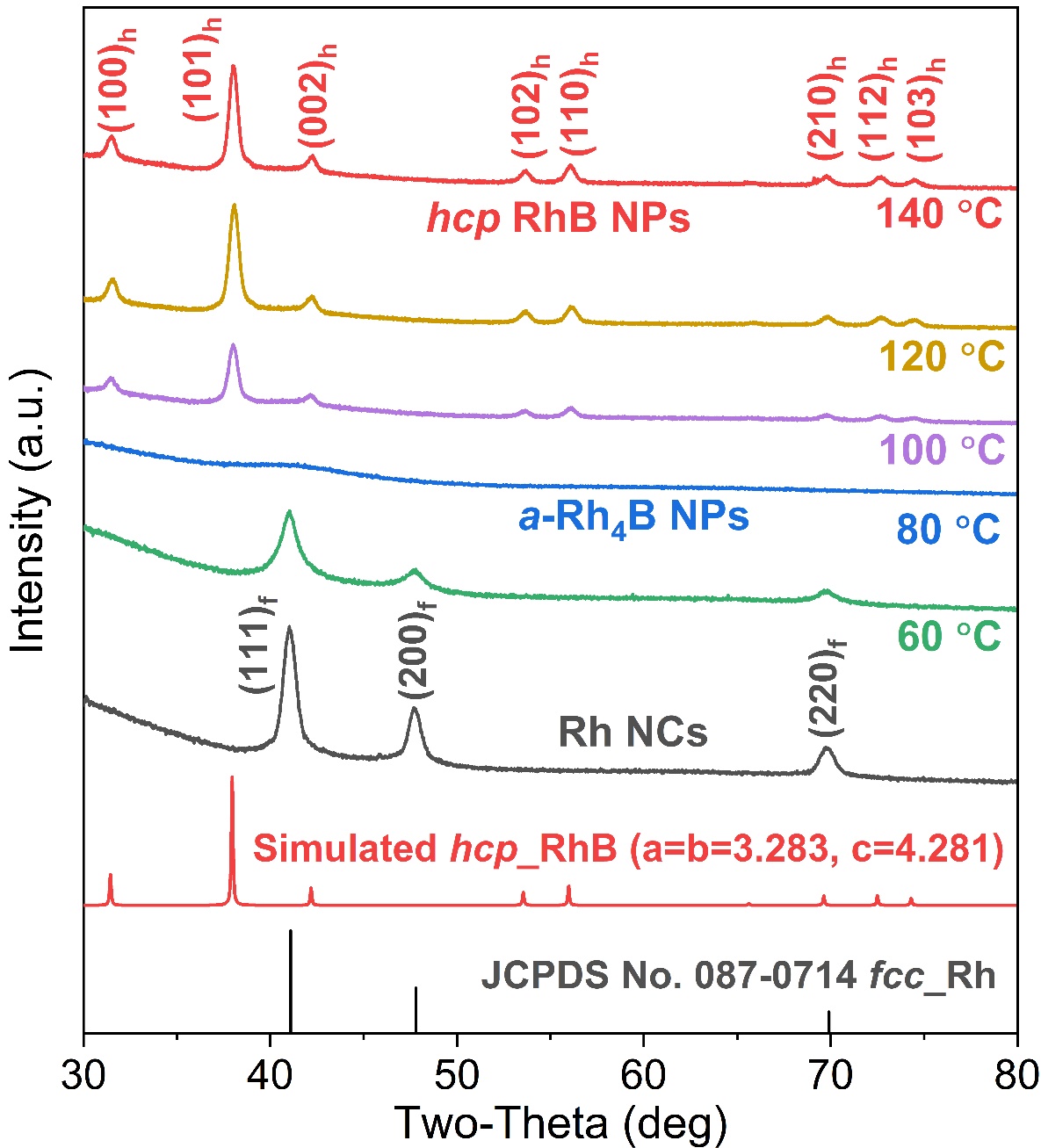


**Fig. S7** XRD patterns of B-inserted Rh nanomaterials synthesized at different reaction temperatures while other conditions are unchanged


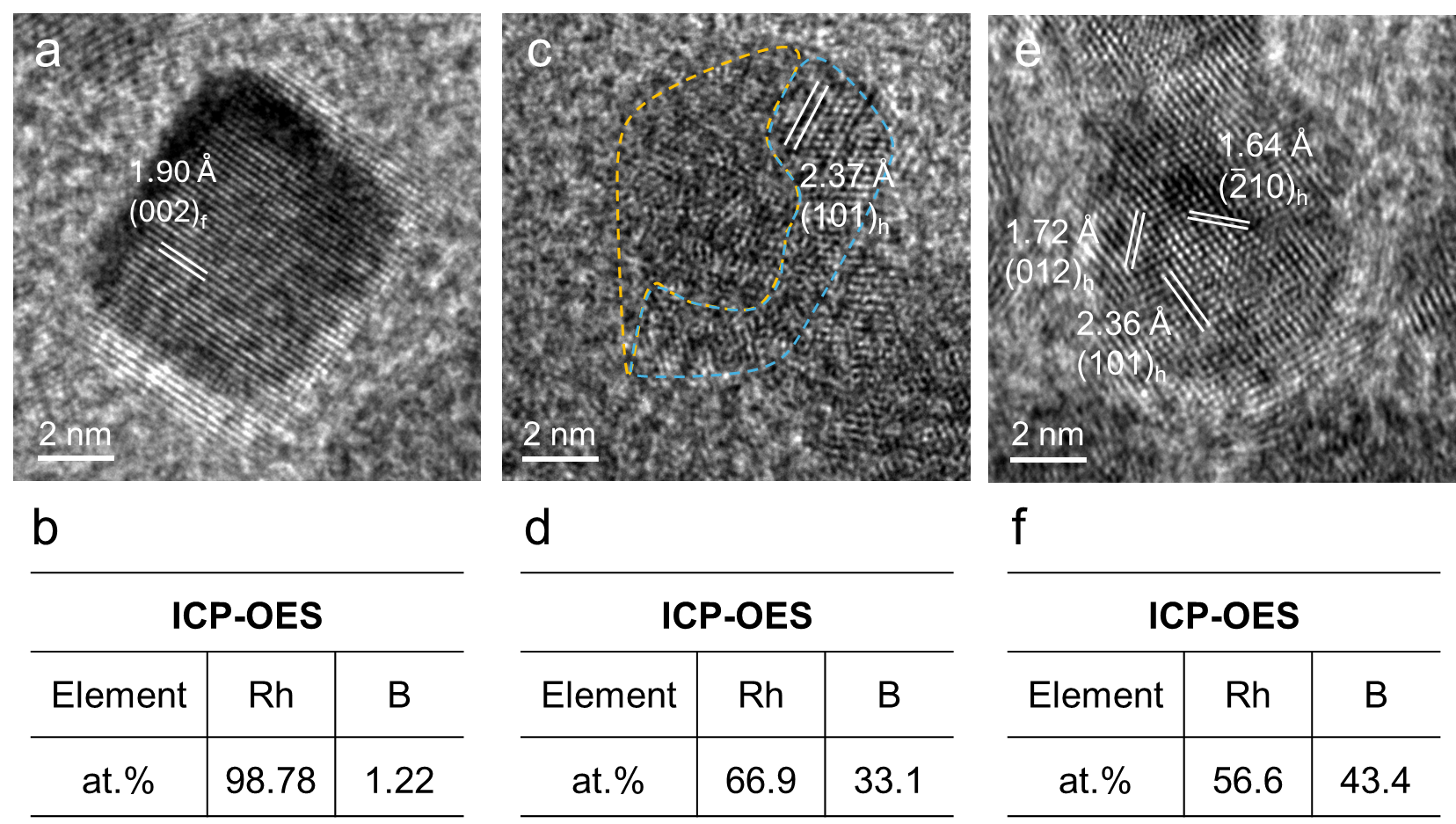


**Fig. S8** HRTEM images of B-inserted Rh nanocrystals synthesized at (**a**) 60 °C, (**c**) 100 °C, and (**e**) 120 °C, respectively. ICP-OES results of B-inserted Rh nanocrystals synthesized at (**b**) 60 °C, (**d**) 100 °C, and (**f)** 120 °C, respectively.


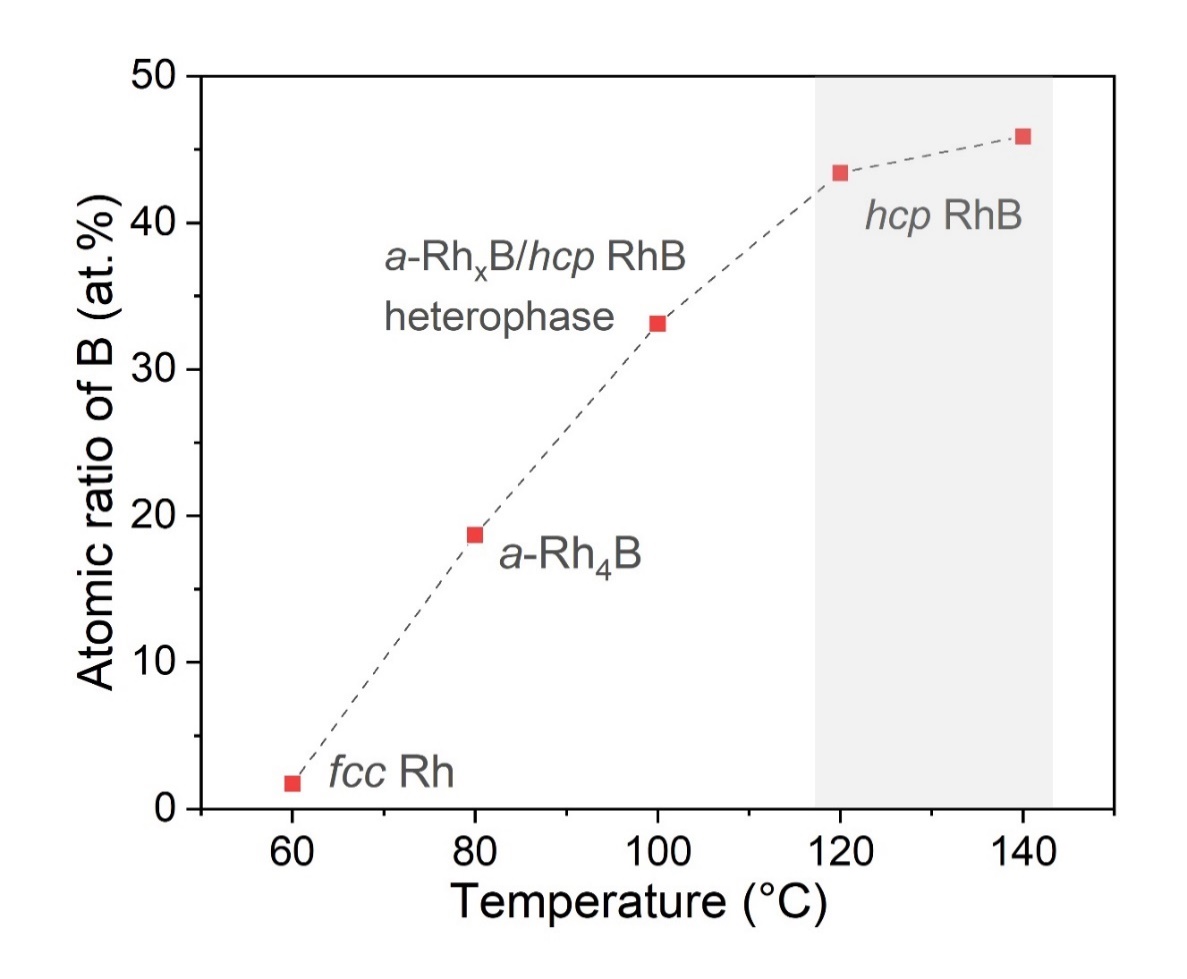


**Fig. S9** Temperature-composition correlation analysis of B-inserted Rh nanocrystals synthesized at different reaction temperatures while other conditions are unchanged. At 60 °C, B content was neglectable (1.22 at.%) and the obtained sample maintains the *fcc* phase, while an *fcc*-to-amorphous transition initiates between 60-80 °C. The coexistence of *a*-RhₓB and *hcp* RhB phases (*a*-RhₓB/*hcp* RhB heterophase, 33.1 at.% B) at 100 °C captures the intermediate transition state during B insertion. Furthermore, the complete transformation to phase-pure *hcp* RhB occurs above 120 °C, as the B content approximately exceeds 43.4 at.%


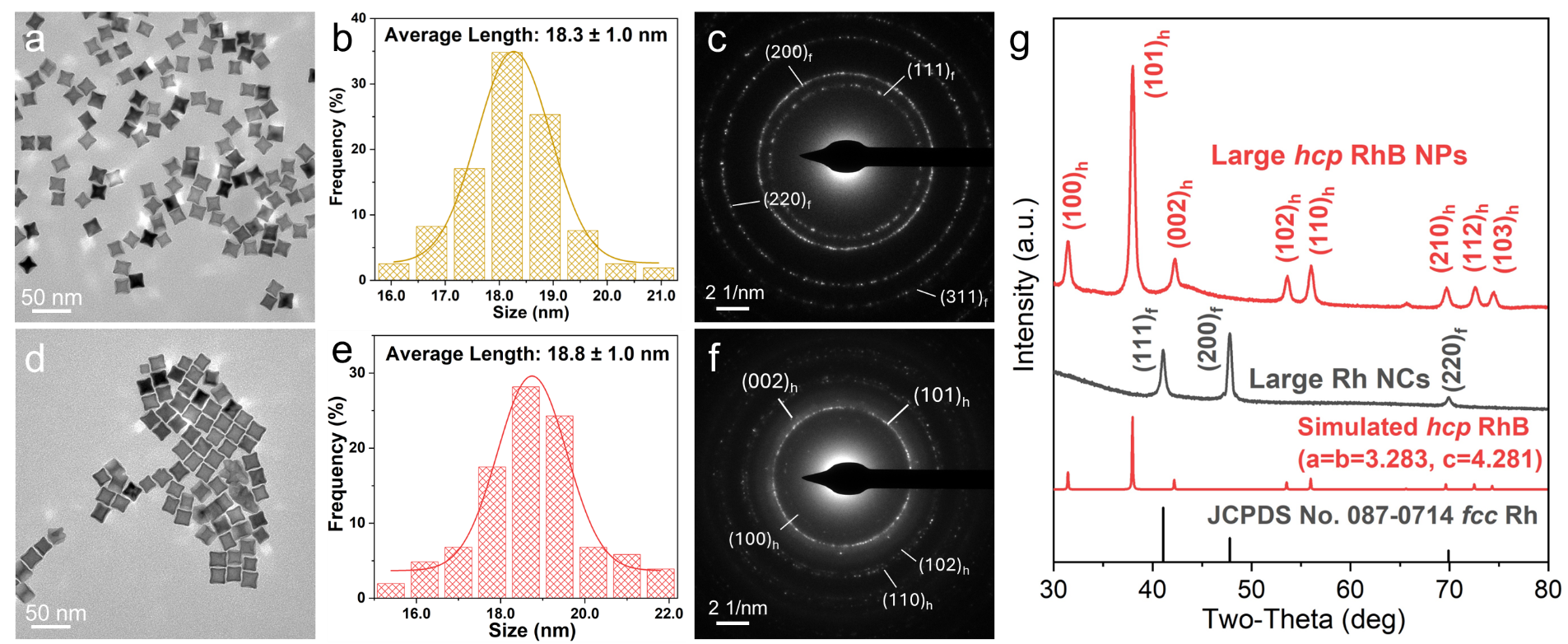


**Fig. S10** (**a, d**) TEM images of large Rh NCs and large *hcp* RhB NPs. (**b,** **c**) Size distribution diagrams of large Rh NCs and large *hcp* RhB NPs. (**e, f**) SAED patterns of large Rh NCs and large *hcp* RhB NPs. (**g**) XRD patterns of large Rh NCs and large *hcp* RhB NPs


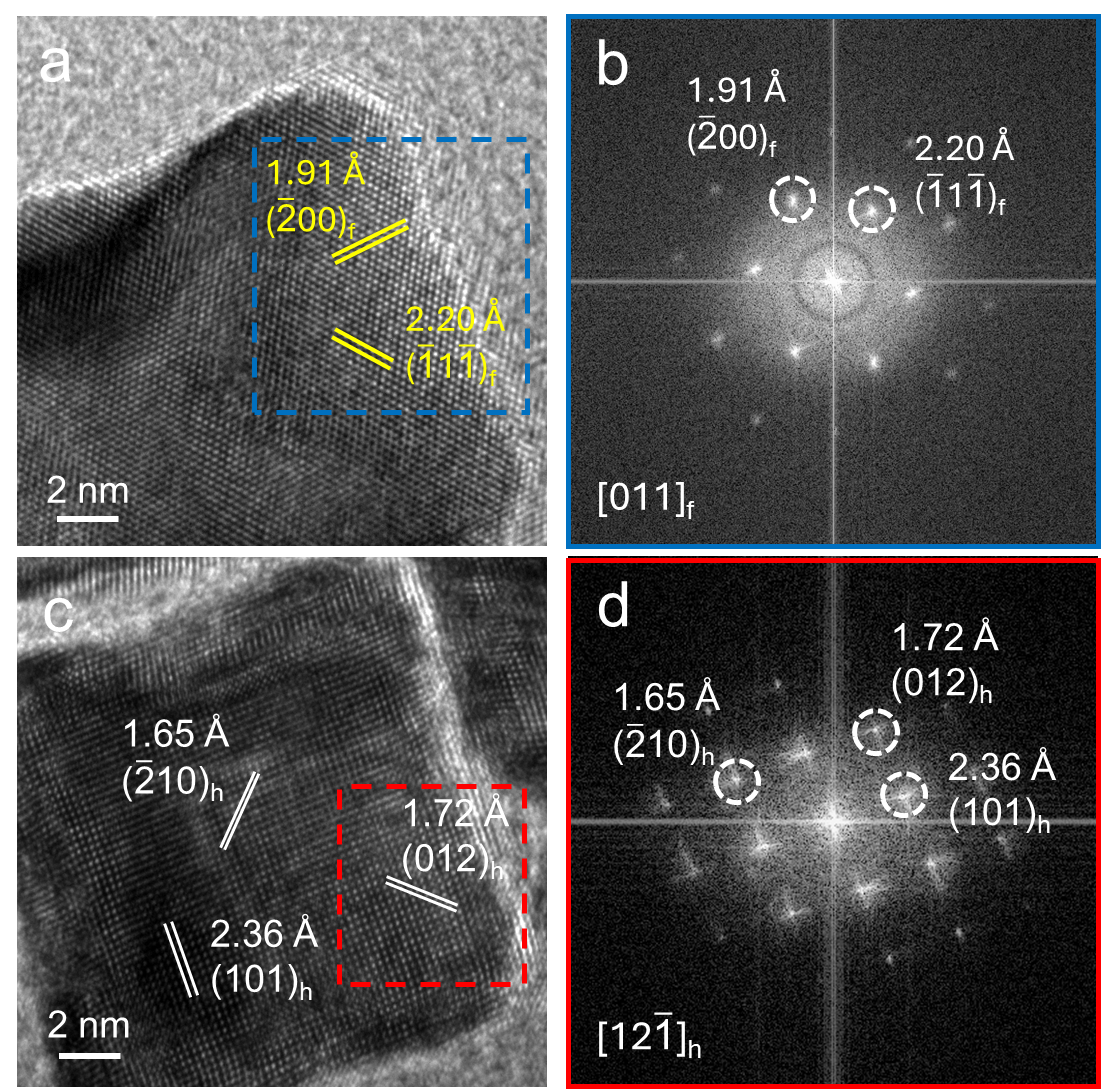


**Fig. S11** (**a**) HRTEM image and (**b**) The corresponding FFT pattern of the selected square area marked in (**a**) of large Rh NCs. (**c**) HRTEM image and (**d**) The corresponding FFT pattern of the selected square area marked in (**c**) of large *hcp* RhB NPs.


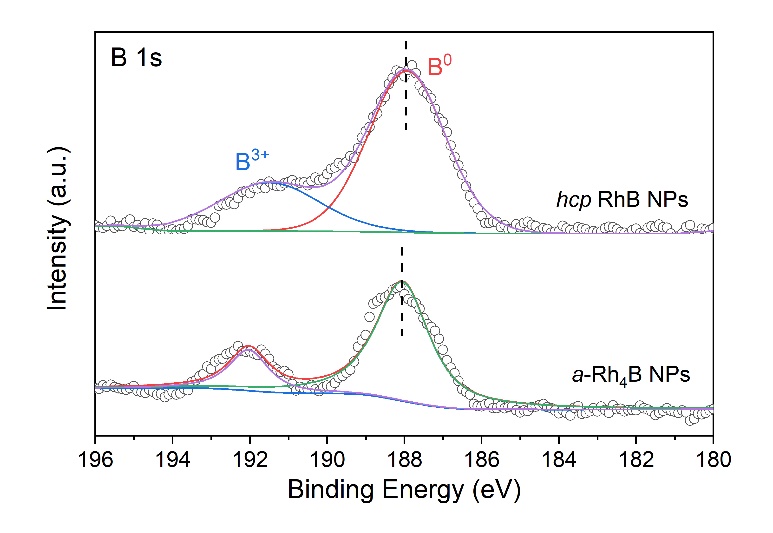


**Fig. S12** High-resolution XPS spectra of B 1s of *a*-Rh_4_B NPs and *hcp* RhB NPs. Note that mild surface B oxidation is commonly observed in B-inserted metal-based nanomaterials and a B oxidation peak is often present in XPS test [S5]


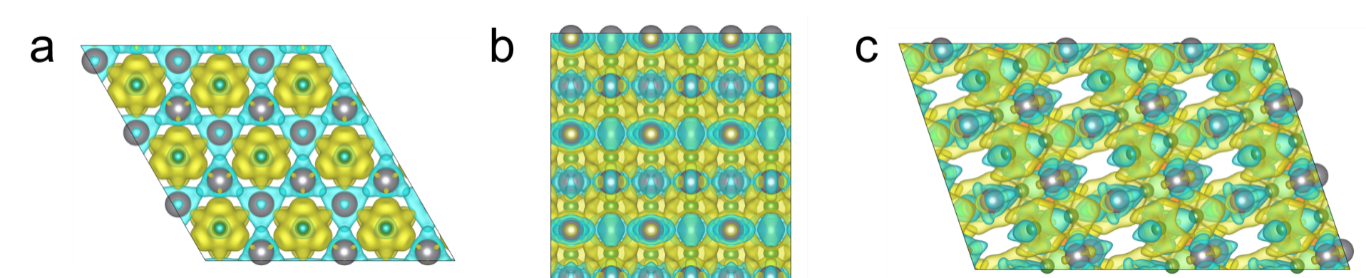


**Fig. S13** The differential charge density analysis of RhB(002), RhB(100), and RhB(101) facets (isovalue = 0.008 a.u.)


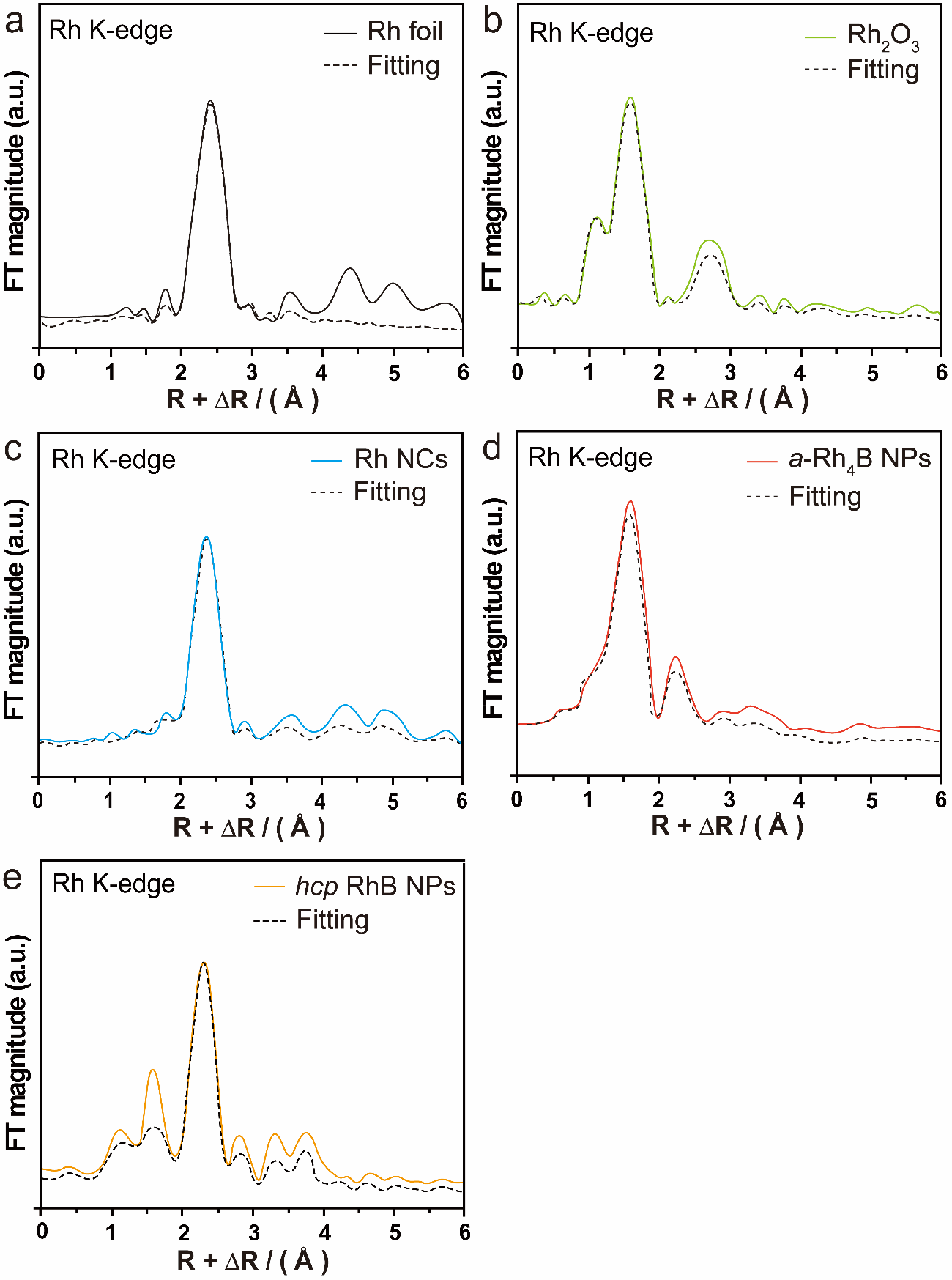


**Fig. S14** EXAFS fitting results of k^2^-weighted R space (a) Rh foil, (b) Rh_2_O_3_, (c) Rh NCs, (d) *a*-Rh_4_B NPs and (e) *hcp* RhB NPs


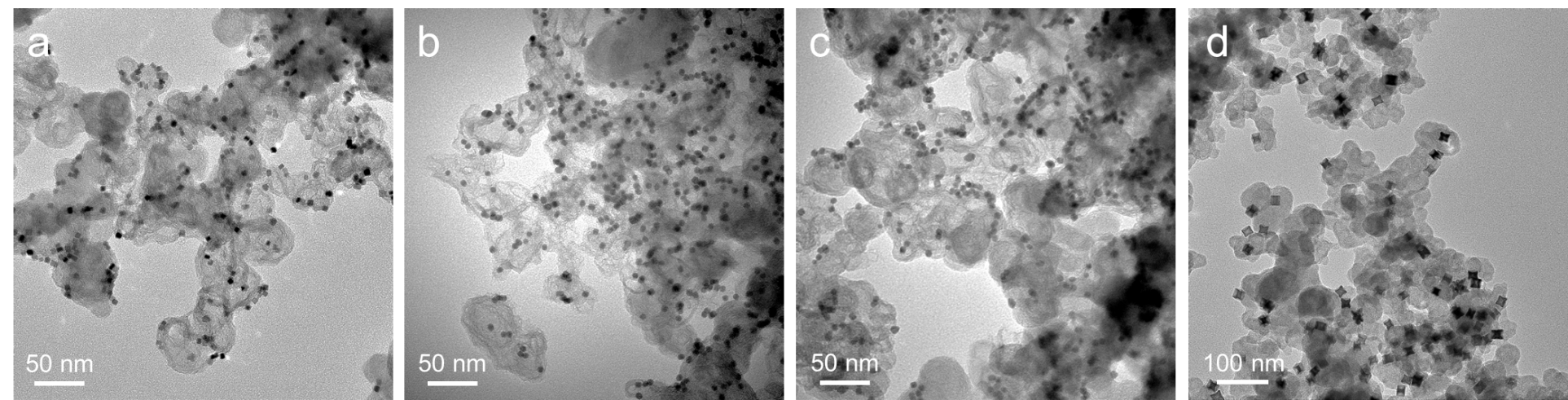


**Fig. S15** TEM images of (**a**) Rh NCs, (**b**) *a*-Rh_4_B NPs, (**c**) *hcp* RhB NPs, (**d**) large *hcp* RhB NPs loaded on carbon black


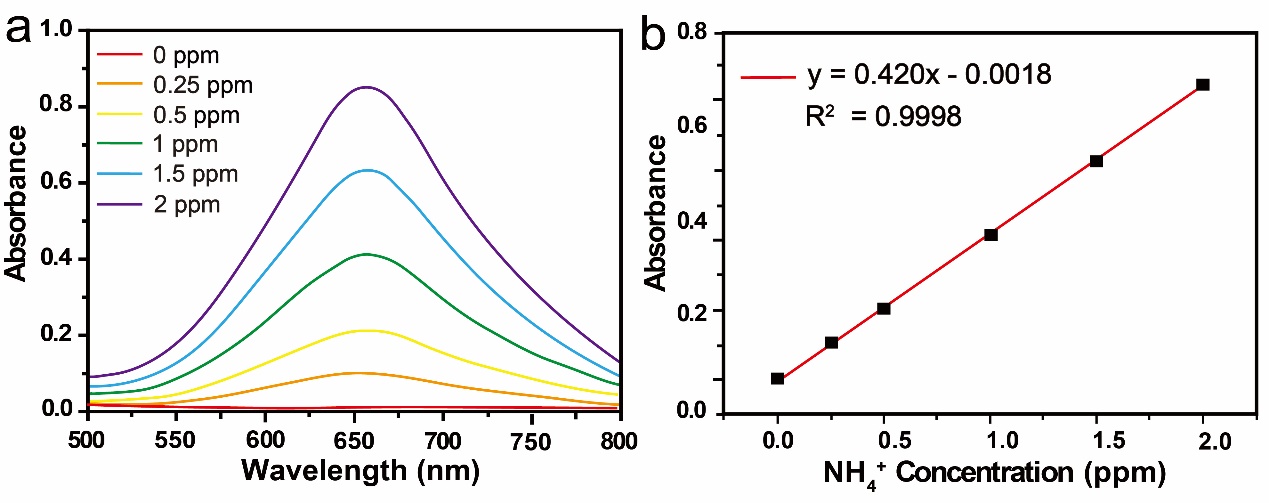


**Fig. S16** Quantification of NH_3_ using the indophenol blue by colorimetric method. (**a**) UV−Vis absorption spectra for NH_4_^+^ (a detectable amount of NH_3_, in the form of NH_4_^+^) at various concentrations. (**b**) A calibration curve for estimating the concentrations of NH_4_^+^


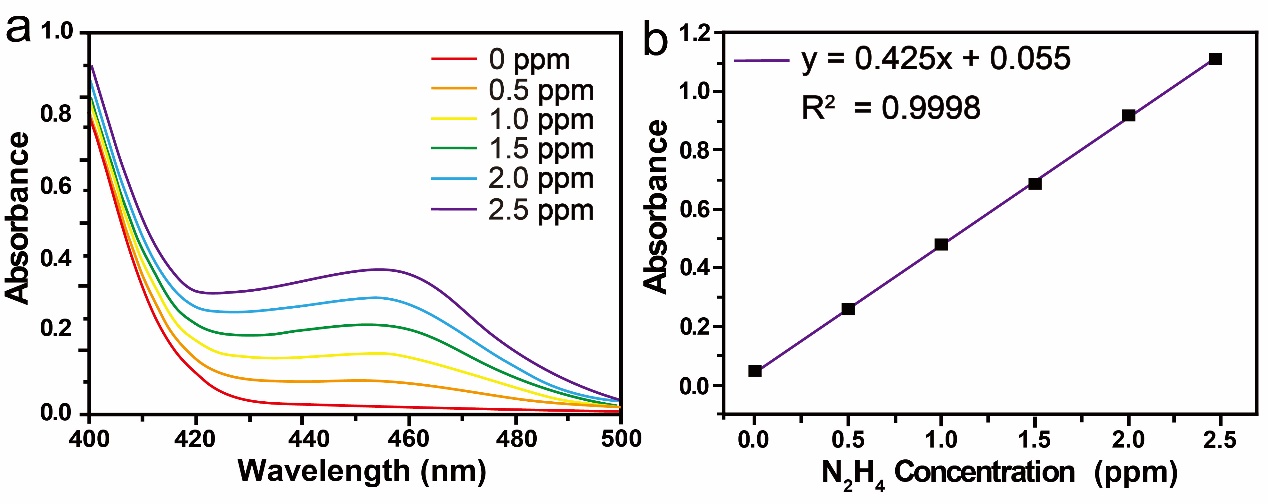


**Fig. S17** Quantification of N_2_H_4_ using colorimetric method. (**a**) UV-Vis absorption spectra of a series of N_2_H_4_ solutions at different concentrations after incubated for 20 min at room temperature and corresponding (**b**) calibration curve used for estimating N_2_H_4_


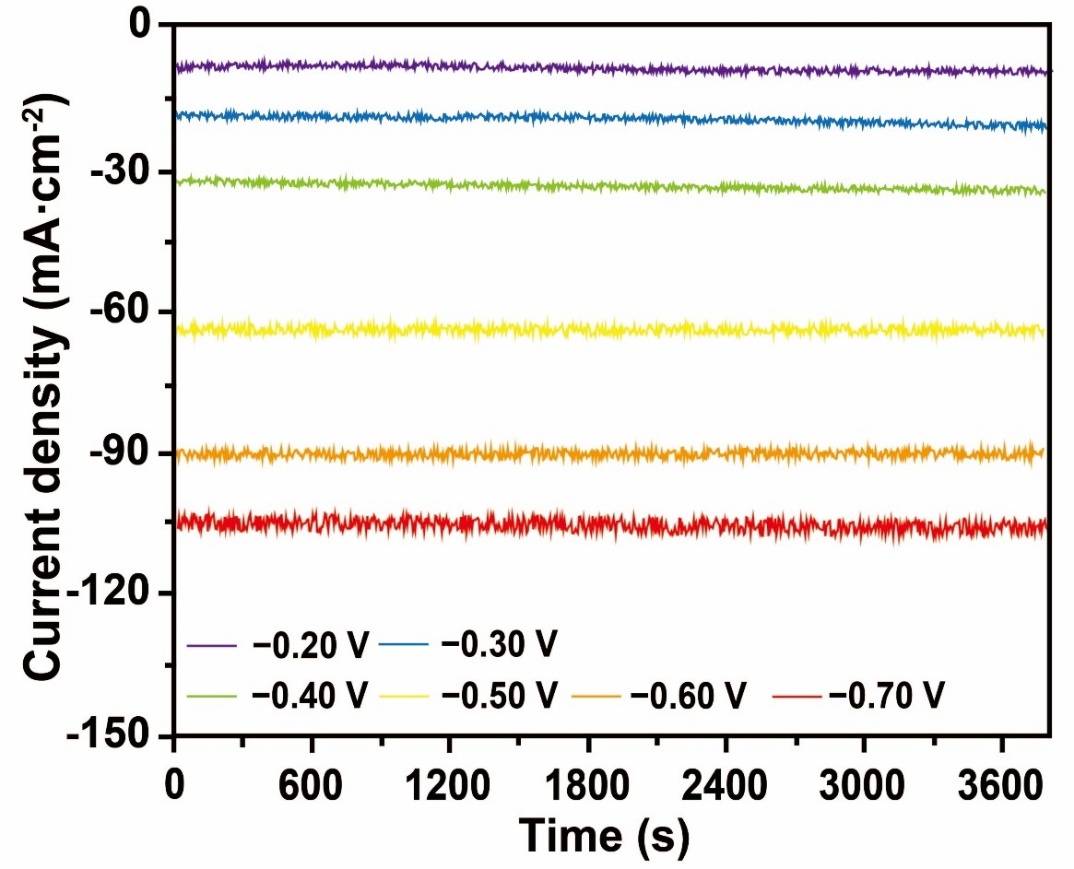


**Fig. S18** Chronoamperometry curves for *hcp* RhB NPs at various potentials in NO (20 *vol.*%) with 0.5 M Na_2_SO_4_ electrolyte


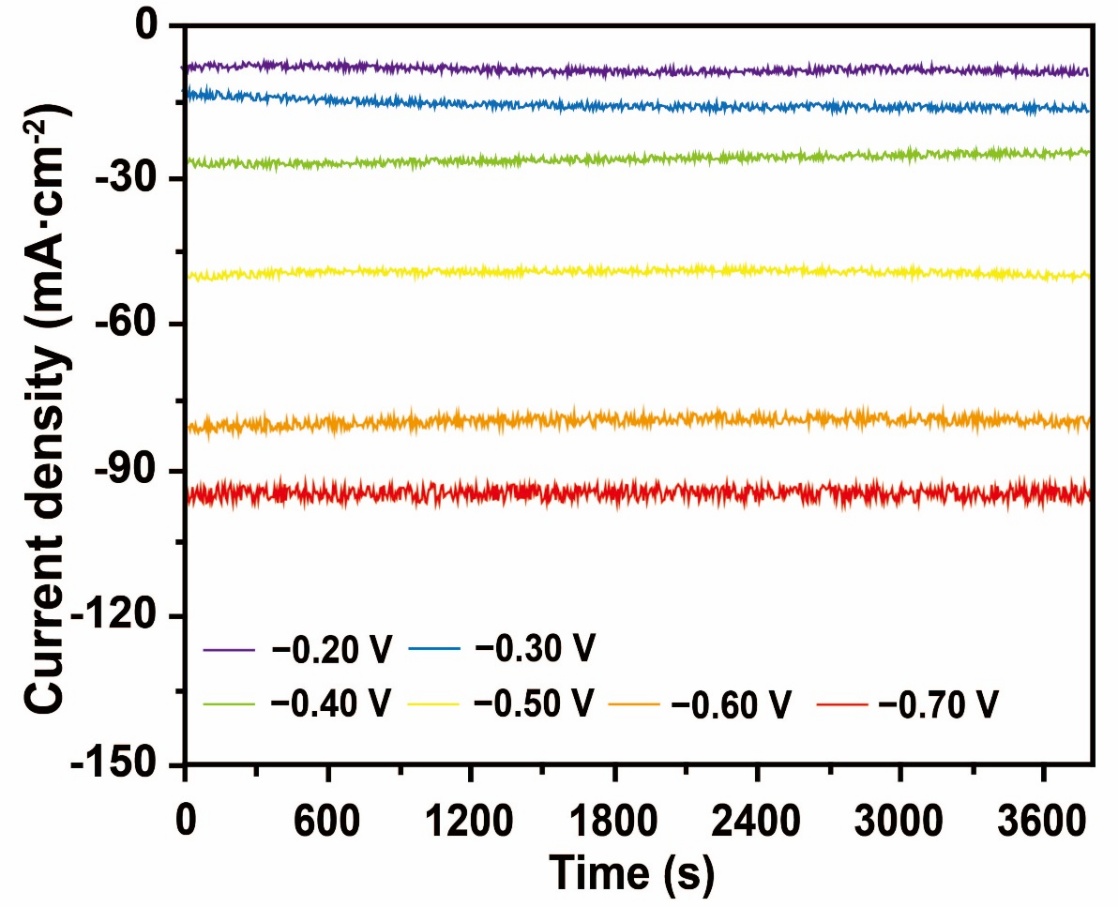


**Fig. S19** Chronoamperometry curves for Rh NCs at various potentials in NO (20 *vol.*%) with 0.5 M Na_2_SO_4_ electrolyte


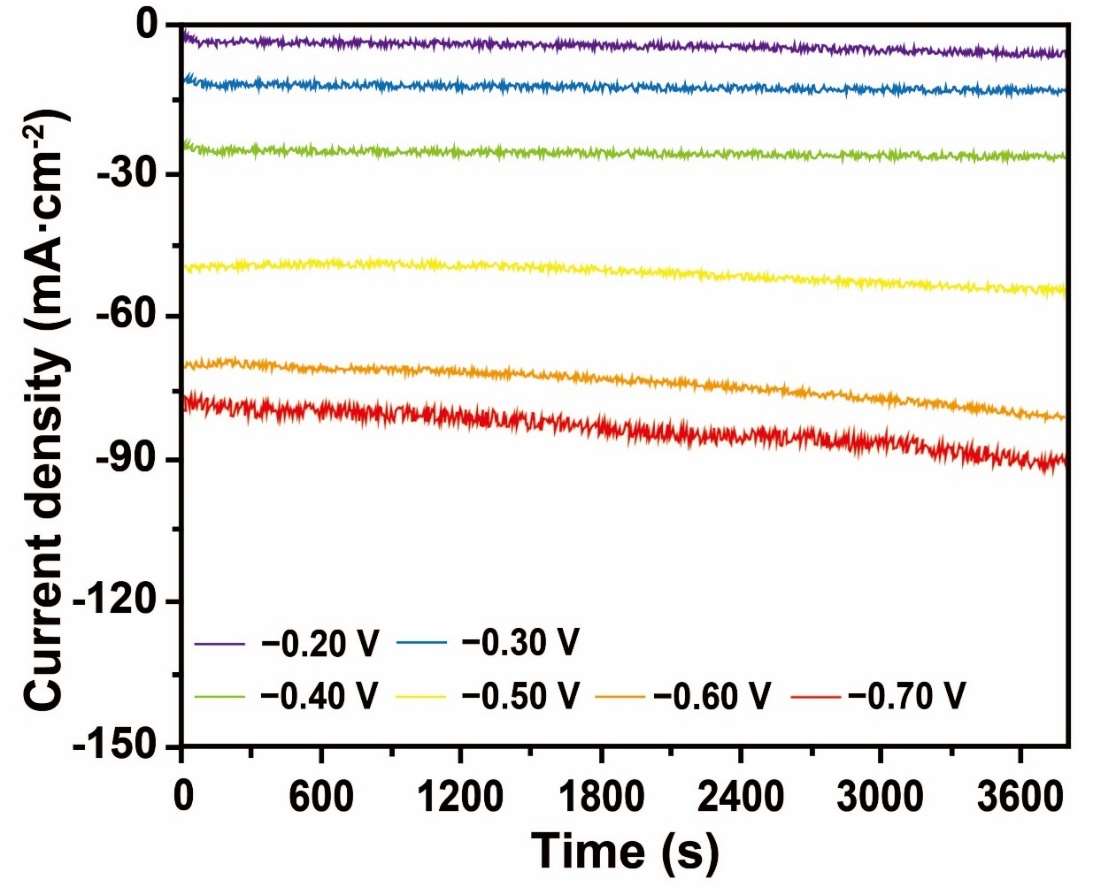


**Fig. S20** Chronoamperometry curves for *a*-Rh_4_B NPs at various potentials in NO (20 *vol.*%) with 0.5 M Na_2_SO_4_ electrolyte


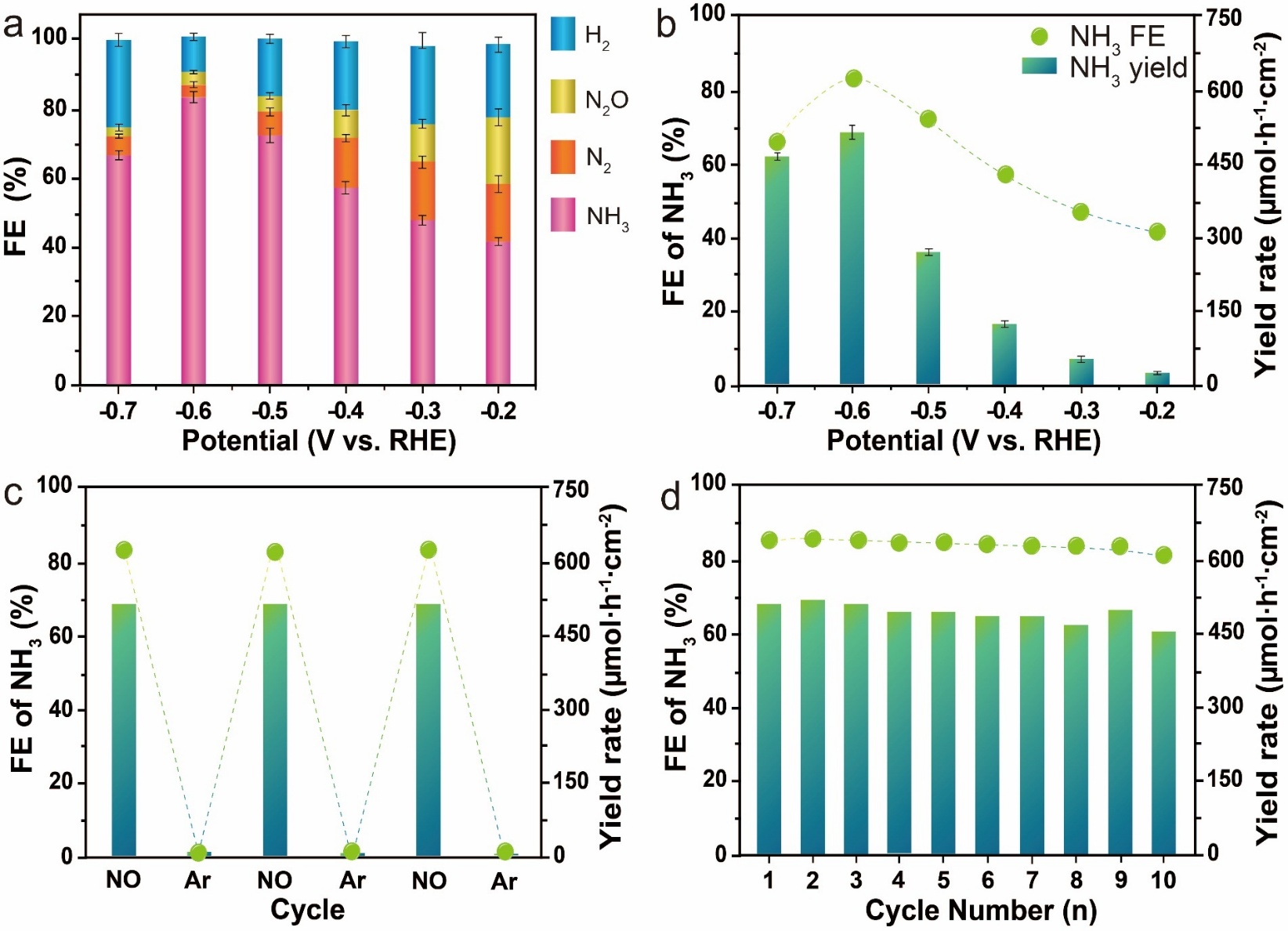


**Fig. S21** Electrocatalytic NORR performance of Rh NCs. (**a**) FEs of all possible products including NH_3_, H_2_, N_2_O and N_2_ for Rh NCs at each given potential. (**b**) NH_3_ yield and FE_NH3_ of Rh NCs at different potentials. (**c**) Alternating electrolysis test of Rh NCs at −0.6 V vs. RHE. (**d**) FE_NH3_ and NH_3_ yield rate in 10 consecutive cycles over Rh NCs for NORR at −0.6 V vs. RHE


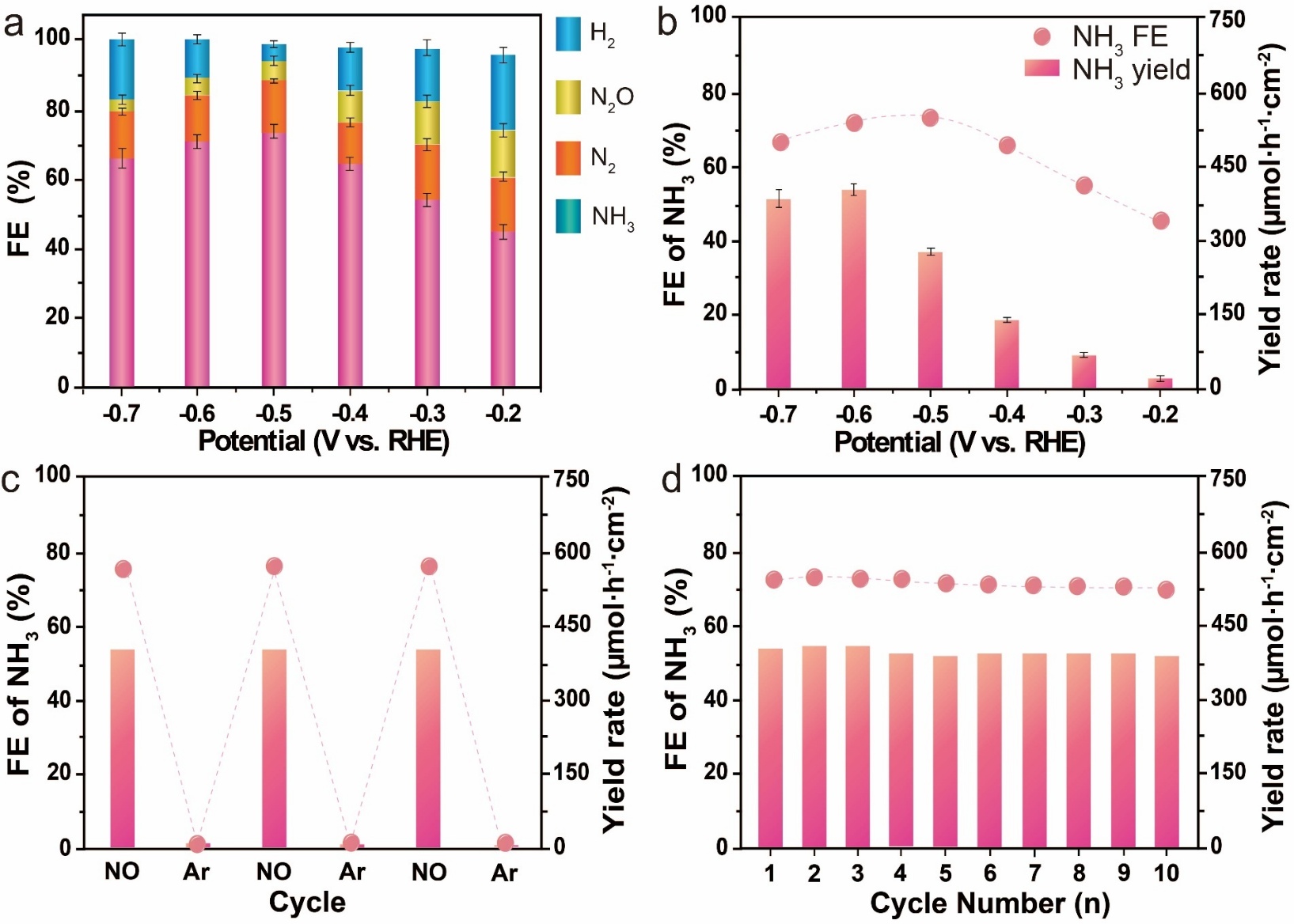


**Fig. S22** Electrocatalytic NORR performance of *a*-Rh_4_B NPs. (**a**) FEs of all possible products including NH_3_, H_2_, N_2_O and N_2_ for *a*-Rh_4_B NPs at each given potential. (**b**) NH_3_ yield and FE_NH3_ of *a*-Rh_4_B NPs at different potentials. (**c**) Alternating electrolysis test of *a*-Rh_4_B NPs at −0.6 V vs. RHE. (**d**) FE_NH3_ and NH_3_ yield rate in 10 consecutive cycles over *a*-Rh_4_B NPs for NORR at −0.6 V vs. RHE


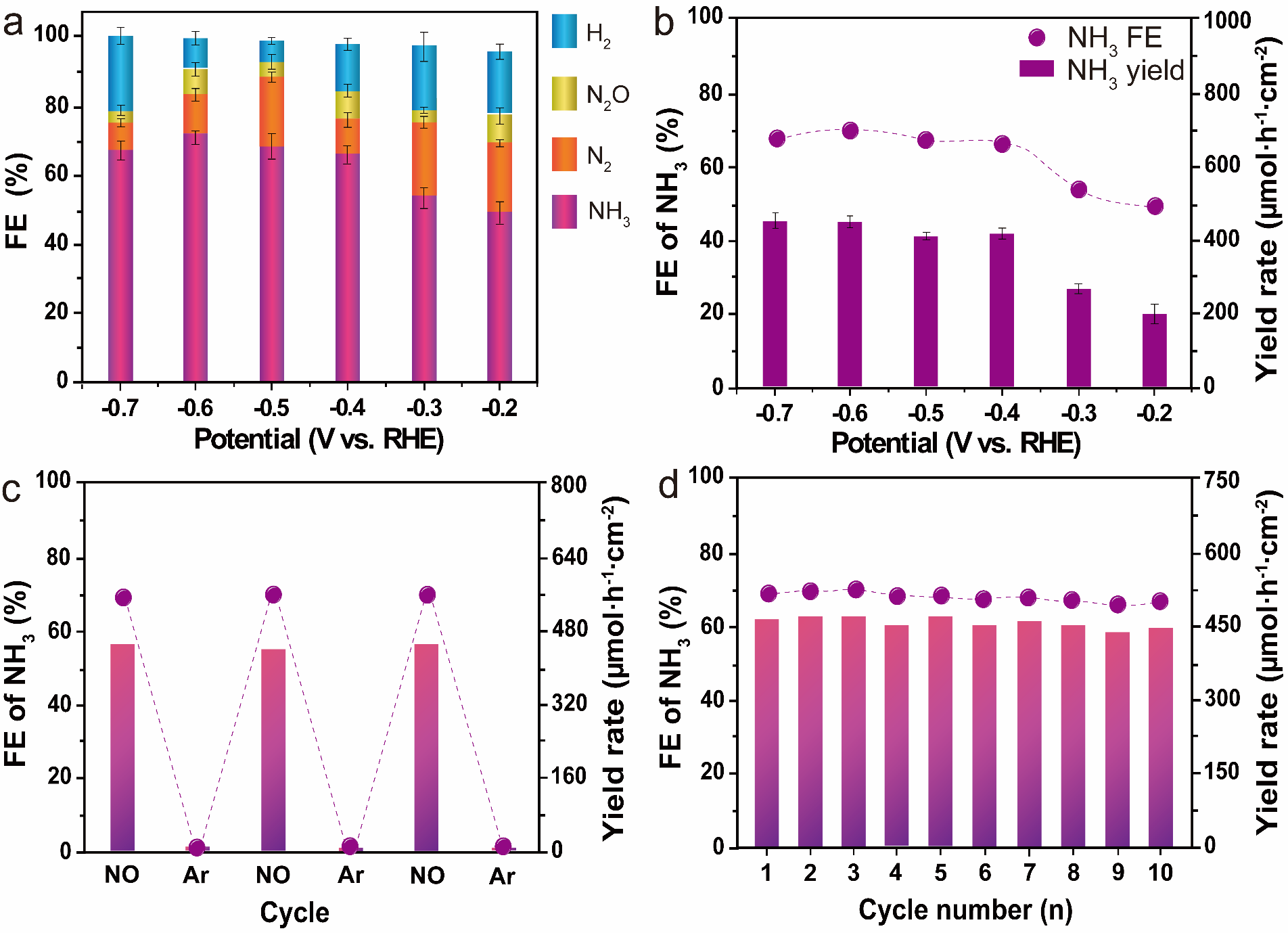


**Fig. S23** Electrocatalytic NORR performance of large *hcp* RhB NPs. (**a**) FEs of all possible products including NH_3_, H_2_, N_2_O and N_2_ at each given potential. (**b**) NH_3_ yield and FE_NH3_ measured at different potentials. (**c**) Alternating electrolysis test at −0.6 V vs. RHE. (**d**) FE_NH3_ and NH_3_ yield rate in 10 consecutive cycles for NORR at −0.6 V vs. RHE


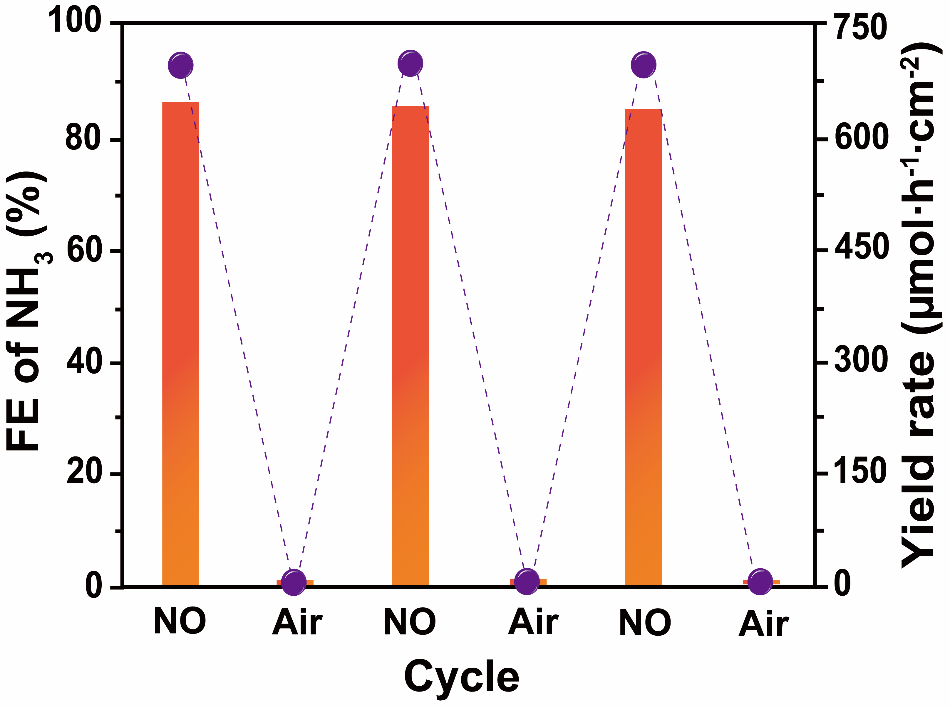


**Fig. S24** Alternating electrolysis test of *hcp* RhB NPs at −0.6 V vs. RHE by switching between NO-saturated and air-saturated electrolytes


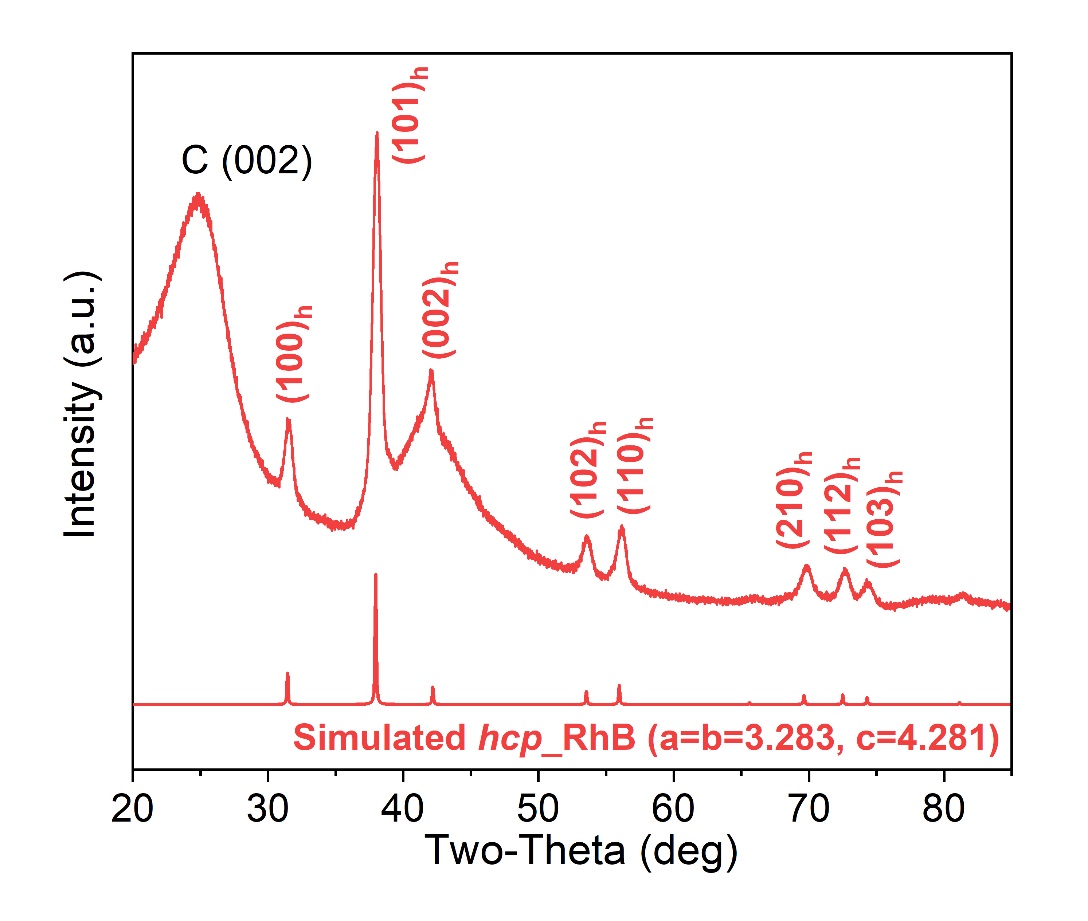


**Fig. S25** XRD pattern of *hcp* RhB NPs/C after the long-term electrocatalytic stability test


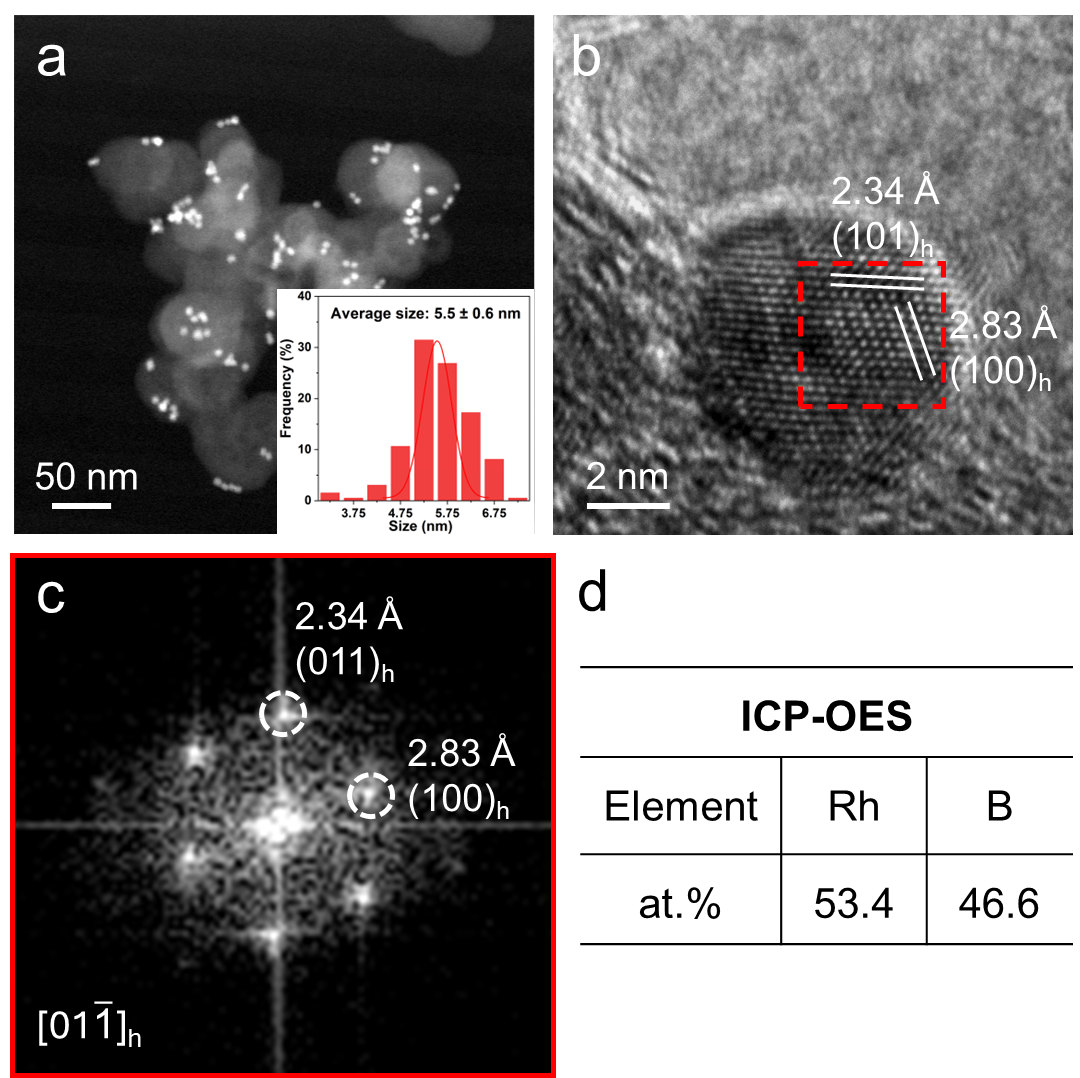


**Fig. S26** (**a**) HAADF-STEM image and the size distribution diagram (inset), (**b**) HRTEM image, (**c**) The corresponding FFT pattern of the selected area in (**b**), and (**d**) ICP-OES result of the *hcp* RhB NPs after the long-term electrocatalytic stability test


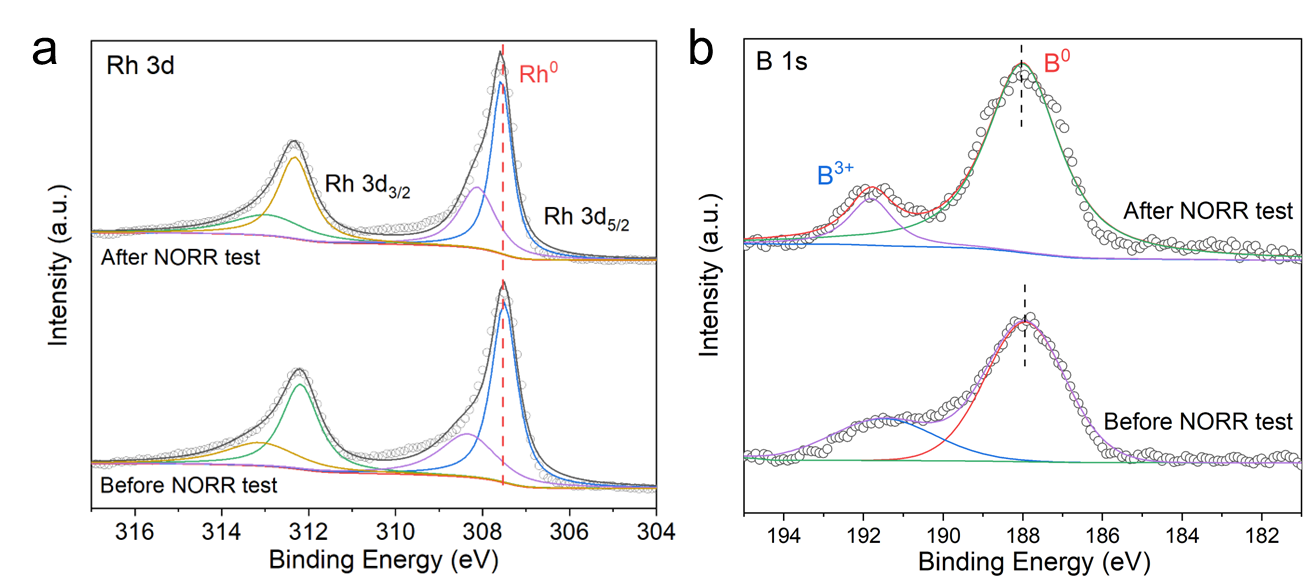


**Fig. S27** High-resolution XPS spectra of *hcp* RhB NPs before and after NORR test in (**a**) Rh 3d region, and (**b**) B 1s region


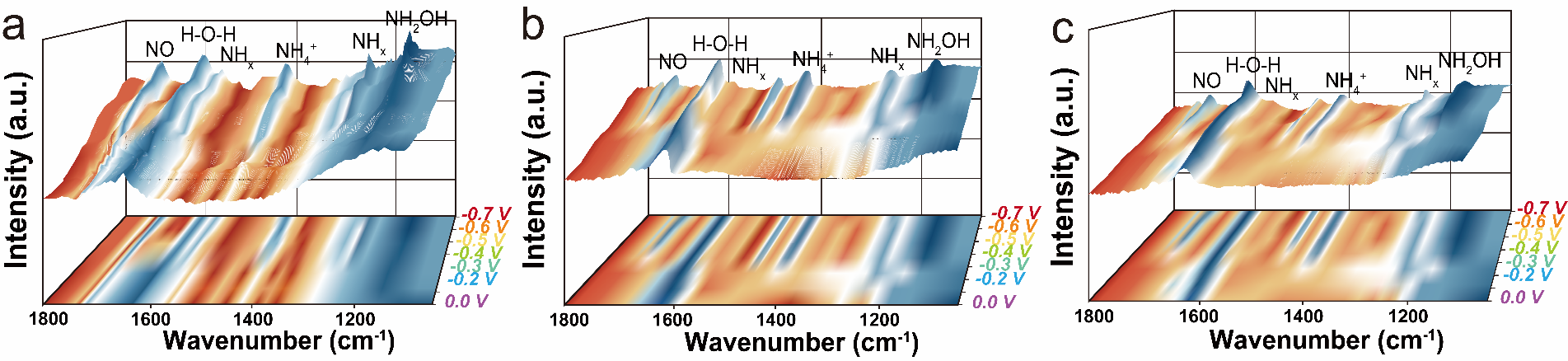


**Fig. S28** 3D potential-dependent *in situ* attenuated total reflection infrared spectra of (**a**) *hcp* RhB NPs, (**b**) *a*-Rh_4_B NPs, and (**c**) Rh NCs for NORR in the range from 0 to −0.7 V vs. RHE


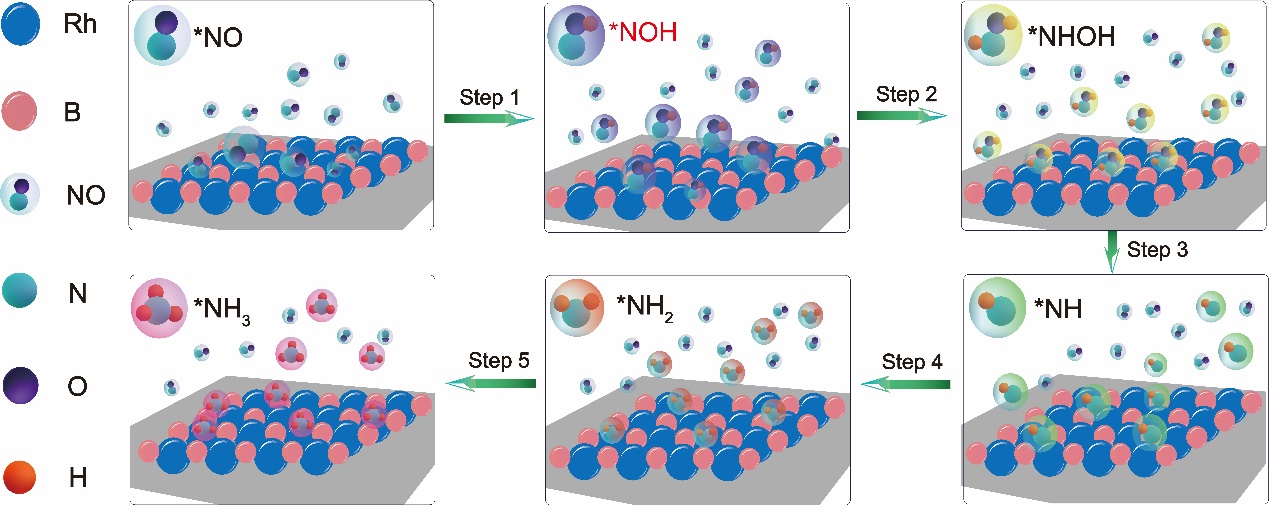


**Fig. S29** Schematic illustration of the first possible NORR pathway of “NO → *NO → *NOH → *NHOH → *NH_2_OH → *NH_2_ → *NH_3_”


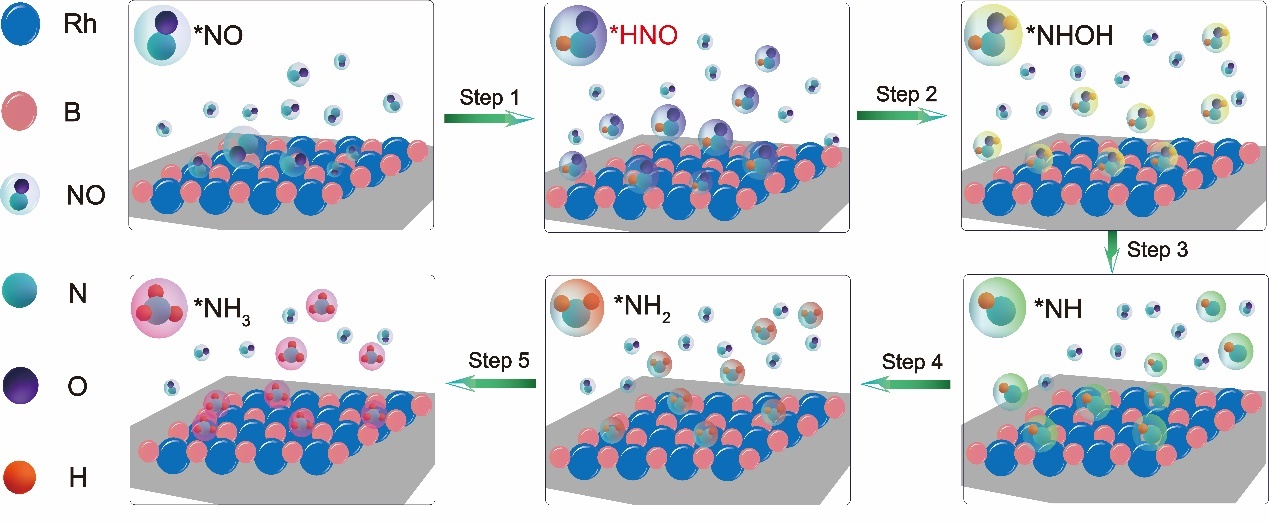


**Fig. S30** Schematic illustration of the second possible NORR pathway of “NO → *NO → *HNO → *NHOH → *NH_2_OH → *NH_2_ → *NH_3_”


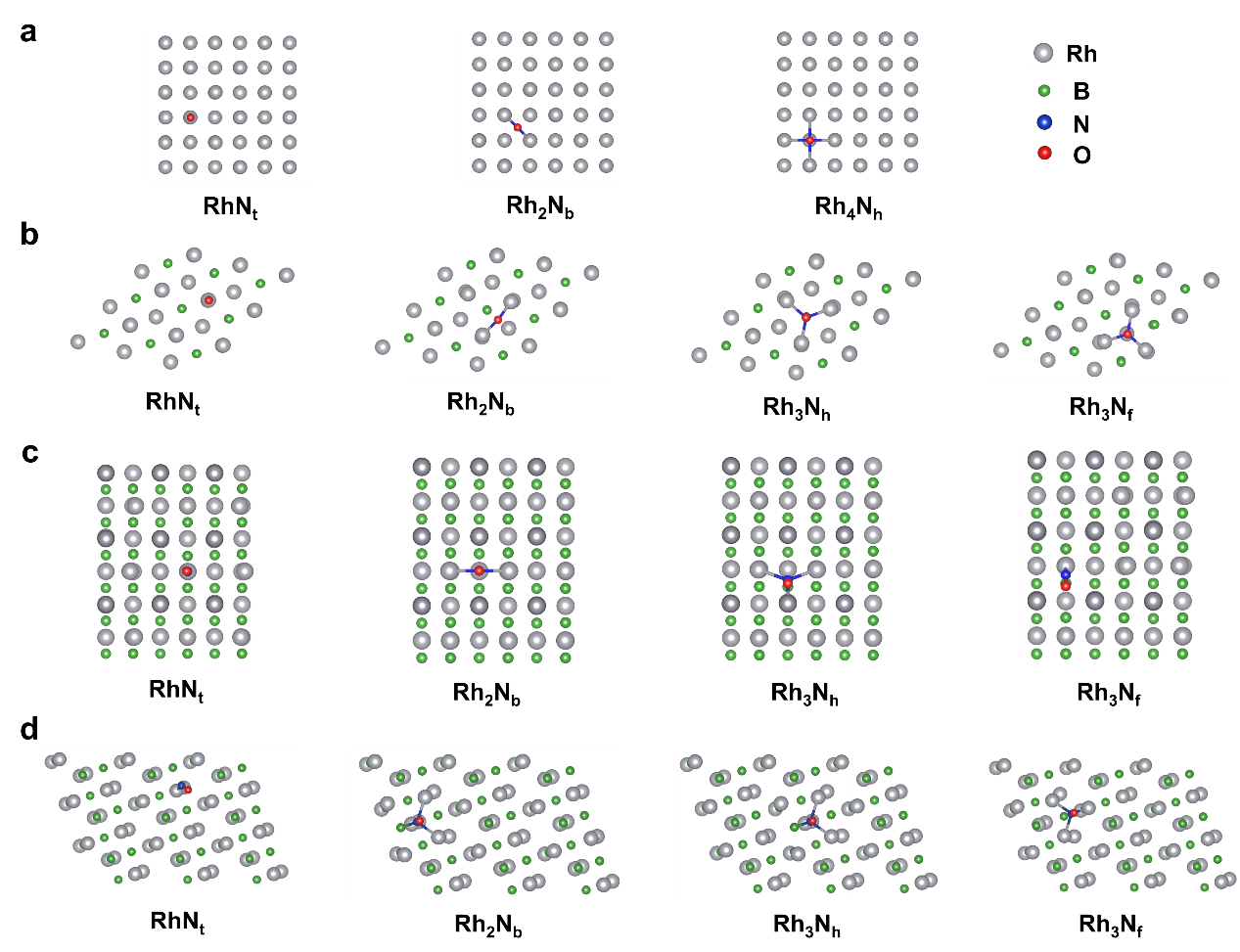


**Fig. S31** Top views of the possible adsorption configurations of NO molecules on the surfaces of (**a**) Rh(100); (**b**) RhB(002), (**c**) RhB(100), and (**d**) RhB(101) facets


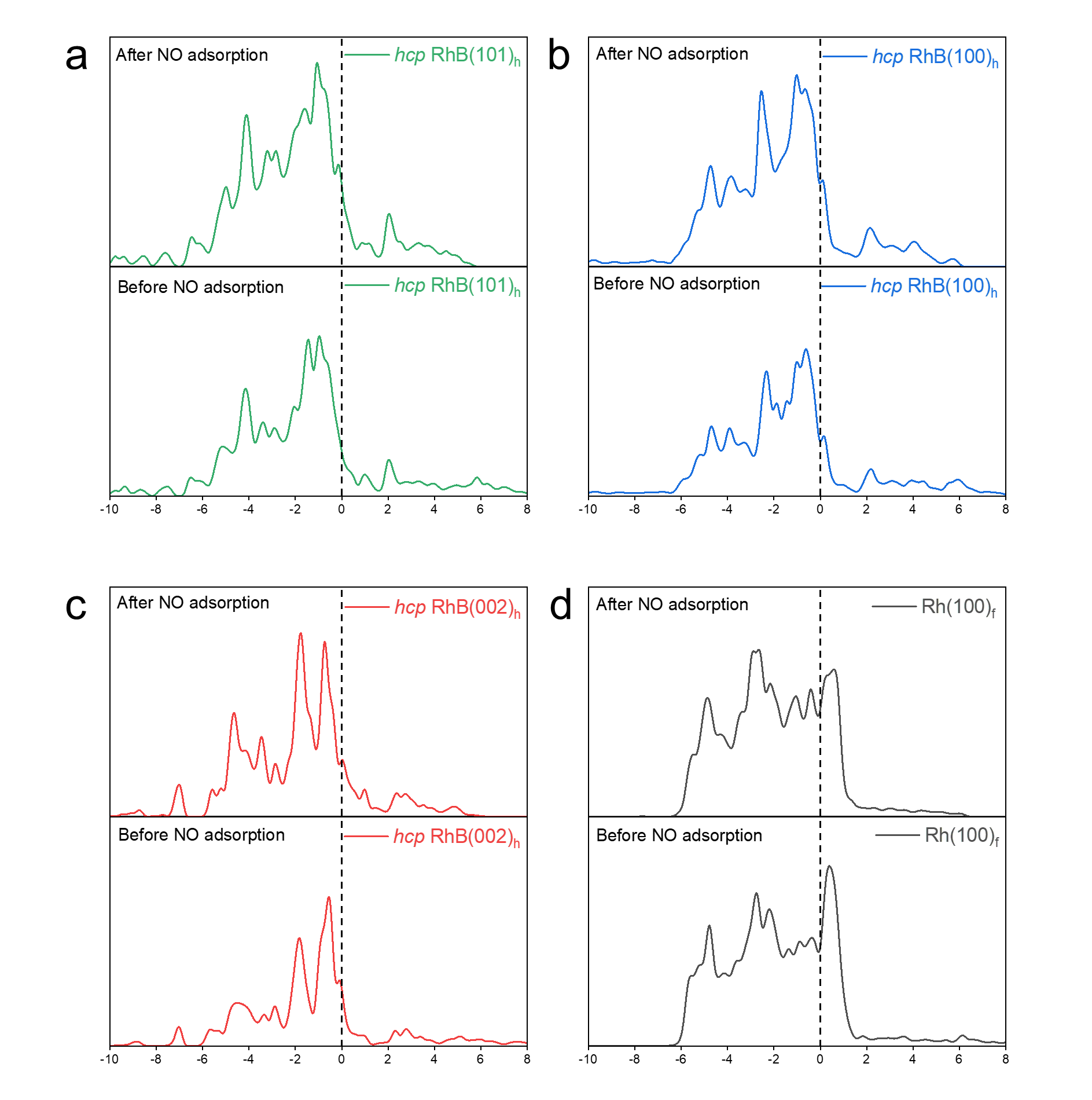


**Fig. S32** The PDOSs of Rh sites over (**a**) *hcp* RhB(101)_h_, (**b**) *hcp* RhB(100)_h_, (**c**) *hcp* RhB(002)_h_, and (**d**) Rh(100)_f_ surfaces before and after NO adsorption


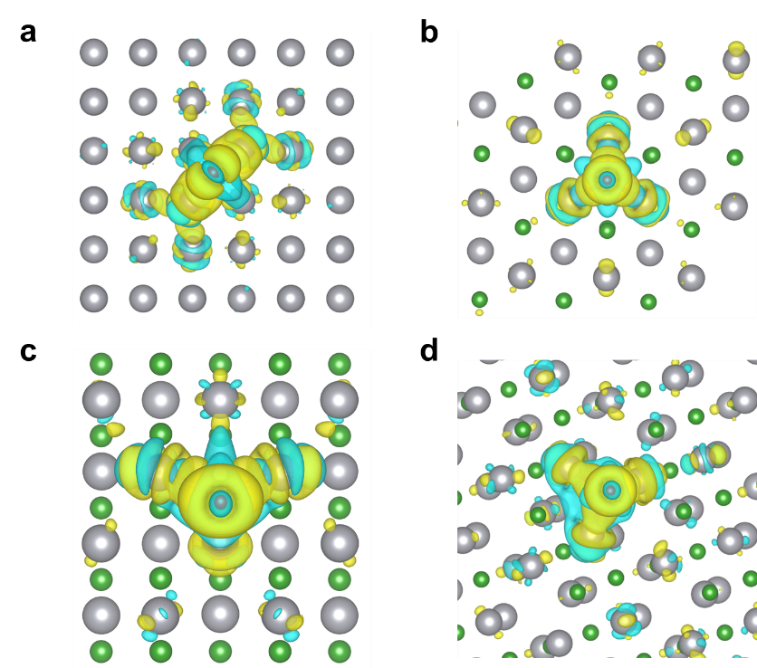


**Fig. S33** Top views of calculated Charge of Density and Bader Charge analysis of NO molecules on (**a**) Rh(100), (**b**) RhB(002), (**c**) RhB(100), and (**d**) RhB(101) facets


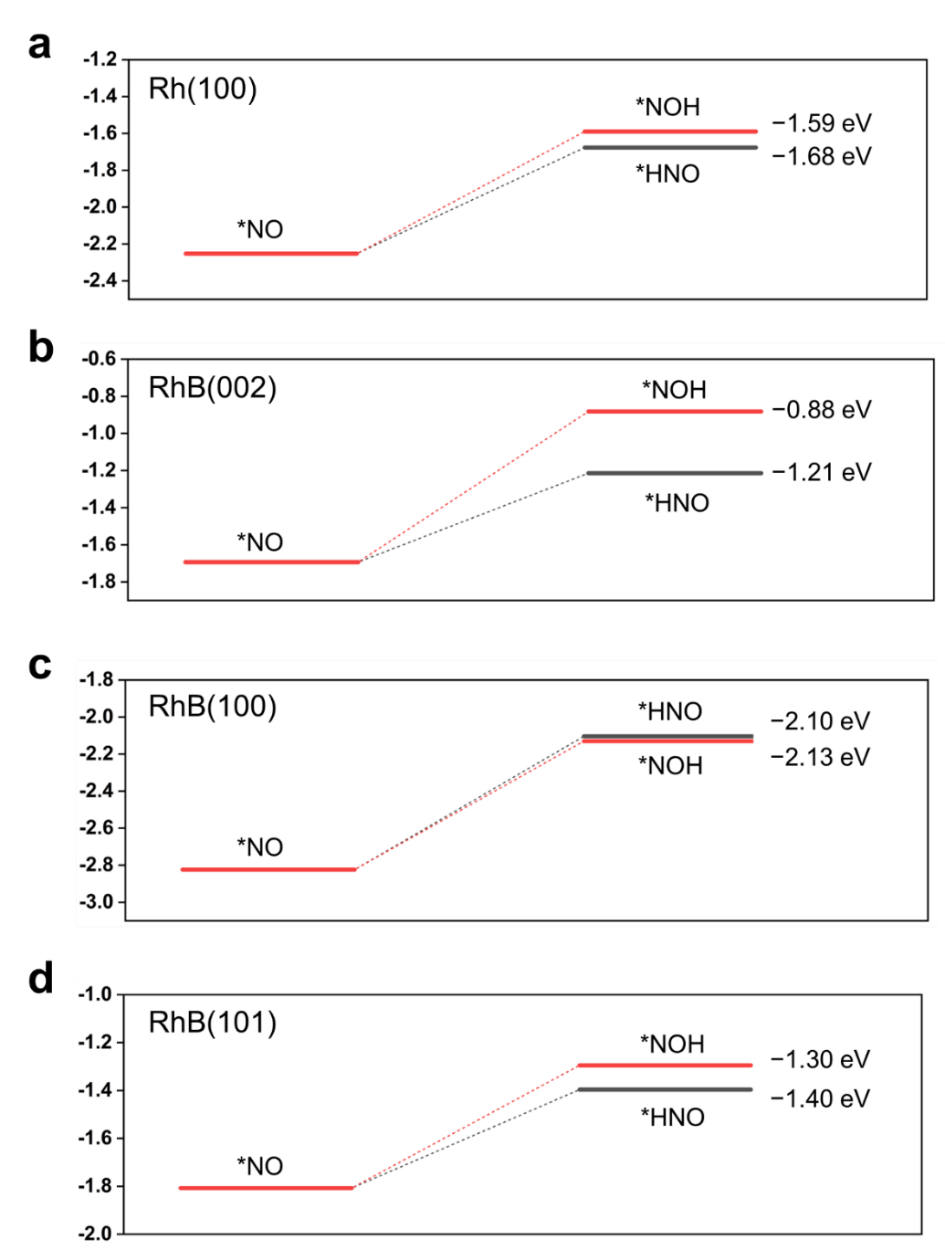


**Fig. S34** Reaction Gibbs free energy (G) diagram of “*NO → *HNO” step or “*NO → *NOH” step on (**a**) Rh(100), (**b**) RhB(002), (**c**) RhB(100), and (**d**) RhB(101) facets


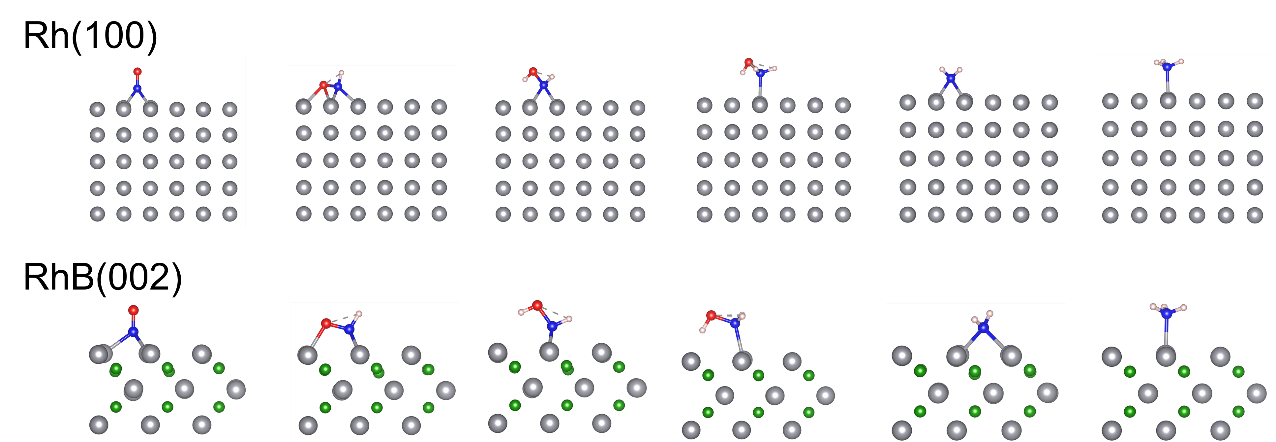


**Fig. S35** Side views of adsorption configurations of intermediates along the NORR steps on Rh(100) and RhB (002) facets


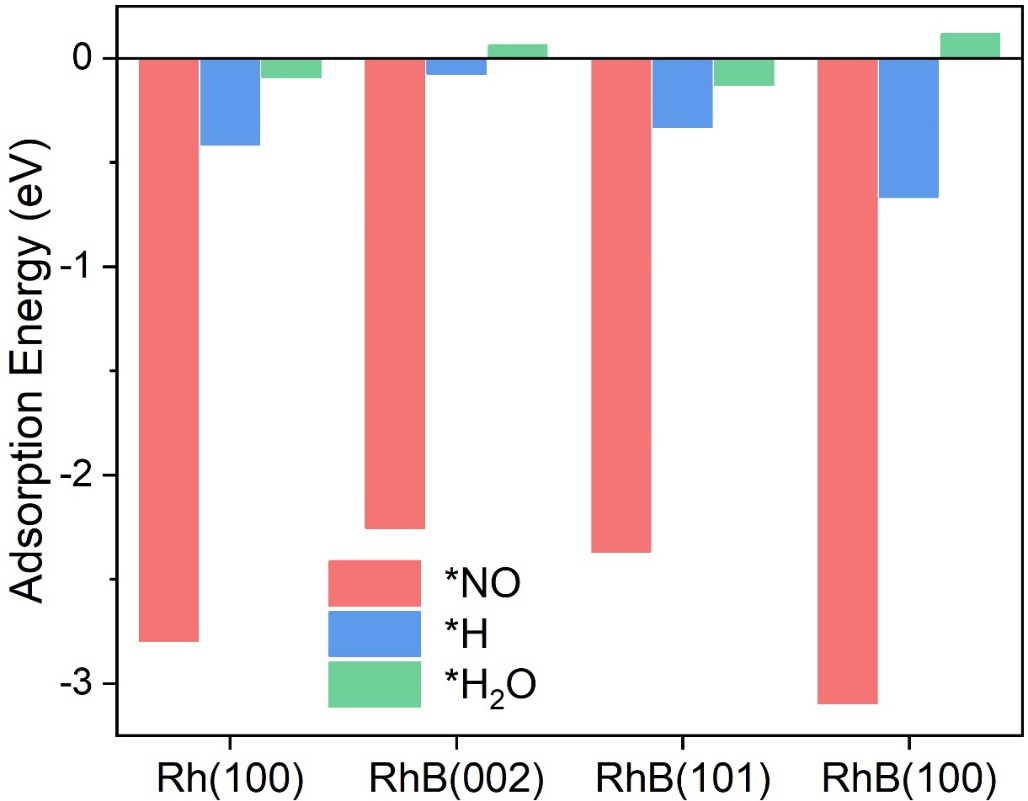


**Fig. S36** Calculated adsorption energies of *NO, *H, and *H_2_O on Rh(100), RhB(002), RhB(101), and RhB(100) facets


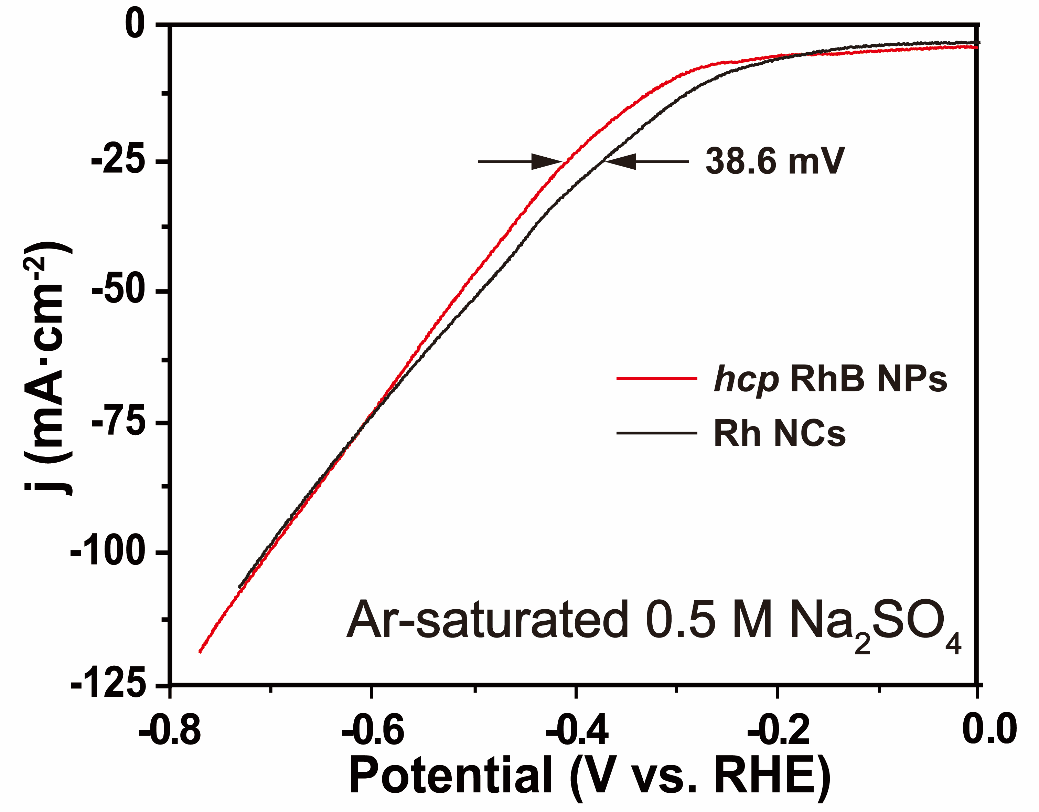


**Fig. S37** LSV curves of Rh NCs and *hcp* RhB NPs in Ar-saturated 0.5 M Na_2_SO_4_ electrolyte. The *hcp* RhB NPs exhibit a 38.6 mV higher overpotential than Rh NCs at the current density of −25 mA cm^−2^, demonstrating effective suppression of HER activity


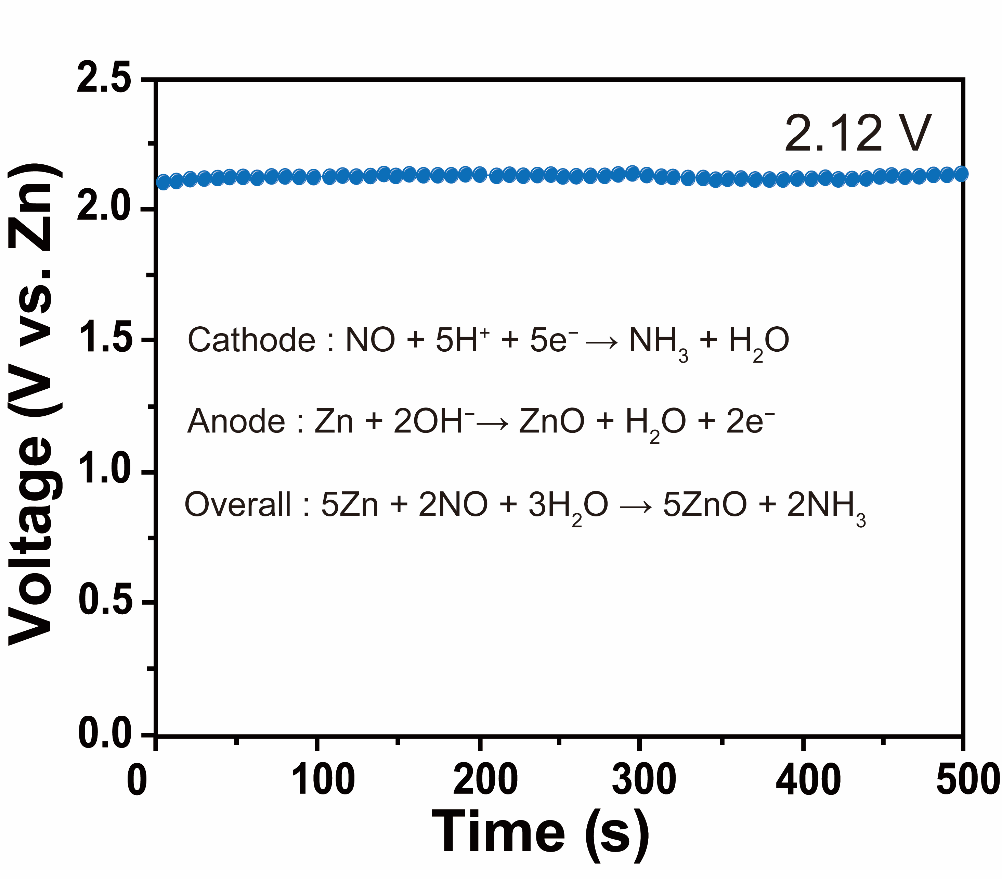


**Fig. S38** Open circuit voltage (OCV) measurement of the Zn-NO battery with *hcp* RhB NPs as the cathode

**
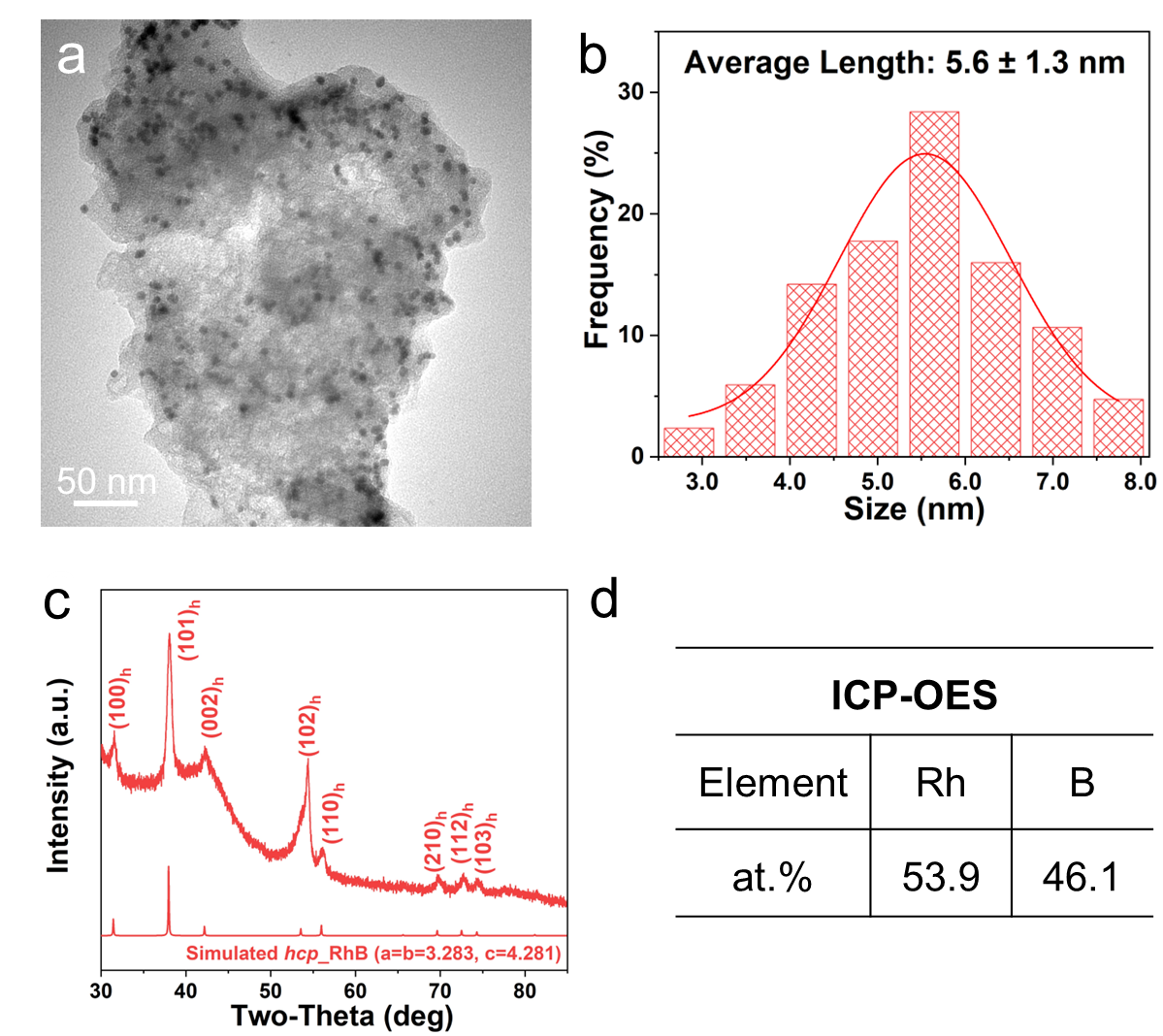
**

**Fig. S39** (**a**) TEM image, (**b**) Size distribution diagram, (**c**) XRD pattern, and (d) ICP-OES result of *hcp* RhB NPs after the Zn-NO battery test

# S3 Supplementary Tables

| Sample | Shell | C.N. | R (Å) | σ^2^ (×10^-3^ Å^2^) | *ΔE*_0_ (eV) | R-factor |
| --- | --- | --- | --- | --- | --- | --- |
| Rh foil | Rh-Rh | 12.0* | 2.72 | 3.6 | -8.6 | 0.005 |
| Rh_2_O_3_ | Rh-O | 6.0 | 2.04 | 4.0 | -3.7 | 0.011 |
|  | Rh-Rh | 3.0 | 3.05 | 11.3 |  |  |
| *hcp* RhB NPs | Rh-Rh | 8.7 ± 0.70 | 2.64 ± 0.05 | 7.9 ± 0.26 | -5.5 ± 1.05 | 0.018 |
|  | Rh-B | 5.3 ± 0.80 | 1.96 ± 0.04 | 2.0 ± 0.11 |  |  |
| Rh NCs | Rh-Rh | 10.4 ± 0.16 | 2.70 ± 0.03 | 3.8 ± 0.14 | -3.0 ± 0.35 | 0.007 |
| *a*-Rh_4_B NPs | / | / | / | / | / | / |

**Table S1** A summary of the Rh K-edge EXAFS fitting results of Rh NCs, *a*-Rh_4_B NPs, and *hcp* RhB NPs in reference to Rh foil and Rh_2_O_3_.

C.N.: coordination numbers; R: bond distance; σ^2^: Debye-Waller factors (a measure of thermal and static disorder in absorber-scatterer distances); *ΔE*_0_: the inner potential correction. R-factor: goodness of fit. *These values were fixed during the EXAFS fitting, based on the known structures of Rh foil.

**Table S2** Comparison of NH_3_ yield rate and FE_NH3_ of our catalysts with the reported nanocatalysts for electrocatalytic NORR performed in H-type electrochemical systems

| Catalysts | Electrolyte  (pH value) | *C*_NO_ | NH_3_ yield (µmol h^-1^ cm^-2^) | FE (%) | Potential  (V vs. RHE) | Ref. |
| --- | --- | --- | --- | --- | --- | --- |
| ***hcp* RhB NPs** | **0.5 M Na_2_SO_4_** (pH = 7.0) | 20 *vol.*% | **629.5** | **92.1** | **−0.60/−0.50^a^** | **This work** |
| **Rh NCs** | **0.5 M Na_2_SO_4_** (pH = 7.0) | 20 *vol.*% | **514.7** | **83.2** | **−0.60** | **This work** |
| ***a*-Rh_4_B NPs** | **0.5 M Na_2_SO_4_**  (pH = 7.0) | 20 *vol.*% | **403.8** | **73.7** | **−0.60/−0.50** | **This work** |
| Defective TiO_2−x_ | 0.2 M PBS  (pH = 7.4) | 10 *vol.*% | 72.41 | 92.5 | −0.70/−0.40 | Small 2023, 19, 2300291 [S6] |
| MoC/NCS | 0.1 M HCl + 0.5 mM Fe(II) sodium benzoate (Fe^II^-SB)  (pH = 1.0) | 99.99 *vol.*% | 79.27 | 89 | −0.80 | Nano Res. 2022,15, 8890 [S7] |
| CoB/Co@C | 0.5 M Na_2_SO_4_  (pH = 7.0) | 99.99 *vol.*% | 315.4 | 70.3 | −0.60 | Nano Lett. 2023, 23, 7120−7128 [S8] |
| NiO nanosheet | 0.1 M Na_2_SO_4_ + 0.5 mM Fe^II^-EDTA  (pH = 7.0) | 10 *vol.*% | 125.07 | 90.0 | −0.60 | Chem. Commun. 2021, 57, 13562–13565 [S9] |
| MoS_2_  nanosheet | 0.1 M HCl + 0.5 mM Fe^II^-SB  (pH = 1.0) | 10 *vol.*% | 99.6 | 76.6 | −0.70 | Angew. Chem. Int. Ed. 2021, 60, 25263–25268 [S10] |
| Ni_2_P nanosheet | 0.1 M HCl  (pH = 1.0) | 10 *vol.*% | 33.47 | 76.9 | −0.20 | J. Mater. Chem. A 2021, 9, 24268–24275 [S11] |
| *a-*B_2.6_C@TiO_2_ | 0.2 M Na_2_SO_4_ + Fe^II^-EDTA  (pH = 7.0) | 10 *vol.*% | 216.01 | 87.6 | −0.90 | Angew. Chem. Int. Ed. 2022, 61, e202202087 [S12] |
| Cu foam | 0.25 M Li_2_SO_4_  (pH = ~7.0) | 99.99 *vol.*% | 517.1 | 93.5 | −0.90 | Angew. Chem. Int. Ed. 2020, 59, 9711–9718 [S13] |
| Fe_2_O_3_ nanorod | 0.1 M Na_2_SO_4_ + 0.5 mM  Fe^II^-EDTA  (pH = ~7.0) | 10 *vol.*% | 78.02 | 86.7 | −0.60/−0.40 | J. Mater. Chem. A 2022, 10, 6454–6462 [S14] |
| CuFe alloy | 0.1 M PBS  (pH = 7.4) | 95 *vol.*% NO + 5 *vol.*%NO_2_ | 136.81 | 90.6 | −0.80 | Chem Catal. 2022, 2, 622-638 [S15] |
| CoP nanowire | 0.2 M Na_2_SO_4_  (pH = 7.0) | 10 *vol.*% | 47.22 | 88.3 | −0.20 | Inorg. Chem. Front. 2022, 9, 1366-1372 [S16] |
| Ru_0.05_Cu_0.95_ | 0.5 M Na_2_SO_4_  (pH = 7.0) | 25 *vol.*% | 17.68 | 64.9 | −0.50 | Sci. China Chem. 2021, 64, 1493 [S17] |
| Mo_2_C nanosheets | 0.5 M Na_2_SO_4_  (pH = 7.0) | 99.99 *vol.*% | 122.7 | 86.3 | −0.40 | Inorg. Chem. 2023, 62, 653−658 [S18] |
| *bcc* RuGa | 0.1 M K_2_SO_4_  (pH = 7.0) | 20 *vol.*% | 160.3 µmol h^-1^ cm^-2^ **^b^** | 72.3 | −0.20 | Angew. Chem. Int. Ed. 2023, 62, e202213351 [S19] |
| Cu@Co | 0.1 M Na_2_SO_4_  (pH = 7.0) | 1.0 *vol.*% | 36.89 | 76.5 | −0.50 | Adv. Mater. 2023, 36, e2309470 [S20] |
| Cu nanosheets | 0.1 M K_2_SO_4_  (pH = 7.0) | 99.99 *vol.*% | 187.5 | 93.2 | −0.59 | Angew. Chem. Int. Ed. 2024, 63, e202319135 [S21] |
| FeOCl-V_Cl_ | 0.1 M HCl +  50 mM sodium benzenesulfonate (SB)  (pH = 1.0) | 1.0 *vol.*% | 26.79 | 91.1 | −0.50 | Angew. Chem. Int. Ed. 2024, 63, e202318792 [S22] |
| CuFe DS/NC | 0.1 M Na_2_SO_4_  (pH = 7.0) | 99.99 *vol.*% | 112.52 | 90.0 | −0.60 | Adv. Mater. 2023, 35, 2304646 [S23] |
| *hcp* Co | 0.1 M Na_2_SO_4_  (pH = 7.0) | 99.99 *vol.*% | 439.5 | 72.6 | −0.60 | J. Am. Chem. Soc. 2023, 145, 6899−6904 [S24] |
|  | 0.1 M Na_2_SO_4_  + 0.5 mM Fe^II^-EDTA  (pH = 7.0) | 99.99 *vol.*% | 463.4 | 75.8 | −0.60 |  |
| low-coordinated Ru | 0.5 M Na_2_SO_4_  (pH = 7.0) | 1.0 *vol.*% | 45.02 | 66.0 | −0.20 | ACS Energy Lett. 2022, 7, 1187−1194 [S25] |
| MnO_2-x_ nanowire array | 0.2 M Na_2_SO_4_  (pH = 7.0) | 10 *vol.*% | 9.90 | 82.8 | −0.70 | Mater. Today Phys. 2022, 22, 100586 [S26] |
| FeNC | 0.1 M HClO_4_  (pH = 1.0) | 10 *vol.*% | ~20.2 | 5.1 | −0.20 | Nat. Commun. 2021, 12, 1856 [S27] |
| NiNC@CF | 0.5 M PBS  (pH = 7.4) | 99.99 *vol.*% | 108 | 87.0 | −0.90/−0.50 | J. Mater. Chem. A 2022, 10, 6470 [S28] |
| FeB_2_ | 0.5 M Na_2_SO_4_  (pH = 7.0) | 99.9 *vol.*% | 289.3 | 93.8 | −0.40 | Inorg. Chem. 2023, 62, 8487−8493 [S29] |
| Cu_1_/MoS_2_ | 0.5 M Na_2_SO_4_  (pH = 7.0) | 99.99 *vol.*% | 337.5 | 90.6 | −0.60 | Nano Res. 2023, 16, 5857–5863 [S30] |
| Sb_1_/MoO_3_ | 0.5 M Na_2_SO_4_  (pH = 7.0) | 99.9 *vol.*% | 273.5 | 91.7 | −0.60 | ACS Energy Lett. 2023, 8, 1281−1288 [S31] |
| Pd_1_Cu | 0.5 M Na_2_SO_4_  (pH = 7.0) | 99.99 *vol.*% | 305.8 | 96.7 | −0.40 | Nano Lett. 2024, 24, 541-548 [S32] |
| Ir_1_/MoO_3_ | 0.5 M Na_2_SO_4_  (pH = 7.0) | 99.9 *vol.*% | 438.8 | 93.2 | −0.47 | Nano Res. 2023, 16, 8737-8742 [S33] |
| Fe_1_/MoS_2-x_ | 0.5 M Na_2_SO_4_  (pH = 7.0) | 99.99 *vol.*% | 288.2 | 82.5 | −0.60 | Appl. Catal., B 2023, 324, 122241 [S34] |
| W_1_/MoO_3−x_ | 0.5 M Na_2_SO_4_  (pH = 7.0) | 99.99 *vol.*% | 308.6 | 91.2 | −0.50/−0.40 | Nano Lett. 2023, 23, 1735-1742 [S35] |
| Cu@Cu/C NWAs | 0.05 M H_2_SO_4_  (pH = 7.0) | 3.0 *vol.*% | 69.3 | 93.0 | −0.10 | J. Am. Chem. Soc. 2024, 146, 10044 [S36] |
| V_O_-TiO_2-x_ | 1 M KOH +  50 mM SB  (pH = 14.0) | 1.0 *vol.*% | 37.2 µmol h^-1^ mg_cat._^-1^ **^c^** | 76.4 | −0.50 | Angew. Chem. Int. Ed. 2024, e202420346 [S37] |

**a**: This catalyst achieves the maximum NH_3_ yield rate and FE_NH3_ at different potential.

**b**: The NH_3_ yield rate of *bcc* RuGa was re-calculated in the unit of µmol h^-1^ cm^-2^.

**c**: The NH_3_ yield rate of V_O_-TiO_2-x_ was calculated in the unit of µmol h^-1^ mg_cat._^-1^.

**Table S3** The NO adsorption energy (*E*_ads_) of different configurations on Rh(100), RhB(002), RhB(100) and RhB(101) facets

| Model | Adsorption Site | NO adsorption energy (*E*_ads_) |
| --- | --- | --- |
| Rh(100) | RhN_t_ | -2.429 eV |
|  | **Rh_2_N_b_** | **-2.797 eV** |
|  | Rh_4_N_h_ | -2.640 eV |
| RhB(002) | RhN_t_ | -2.136 eV |
|  | Rh_2_N_b_ | -2.088 eV |
|  | **Rh_3_N_f_** | **-2.255 eV** |
|  | Rh_3_N_h_ | -2.088 eV |
| RhB(101) | RhN_t_ | -2.063 eV |
|  | Rh_2_N_b_ | -1.806 eV |
|  | Rh_3_N_h_ | -1.806 eV |
|  | **Rh_3_N_f_** | **-2.371 eV** |
| RhB(100) | RhN_t_ | -2.334 eV |
|  | Rh_2_N_b_ | -2.988 eV |
|  | **Rh_3_N_h_** | **-3.097 eV** |
|  | Rh_3_N_f_ | -2.350 eV |

Note: The *E*_ads_ calculation results reveal that NO prefers to adopt Rh_3_N_f_ sites on RhB(002) and RhB(101), Rh_3_N_h_ site on RhB(100), and Rh_2_N_b_ site on Rh(100).

**Table S4** Adsorbent charge transfer in Bader analysis (+ represents adsorbate gain electrons from slab, − is the opposite)

| System | Rh(100) | RhB(002) | RhB(100) | RhB(101) |
| --- | --- | --- | --- | --- |
| Sum electrons | +0.37 e^−^ | +0.47 e^−^ | +0.56 e^−^ | +0.44 e^−^ |

**Table S5** Calculated Gibbs free energies (ΔG) of adsorption species for NORR on Rh(100), RhB(002), RhB(100) and RhB(101) surfaces with respect to the reference NO + * (in eV)

| Rh (100) | * | *NO | ***HNO** | *NHOH | *NH_2_OH | *NH_2_ | *NH_3_ |
| --- | --- | --- | --- | --- | --- | --- | --- |
|  | 0.00 | -2.253 | **-1.677** | -1.370 | -1.910 | -4.078 | -4.242 |
|  | * | *NO | ***NOH** |  |  |  |  |
|  | 0.00 | -2.253 | **-1.590** |  |  |  |  |
| RhB (002) | * | *NO | ***HNO** | *NHOH | *NH_2_OH | *NH_2_ | *NH_3_ |
|  | 0.00 | -1.693 | **-1.214** | -1.443 | -1.725 | -3.476 | -4.001 |
|  | * | *NO | ***NOH** |  |  |  |  |
|  | 0.00 | -1.693 | **-0.882** |  |  |  |  |
| RhB (101) | * | *NO | ***HNO** | *NHOH | *NH_2_OH | *NH_2_ | *NH_3_ |
|  | 0.00 | -1.807 | **-1.396** | -1.410 | -1.730 | -3.675 | -4.245 |
|  | * | *NO | ***NOH** |  |  |  |  |
|  | 0.00 | -1.807 | **-1.295** |  |  |  |  |
| RhB (100) | * | *NO | ***HNO** |  |  |  |  |
|  | 0.00 | -2.824 | **-2.104** |  |  |  |  |
|  | * | *NO | ***NOH** | *NHOH | *NH_2_OH | *NH_2_ | *NH_3_ |
|  | 0.00 | -2.824 | **-2.130** | -1.970 | -1.856 | -4.062 | -4.121 |

**Table S6** Comparison of peak power density of our battery with the reported Zn-NO battery systems

| Catalysts | Catholyte | Power density (mW cm^-2^) | Refs. |
| --- | --- | --- | --- |
| ***hcp* RhB NPs** | **0.2 M Na_2_SO_4_** | **4.33** | **This work** |
| **Rh NCs** | **0.2 M Na_2_SO_4_** | **2.91** | **This work** |
| ***a*-Rh_4_B NPs** | **0.2 M Na_2_SO_4_** | **1.94** | **This work** |
| NiO nanosheet | 0.1 M Na_2_SO_4_ | 0.88 | Chem. Commun. 2021, 57, 13562−13565 [S9] |
| MoS_2_ nanosheet | 0.1 M HCl + 0.5 mM Fe^II^-SB**^a^** | 1.04 | Angew. Chem. Int. Ed. 2021, 60, 25263–25268 [S10] |
| Fe_2_O_3_ nanorod | 0.1 M Na_2_SO_4_ | 1.18 | J. Mater. Chem. A, 2022, 10, 6454–6462 [S14] |
| Ni_2_P nanosheet | 0.1 M HCl | 1.53 | J. Mater. Chem. A, 2021, 9, 24268–24275 [S11] |
| *a-*B_2.6_C@TiO_2_ | 0.1 M Na_2_SO_4_ | 1.7 | Angew. Chem. Int. Ed. 2022, 61, e202202087 [S12] |
| CuFe DS/NC | 0.1 M Na_2_SO_4_ | 2.3 | Adv. Mater. 2023, 35, 2304646 [S23] |
| Cu@Co | 0.1 M Na_2_SO_4_ | 3.08 | Adv. Mater. 2023, 2309470 [S20] |
| CoB/Co@C | 0.5 M Na_2_SO_4_ | 3.68 | Nano Lett. 2023, 23, 7120−7128 [S8] |
| *hcp* Co | 0.1 M Na_2_SO_4_ | 4.66 | J. Am. Chem. Soc. 2023, 145, 6899−6904 [S24] |
| FeOCl-V_Cl_ | 0.1 M HCl + 50 mM SB**^a^** | 6.2 | Angew. Chem. Int. Ed. 2024, e202318792 [S22] |
| NiFe LDH | 0.1 M HCl + 0.5 mM Fe^II^-SB**^a^** | 1.8 | Chem. Commun. 2022, 58, 8097–8100 [S38] |
| Bi NDs | 0.1 M Na_2_SO_4_  + 0.5 mM Fe^II^-EDTA**^a^** | 2.33 | Mater. Today Phys. 2022, 22, 100611 [S39] |
| Bi NPs@C | 0.1 M Na_2_SO_4_ | 2.35 | Nano Res. 2022, 151 5032-5037 [S40] |
| CoP nanowire | 0.2 M Na_2_SO_4_ | 0.496 | Inorg. Chem. Front. 2022, 9, 1366-1372 [S16] |
| MoC/NCS | 0.1 M HCl + 0.5 mM Fe^II^-SB**^a^** | 1.8 | Nano Res. 2022,15, 8890-8896 [S7] |
| CoS_x_ nanosheet | 0.2 M Na_2_SO_4_ | 2.06 | Inorg. Chem. 2022, 61, 8096−8102 [S41] |

**a**: Fe^II^-SB, SB, and Fe^II^-EDTA are electrolyte additives for rapid NO capture [S42, S43].

**Supplementary References**

1. D. Zhu, L. Zhang, R.E. Ruther, R.J. Hamers, Photo-illuminated diamond as a solid-state source of solvated electrons in water for nitrogen reduction. Nat. Mater. **12**(9), 836–841 (2013). <https://doi.org/10.1038/nmat3696>
2. G.W. Watt, J.D. Chrisp, Spectrophotometric method for determination of hydrazine. Anal. Chem. **24**(12), 2006–2008 (1952). <https://doi.org/10.1021/ac60072a044>
3. B. Aronsson, J. Åselius, E. Stenberg, Borides and silicides of the platinum metals. Nature **183**(4671), 1318–1319 (1959). <https://doi.org/10.1038/1831318b0>
4. Z. Li, X. Ai, H. Chen, X. Liang, X. Li et al., Asymmetrically strained hcp rhodium sublattice stabilized by 1D covalent boron chains as an efficient electrocatalyst. Chem. Commun. **57**(41), 5075–5078 (2021). <https://doi.org/10.1039/D1CC00774B>
5. D. Chen, R. Yu, H. Zhao, J. Jiao, X. Mu et al., Boron-induced interstitial effects drive water oxidation on ordered Ir−B compounds. Angew. Chem. Int. Ed. **63**(35), e202407577 (2024). <https://doi.org/10.1002/anie.202407577>
6. Z. Li, Q. Zhou, J. Liang, L. Zhang, X. Fan et al., Defective TiO_2_−x for high-performance electrocatalytic NO reduction toward ambient NH_3_ production. Small **19**(24), 2300291 (2023). <https://doi.org/10.1002/smll.202300291>
7. G. Meng, M. Jin, T. Wei, Q. Liu, S. Zhang et al., MoC nanocrystals confined in N-doped carbon nanosheets toward highly selective electrocatalytic nitric oxide reduction to ammonia. Nano Res. **15**(10), 8890–8896 (2022). <https://doi.org/10.1007/s12274-022-4747-y>
8. B. Wu, L. Huang, L. Yan, H. Gang, Y. Cao et al., Boron-modulated electronic-configuration tuning of cobalt for enhanced nitric oxide fixation to ammonia. Nano Lett. **23**(15), 7120–7128 (2023). <https://doi.org/10.1021/acs.nanolett.3c01994>
9. P. Liu, J. Liang, J. Wang, L. Zhang, J. Li et al., High-performance NH_3_ production *via* NO electroreduction over a NiO nanosheet array. Chem. Commun. **57**(99), 13562–13565 (2021). <https://doi.org/10.1039/D1CC06113E>
10. L. Zhang, J. Liang, Y. Wang, T. Mou, Y. Lin et al., High-performance electrochemical NO reduction into NH_3_ by MoS_2_ nanosheet. Angew. Chem. Int. Ed. **60**(48), 25263–25268 (2021). <https://doi.org/10.1002/anie.202110879>
11. T. Mou, J. Liang, Z. Ma, L. Zhang, Y. Lin et al., High-efficiency electrohydrogenation of nitric oxide to ammonia on a Ni2P nanoarray under ambient conditions. J. Mater. Chem. A **9**(43), 24268–24275 (2021). <https://doi.org/10.1039/D1TA07455E>
12. J. Liang, P. Liu, Q. Li, T. Li, L. Yue et al., Amorphous boron carbide on titanium dioxide nanobelt arrays for high-efficiency electrocatalytic NO reduction to NH_3_. Angew. Chem. **134**(18), e202202087 (2022). <https://doi.org/10.1002/ange.202202087>
13. J. Long, S. Chen, Y. Zhang, C. Guo, X. Fu et al., Direct electrochemical ammonia synthesis from nitric oxide. Angew. Chem. Int. Ed. **59**(24), 9711–9718 (2020). <https://doi.org/10.1002/anie.202002337>
14. J. Liang, H. Chen, T. Mou, L. Zhang, Y. Lin et al., Coupling denitrification and ammonia synthesis *via* selective electrochemical reduction of nitric oxide over Fe_2_O_3_ nanorods. J. Mater. Chem. A **10**(12), 6454–6462 (2022). <https://doi.org/10.1039/D2TA00744D>
15. R. Hao, L. Tian, C. Wang, L. Wang, Y. Liu et al., Pollution to solution: a universal electrocatalyst for reduction of all NO*_x_*-based species to NH_3_. Chem Catal. **2**(3), 622–638 (2022). <https://doi.org/10.1016/j.checat.2022.01.022>
16. J. Liang, W.-F. Hu, B. Song, T. Mou, L. Zhang et al., Efficient nitric oxide electroreduction toward ambient ammonia synthesis catalyzed by a CoP nanoarray. Inorg. Chem. Front. **9**(7), 1366–1372 (2022). <https://doi.org/10.1039/D2QI00002D>
17. J. Shi, C. Wang, R. Yang, F. Chen, N. Meng et al., Promoting nitric oxide electroreduction to ammonia over electron-rich Cu modulated by Ru doping. Sci. China Chem. **64**(9), 1493–1497 (2021). <https://doi.org/10.1007/s11426-021-1073-5>
18. K. Chen, P. Shen, N. Zhang, D. Ma, K. Chu, Electrocatalytic NO reduction to NH_3_ on Mo_2_C nanosheets. Inorg. Chem. **62**(2), 653–658 (2023). <https://doi.org/10.1021/acs.inorgchem.2c03714>
19. H. Zhang, Y. Li, C. Cheng, J. Zhou, P. Yin et al., Isolated electron-rich ruthenium atoms in intermetallic compounds for boosting electrochemical nitric oxide reduction to ammonia. Angew. Chem. Int. Ed. **62**(4), e202213351 (2023). <https://doi.org/10.1002/anie.202213351>
20. Z. Wu, Y. Liu, D. Wang, Y. Zhang, K. Gu et al., Cu@Co with dilatation strain for high-performance electrocatalytic reduction of low-concentration nitric oxide. Adv. Mater. **36**(11), e2309470 (2024). <https://doi.org/10.1002/adma.202309470>
21. L. Xiao, S. Mou, W. Dai, W. Yang, Q. Cheng et al., Identification of Cu(111) as superior active sites for electrocatalytic NO reduction to NH(3) with high single-pass conversion efficiency. Angew. Chem. Int. Ed. **63**(11), e202319135 (2024). <https://doi.org/10.1002/anie.202319135>
22. X. Guo, P. Wang, T. Wu, Z. Wang, J. Li et al., Aqueous electroreduction of nitric oxide to ammonia at low concentration *via* vacancy engineered FeOCl. Angew. Chem. Int. Ed. **63**(6), e202318792 (2024). <https://doi.org/10.1002/anie.202318792>
23. D. Wang, X. Zhu, X. Tu, X. Zhang, C. Chen et al., Oxygen-bridged copper–iron atomic pair as dual-metal active sites for boosting electrocatalytic NO reduction. Adv. Mater. **35**(39), 2304646 (2023). <https://doi.org/10.1002/adma.202304646>
24. D. Wang, Z.-W. Chen, K. Gu, C. Chen, Y. Liu et al., Hexagonal cobalt nanosheets for high-performance electrocatalytic NO reduction to NH_3_. J. Am. Chem. Soc. **145**(12), 6899–6904 (2023). <https://doi.org/10.1021/jacs.3c00276>
25. Y. Li, C. Cheng, S. Han, Y. Huang, X. Du et al., Electrocatalytic reduction of low-concentration nitric oxide into ammonia over Ru nanosheets. ACS Energy Lett. **7**(3), 1187–1194 (2022). <https://doi.org/10.1021/acsenergylett.2c00207>
26. Z. Li, Z. Ma, J. Liang, Y. Ren, T. Li et al., MnO_2_ nanoarray with oxygen vacancies: an efficient catalyst for NO electroreduction to NH_3_ at ambient conditions. Mater. Today Phys. **22**, 100586 (2022). <https://doi.org/10.1016/j.mtphys.2021.100586>
27. D.H. Kim, S. Ringe, H. Kim, S. Kim, B. Kim et al., Selective electrochemical reduction of nitric oxide to hydroxylamine by atomically dispersed iron catalyst. Nat. Commun. **12**(1), 1856 (2021). <https://doi.org/10.1038/s41467-021-22147-7>
28. T. Muthusamy, S. Sethuram Markandaraj, S. Shanmugam, Nickel nanoparticles wrapped in N-doped carbon nanostructures for efficient electrochemical reduction of NO to NH_3_. J. Mater. Chem. A **10**(12), 6470–6474 (2022). <https://doi.org/10.1039/D2TA00623E>
29. G. Zhang, F. Wang, Y. Wan, Y. Guo, K. Chu, Iron diboride (FeB2) for the electroreduction of NO to NH_3_. Inorg. Chem. **62**(22), 8487–8493 (2023). <https://doi.org/10.1021/acs.inorgchem.3c01207>
30. K. Chen, G. Zhang, X. Li, X. Zhao, K. Chu, Electrochemical NO reduction to NH_3_ on Cu single atom catalyst. Nano Res. **16**(4), 5857–5863 (2023). <https://doi.org/10.1007/s12274-023-5384-9>
31. K. Chen, Y. Zhang, J. Xiang, X. Zhao, X. Li et al., P-block antimony single-atom catalysts for nitric oxide electroreduction to ammonia. ACS Energy Lett. **8**(3), 1281–1288 (2023). <https://doi.org/10.1021/acsenergylett.2c02882>
32. K. Chen, J. Xiang, Y. Guo, X. Liu, X. Li et al., Pd_1_Cu single-atom alloys for high-current-density and durable NO-to-NH_3_ electroreduction. Nano Lett. **24**(2), 541–548 (2024). <https://doi.org/10.1021/acs.nanolett.3c02259>
33. K. Chen, G. Wang, Y. Guo, D. Ma, K. Chu, Iridium single-atom catalyst for highly efficient NO electroreduction to NH_3_. Nano Res. **16**(7), 8737–8742 (2023). <https://doi.org/10.1007/s12274-023-5556-7>
34. K. Chen, J. Wang, J. Kang, X. Lu, X. Zhao et al., Atomically Fe-doped MoS_2−_*_x_* with Fe-Mo dual sites for efficient electrocatalytic NO reduction to NH_3_. Appl. Catal. B Environ. **324**, 122241 (2023). <https://doi.org/10.1016/j.apcatb.2022.122241>
35. K. Chen, J. Wang, H. Zhang, D. Ma, K. Chu, Self-tandem electrocatalytic NO reduction to NH_3_ on a W single-atom catalyst. Nano Lett. **23**(5), 1735–1742 (2023). <https://doi.org/10.1021/acs.nanolett.2c04444>
36. J. Meng, C. Cheng, Y. Wang, Y. Yu, B. Zhang, Carbon support enhanced mass transfer and metal–support interaction promoted activation for low-concentrated nitric oxide electroreduction to ammonia. J. Am. Chem. Soc. **146**(14), 10044–10051 (2024). <https://doi.org/10.1021/jacs.4c00898>
37. X. Guo, T. Wu, H. Li, L. Chai, M. Liu, Enhancing low-concentration electroreduction of NO to NH_3_ *via* potential-controlled active site-intermediate interactions. Angew. Chem. Int. Ed. **64**(8), e202420346 (2025). <https://doi.org/10.1002/anie.202420346>
38. G. Meng, T. Wei, W. Liu, W. Li, S. Zhang et al., NiFe layered double hydroxide nanosheet array for high-efficiency electrocatalytic reduction of nitric oxide to ammonia. Chem. Commun. **58**(58), 8097–8100 (2022). <https://doi.org/10.1039/D2CC02463B>
39. Y. Lin, J. Liang, H. Li, L. Zhang, T. Mou et al., Bi nanodendrites for highly efficient electrocatalytic NO reduction to NH_3_ at ambient conditions. Mater. Today Phys. **22**, 100611 (2022). <https://doi.org/10.1016/j.mtphys.2022.100611>
40. Q. Liu, Y. Lin, L. Yue, J. Liang, L. Zhang et al., Bi nanoparticles/carbon nanosheet composite: a high-efficiency electrocatalyst for NO reduction to NH_3_. Nano Res. **15**(6), 5032–5037 (2022). <https://doi.org/10.1007/s12274-022-4283-9>
41. L. Zhang, Q. Zhou, J. Liang, L. Yue, T. Li et al., Enhancing electrocatalytic NO reduction to NH_3_ by the CoS nanosheet with sulfur vacancies. Inorg. Chem. **61**(20), 8096–8102 (2022). <https://doi.org/10.1021/acs.inorgchem.2c01112>
42. K. Ogura, H. Ishikawa, Electrochemical conversion of nitrous oxide into ammonia in the presence of iron complexes. J. Chem. Soc., Faraday Trans. 1 **80**(8), 2243 (1984). <https://doi.org/10.1039/f19848002243>
43. E.K. Pham, S.-G. Chang, Removal of NO from flue gases by absorption to an iron(ii) thiochelate complex and subsequent reduction to ammonia. Nature **369**(6476), 139–141 (1994). <https://doi.org/10.1038/369139a0>
